# Metabolic labelling of the carbohydrate core in bacterial peptidoglycan and its applications

**DOI:** 10.1038/ncomms15015

**Published:** 2017-04-20

**Authors:** Hai Liang, Kristen E. DeMeester, Ching-Wen Hou, Michelle A. Parent, Jeffrey L. Caplan, Catherine L. Grimes

**Affiliations:** 1 Department of Chemistry and Biochemistry, University of Delaware, 134 Brown Lab, Newark, Delaware 19716, USA; 2 Department of Medical Laboratory Sciences, University of Delaware, Newark, Delaware 19716, USA; 3 Department of Biological Sciences, University of Delaware, Newark, Delaware 19716, USA; 4 Bioimaging Center, Delaware Biotechnology Institute, Newark, Delaware 19716, USA

## Abstract

Bacterial cells are surrounded by a polymer known as peptidoglycan (PG), which protects the cell from changes in osmotic pressure and small molecule insults. A component of this material, *N*-acetyl-muramic acid (NAM), serves as a core structural element for innate immune recognition of PG fragments. We report the synthesis of modifiable NAM carbohydrate derivatives and the installation of these building blocks into the backbone of Gram-positive and Gram-negative bacterial PG utilizing metabolic cell wall recycling and biosynthetic machineries. Whole cells are labelled via click chemistry and visualized using super-resolution microscopy, revealing higher resolution PG structural details and allowing the cell wall biosynthesis, as well as its destruction in immune cells, to be tracked. This study will assist in the future identification of mechanisms that the immune system uses to recognize bacteria, glean information about fundamental cell wall architecture and aid in the design of novel antibiotics.

## Abstract (web summary)

*N*-acetyl-muramic acid (NAM) is a core component of the bacterial peptidoglycan (PG) cell wall, and is recognised by the innate immune system. Here the authors engineer Gram-negative and Gram-positive bacteria to incorporate a modified NAM into the backbone of PG, which can be labelled with click chemistry for imaging and tracking.

Peptidoglycan (PG), a component of bacterial cell wall, is one of the essential polymers for life. Bacterial cells are surrounded by this material, which assists in bacterial cell division, maintenance of cell shape and small molecule recognition and signalling^1^,^2^. PG is composed of alternating monosaccharide units *N*-acetyl glucosamine (NAG) and *N*-acetyl muramic acid (NAM), with short peptide chains present on the muramic acid residue^3^. These molecular building blocks can combine in a variety of ways to produce a range of macromolecular structures, with NAG and NAM remaining constant throughout all bacteria^3^. PG structures impact human health, as antibiotics are designed to target its destruction^4^, whereas the innate immune system senses and responds to bacteria via fragments of PG^5^.

The innate immune system, the first line of defense against pathogens^6^, must differentiate and control ∼39 trillion bacteria that constitute the microbiome^7^. Commensal and pathogenic bacteria produce PG fragments, many of which contain the core glycan unit NAM^8^. The innate immune system utilizes a series of receptors, including Toll-like receptors and Nod-like receptors^9^,^10^,^11^, which bind to fragments of PG, such as the synthetic fragments muramyl dipeptide (MDP) and muramyl tripeptide (MTP), to generate the proper immune response^12^,^13^,^14^ (Fig. 1a). Moreover, misrecognition of PG fragments can lead to the development of inflammatory bowel disease, such as Crohn's disease, asthma and gastrointestinal cancers^15^,^16^. Information regarding the biological identity and the generation^17^,^18^ of these NAM-containing fragments, either via host or pathogen machinery, and the cellular location of these events is limited^19^,^20^,^21^,^22^. Tracking the biologically relevant NAM fragments of bacterial cell wall that are naturally produced proves challenging without a NAM-based set of molecular probes. To answer questions regarding both immune recognition and the three-dimensional (3D) architecture of bacterial cell walls, we developed the synthesis of the necessary PG building blocks, subsequent incorporation strategies and a method to label and visualize the glycan backbone directly.

To strategically develop a NAM-based labelling method, we first took inspiration from bacterial PG composition and biosynthesis. The biosynthesis of PG and its molecular intermediates have been known and well characterized since the late 1950s (refs 2, 23). The PG polymer to be modified is highly cross-linked and its synthesis is conserved, involving more than ten distinct enzymatic steps (Fig. 1b). Motivation to utilize the biosynthetic machinery to tag this polymer was driven from enormous efforts and successes in *in vivo* labelling of macromolecular structures and PG. Pioneering work conducted by Bertozzi and Kiessling^24^ introduced bioorthogonal functionality into eukaryotic glycans. These studies showcased the power of glycoengineering and subsequent chemical manipulation in whole cells. We were interested in applying these fundamental principles to bacterial PG and gathered inspiration from previous efforts to label this polymer: unnatural amino acids including D-amino acid fluorophores and derivatives can be incorporated using metabolic machinery, cell wall targeting antibiotics can deliver probes and proteins embedded in the cell wall can be modified to include a fluorescent dye^25^,^26^,^27^,^28^,^29^,^30^,^31^,^32^,^33^,^34^,^35^,^36^. Furthermore, efforts by Nishimura and colleagues^37^ revealed that the NAG unit of PG could potentially be labelled at the 2-*N* acetyl position in lactic acid bacteria. These elegant methods have proven useful in studying bacterial cell wall. However, current methods that label the terminal D-Ala residues of the peptide stems are subject to removal during PG remodelling and these terminal residues are not required for immune activation^14^. For example, MDP and MTP (Fig. 1a) do not contain a D-Ala residue. Moreover, extension of the peptide destroys the ability for the fragments to activate innate immune receptors *in vitro*^13^. However, the label at the NAM carbohydrate level will be retained in predicted innate immune agonist structures (MDP/MTP, Fig. 1a)^5^,^13^,^14^. Unfortunately, all of the aforementioned methods do not label on the NAM carbohydrate core of the PG polymer. To overcome this concern, we sought to install bioorthogonal functionality on the NAM carbohydrate backbone, which would increase the lifetime of the probe and allow the study of nascent polymer through metabolic labelling. D-Ala probes can be incorporated by exchange reactions^28^ in mature cell wall, as well as through a biosynthetic route complicating efforts to measure sites of *de novo* PG synthesis. Furthermore, a NAM-based labelling strategy would allow for the selective incorporation of label into NAM residues, which are only found in bacterial PG^5^.

When designing the NAM-based probes, we were inspired by nature, as mycobacteria and Actinomycetes can modify the 2-*N* acetyl position of the NAM glycan to *N*-glycolyl via NAM hydroxylase^38^, revealing that unnatural structural features at the *N*-acetyl position may be tolerated by the PG biosynthetic machinery. Although *N*-glycolylation has been responsible for an increase in immunogenicity^39^, we have recently shown that small modifications such as an azide and biotin, at the 2-*N* acetyl position do not have a significant effect on innate immune activation, specifically Nod2-dependent nuclear factor-κB signalling^40^, indicating a tolerance of these bioorthogonal modifications that allow for manipulation at this position to study innate immune signalling. Here we present a new, complementary method to remodel and label the 2-*N* position on the NAM residue and showcase its utility to visualize this essential structure in multiple organisms and monitor the fragmentation of PG after macrophage uptake.

## Results

### Synthesis of NAM derivatives and utilization by PG enzymes

**Figure 1:**
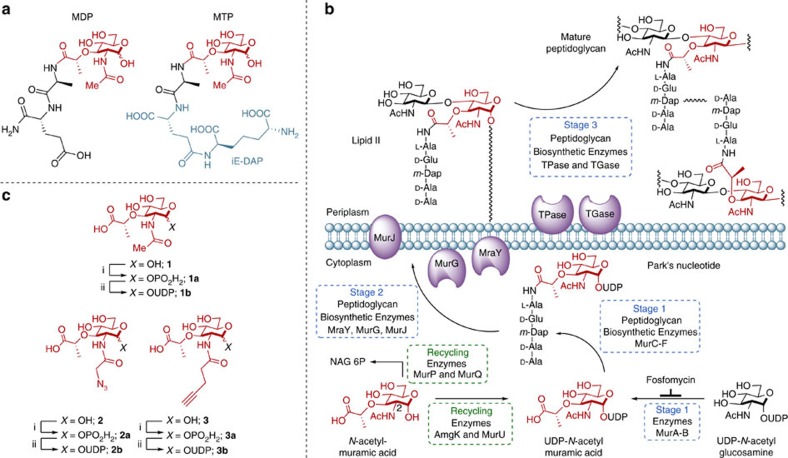
Immunostimulatory PG fragments and use of bioorthogonal NAM derivatives in PG Biosynthesis. (**a**) Synthetic fragments MDP (*N*-acetylmuramic acid linked by its lactic acid moiety to the N terminus of an L-alanine D-isoglutamine dipeptide) and MTP (MTP with the D-iso-glutamine-diaminopimelic acid (iE-DAP) moiety shown in blue) of PG used to probe the innate immune response. (**b**) PG biosynthesis begins with the formation of UDP-MurNAc through MurA/B and UDP-NAG (UDP-GlcNAc). Recycling enzymes AmgK/MurU provide another route to synthesize UDP-MurNAc with NAM as the building block. NAM could be further metabolized into NAG 6-phosphate (NAG 6P) via enzymes MurP and MurQ. UDP-MurNAc is converted into Park's nucleotide through enzymes MurC-F. MraY links Park's nucleotide to the cell membrane where MurG then glycosylates this fragment to form Lipid II. MurJ transports Lipid II into the periplasmic space where transglycosylases (TGase) and transpeptidases (TPase) further cross-link these molecules to form the mature PG. (**c**) Bioorthogonal NAM (**1**) derivatives with azide (**2**) and alkyne (**3**) modifications: (i) AmgK and (ii) MurU.

**Figure 2:**
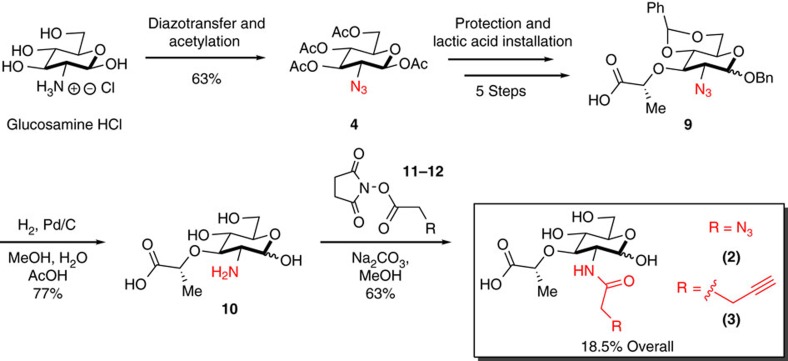
Modular synthesis of bioorthogonal NAM derivatives. The azide was installed onto glucosamine HCl using a diazotransfer followed by acetylation to yield **4**. Intermediate **4** was protected and modified over 5 steps: (i) hydrazine acetate; (ii) COCl_2_ followed by AgCO_3_, AgOTf and BnOH; (iii) 0.5 M NaOMe; (iv) PhCH(OMe)_2_, pTSA; (v) 60% NaH, *(s)*-2-chloropropionic acid) to yield **9**. Global deprotection via Pd/C hydrogenation produced **10**. Followed by NHS coupling conditions, **2** and **3** are synthesized in 18.5% overall yield from the commercially available glucosamine HCl.

The fundamental building blocks of bacterial cell wall are the glycans NAG and NAM. The latter is exclusively used as a PG building block (Fig. 1b). The NAM monosaccharide is introduced during the first committed step to bacterial cell wall biosynthesis with the formation of UDP-*N*-acetyl-muramic acid (UDP-MurNAc) (Fig. 1b). The bioorthogonal handle was installed at this intermediate. To circumvent the synthesis of the gram quantities of the UDP-MurNAc derivatives and complicated delivery strategies for the diphosphate moiety, we utilized cell wall recycling machinery, which is absent in *Escherichia coli*, but present in many bacterial species including *Pseudomonas putida*^41^. The recycling enzyme anomeric NAM/NAG kinase (AmgK) converts NAM **1** into MurNAc 1-phosphate **1a**, which is then converted to UDP-MurNAc **1b** by MurNAc α-1-phosphate uridylyl transferase (MurU) (Fig. 1b,c). Intrigued by the functionality of these two enzymes, a modular synthetic strategy (Fig. 2 and Supplementary Methods) was implemented to build NAM derivatives **2** and **3** (Fig. 1c).

To synthesize the necessary amounts of NAM derivatives, we optimized our laboratory's recently reported synthesis of 2-azido MDP^40^ (Fig. 2 and Supplementary Methods). First, we modified conditions^42^ to produce high gram quantities of the 2-azido intermediate **4** from commercially available glucosamine HCl (Fig. 2 and Supplementary Methods). Using our established method for installation of the lactic acid moiety, intermediate **9** was prepared. Global deprotection unmasked the azide to reveal the secondary amine at the 2-*N* position of the NAM core in gram quantities. Material could be stored at this intermediate. The terminal carboxylic acid of **9** is poised to hydrogen bond to the free amine at position 2 (Fig. 2, compound **10**), making coupling at the 2-*N* position challenging. Coupling conditions were developed to successfully synthesize NAM derivatives **2** and **3**. These compounds were characterized (Supplementary Figs 21, 23, 25 and 26) with an 18.5% overall yield from glucosamine HCl.

With the NAM derivatives in hand, we assessed the substrate specificity of the recycling enzymes, AmgK and MurU^41^. We found that these enzymes were able to convert **2** and **3** with bioorthogonal modifications such as an azide or alkyne at the 2-*N*-acetyl position, to yield the respective UDP carbohydrate products **2b** and **3b**, as confirmed by high-resolution liquid chromatography and mass spectrometry (HRLC/MS) (Supplementary Table 1). The ability to synthesize these unnatural NAM derivatives allowed for the demonstration that the recycling enzymes are promiscuous. From intermediates **2b** and **3b**, we were also able to demonstrate that the biosynthetic enzymes (MurC–MurF, Fig. 1b) have relaxed substrate specificity as confirmed by HRLC/MS (Supplementary Table 1). If these enzymes did not tolerate the unnatural modifications, the ability to modify the NAM carbohydrate would not be possible. These data motivated us to investigate if the metabolic incorporation of NAM into the PG of whole bacterial cells was achievable.

### NAM probes rescue bacteria from lethal dose of fosfomycin

**Figure 3:**
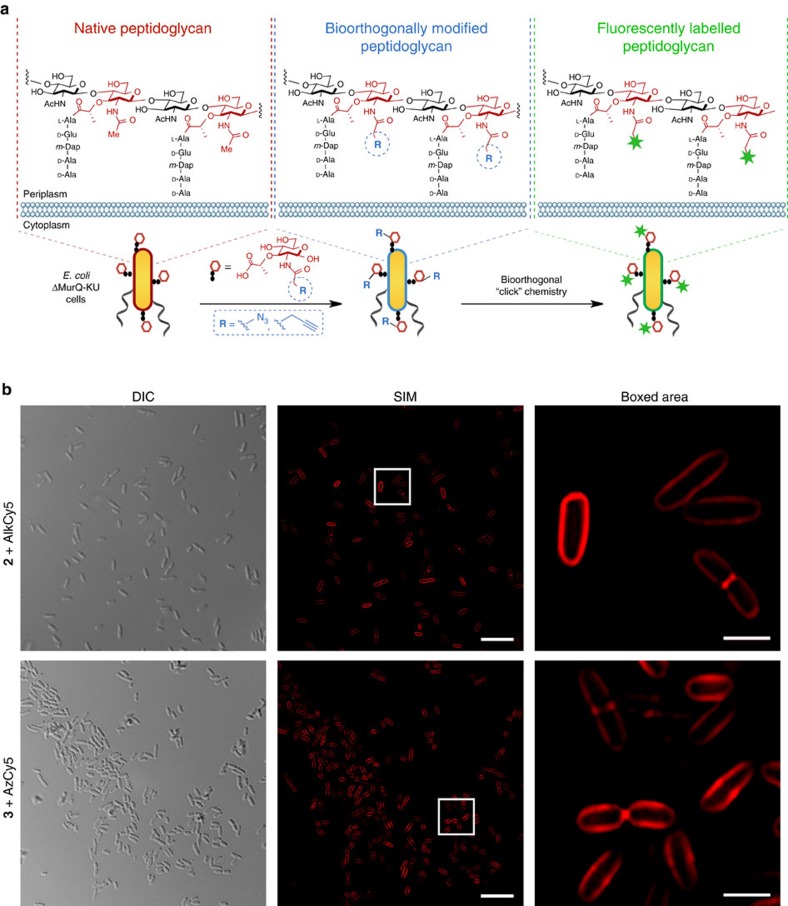
Fluorescent labelling of *E. coli* DMurQ-KU cells. (**a**) Overall PG remodelling strategy. To survive fosfomycin inhibition, cells utilize the promiscuity of the recycling and biosynthetic enzymes for incorporation of bioorthogonal NAM building blocks into PG. Click chemistry affords fluorescence modification onto the NAM backbone of bacterial PG. (**b**) DIC and individual 2D images from super-resolution SIM *Z*-stacks of cells treated with **2** or **3** and clicked with Cy5 (red) (scale bars, 10 μm for SIM and 2 μm in the boxed area). The 2D and 3D renderings are provided in Supplementary Movies 1 and 2, respectively. Images are representative of a minimum of five fields viewed per replicate with at least two technical replicates and the experiment was conducted in over ten biological replicates.

Knowing that the recycling and stage one biosynthetic pathways are promiscuous, a cell-based assay to exclusively label the PG polymer was implemented (Fig. 3a). First, it is known that in *E. coli* PG metabolism, NAM sugars can be imported by a phosphotransferase system (MurP) and yield *N*-acetylmuramic acid-6-phosphate (Fig. 1b)^43^. MurQ^44^, which is known as the only NAM-P etherase, can cleave off the D-lactic acid component of *N*-acetylmuramicacid-6-phosphate to produce NAG-6 phosphate (Fig. 1b). The fate of NAG-6 phosphate will be divided into two parts: (1) incorporation into new bacterial PG, and (2) degradation and utilization as the carbon and energy source^44^. To avoid potential metabolic incorporation of NAM probes through alternative catabolic pathways, an *E. coli* (MurQ) knockout strain was used (*E. coli* ΔMurQ)^41^. As *amgK* and *murU* are from *P. putida*, not native to *E. coli*, expression vector (pBBR-KU) was introduced to construct the *E. coli* ΔMurQ strains, which can express AmgK and MurU enzymes yielding *E. coli* ΔMurQ-KU (Methods).

Under the lethal concentration of fosfomycin, an antibiotic drug that can selectively inhibit the MurA enzyme^45^ (Fig. 1b), *E. coli* ΔMurQ-KU cells were unable to grow due to the inhibition of natural biosynthesis of UDP-MurNAc (Supplementary Fig. 1). Cell growth of *E. coli* ΔMurQ-KU can be restored in the presence of fosfomycin when cells were provided with an alternative way to synthesize UDP-MurNAc via the AmgK/MurU pathway and supplemented NAM sugars **1**–**3** (Fig. 1b,c and Supplementary Fig. 1). After 60–80 min, it was observed that cell growth in the presence of **2** slowed. As the cell growth is dependent of the availability of PG building blocks, we added an additional dose of NAM sugars (**1**–**2**). This treatment resulted in restoration of cell growth rate (Supplementary Fig. 1b). Importantly, this cell growth analysis demonstrated that the NAM building blocks are able to sustain growth in the presence of a lethal dose of fosfomycin. This implies that the cell can build PG with bioorthogonal NAM carbohydrates, which can be modified and subsequently visualized.

### Remodeling and labelling *E. coli* PG

To visualize PG, cells were grown in an optimized amount of either NAM derivatives **1**–**3**. After incubation (0.2% (w/v) of NAM derivative, see Methods and Supplementary Figs 1a and 2), a subsequent Copper (I)-catalysed azide-alkyne cycloaddition, also known as a ‘click' reaction^46^, was applied to introduce a fluorophore into the remodelled PG polymer (see Methods). Cells supplemented with either **2** or **3** were successfully labelled with the corresponding alkyne or azide fluorophore Cy5 (AlkCy5 or AzCy5) or Rhodamine 110 (Alk488 or Az488) and visualized through structured illumination microscopy (SIM)^47^ (Fig. 3b, Supplementary Fig. 3a and Supplementary Movies 1 and 2). Only background labelling was observed when *E. coli* ΔMurQ-KU cells were supplemented with **1** and treated under click conditions (Supplementary Fig. 4a). Cells without recycling enzymes AmgK and MurU (*E. coli* ΔMurQ-pBBR), and *E. coli* DH5α were not labelled with this method (Supplementary Fig. 4b,c, respectively). The data showed for the first time that carbohydrates could be directly visualized in the bacterial PG.

### Efficiency/selectivity studies of labelled *E. coli* PG

We measured the cell population labelling efficiency of the NAM method through fluorescence activated cell sorting (Supplementary Fig. 3b–e). The percentage of the *E. coli* ΔMurQ-KU cell population that fluoresce after incorporation with either **2** or **3** followed by Copper (I)-catalysed azide-alkyne cycloaddition with the appropriate conjugated dye was shown to be over 87% of the total number of cells when compared with the control **1** subjected to the same click conditions (Supplementary Fig. 3b–e and Supplementary Table 4).

To demonstrate that these probes are in fact embedded in the PG, we took advantage of the fosfomycin antibiotic selection of the *E. coli* ΔMurQ-KU cells (Fig. 1b). With the MurA pathway blocked, the only known NAM source for the bacteria to build their new PG must come from the substrates we supplement to the cells. As previously described, *E. coli* ΔMurQ-KU cell growth could be restored and sustained in the presence of a lethal dose of fosfomycin and the modified sugars **2** or **3** (Supplementary Fig. 1). Further, fluorescence microscopy in combination with fluorescence activated cell sorting demonstrates the percent of the *E. coli* ΔMurQ-KU population that fluoresced decreased when cells were grown in the absence of fosfomycin (Fig. 1b and Supplementary Fig. 5a,c). We observed a similar decrease in population labelling efficiency when labelled *E. coli* ΔMurQ-KU cells were treated with the cell wall digestive enzyme lysozyme, as this enzyme is known to specifically degrade PG and a decrease in labelling correlates with the decomposition of the polymer (Supplementary Fig. 5b,d).

To definitively show that the NAM probe was incorporated into PG, we desired MS evidence. Briefly, 488-labelled *E. coli* ΔMurQ-KU cells grown in the presence of **3** were treated with lysozyme (see Methods) and subjected to high-pressure liquid chromatography (HPLC) and high-resolution MS analyses (Supplementary Fig. 6a). We were able to identify and confirm potential mono-, di- and tetra-saccharide fragments of PG (compounds **13**–**17**) tagged with the 488 fluorophore (Supplementary Fig. 6b,c), demonstrating successful NAM incorporation and fluorescence modification on the *E. coli* ΔMurQ-KU PG. PG fragments **13**–**17** were not found in *E. coli* ΔMurQ-KU cells supplemented with **1** under the same conditions. Importantly, the natural, untagged lysozyme product **18** was found in both treatments with **1** and **3**, suggesting that PG was properly digested. The bioorthogonally tagged lysozyme product **19** was found solely in treatment with **3** (Supplementary Fig. 6b,c), implying that **3** was successfully incorporated into whole cell PG and then digested. The amount of modified NAMs that can be incorporated into bacterial PG is limited by the ability of fosfomycin to completely inhibit MurA (Fig. 1b). If we assume complete inhibition and complete conversion of the click reaction (Fig. 3a), then after one doubling time, 50% of the PG would be labelled. After two doubling times, 75% of the PG would be labelled. However, the percent of incorporation could be lower due to incomplete fosfomycin inhibition. Furthermore, the MS data indicate that the click labeling percentage is not 100% (Supplementary Fig. 6c). Together, the cell growth assays with and without fosfomycin (Supplementary Fig. 5a,c), lysozyme digestion assay (Supplementary Fig. 5b,d) and MS data (Supplementary Fig. 6b,c) support the conclusion that the probes are efficiently labelling these bacterial populations and specifically incorporating into the bacterial PG.

### Labelling of Gram-negative and Gram-positive bacterial PG

To demonstrate the utility of the method, the labelling strategy was implemented on additional Gram-negative bacterial strains. *P. putida* bacteria, which naturally have enzymes AmgK and MurU, and *E. coli* DH5α cells with inducible AmgK and MurU (*E. coli* KU) were successfully labelled (Supplementary Fig. 7a,b). As a control, *P. putida* and *E. coli* KU cells supplemented with **1** were not labelled (Supplementary Fig. 7a,b). To further extend this labelling strategy to Gram-positive bacterial strains, a *Bacillus subtilis* strain containing the *amgK* and *murU* gene cluster was constructed (*B. subtilis* 3A38-KU, Methods). After incubating with fosfomycin and compounds **2** or **3**, *B. subtilis* 3A38-KU cells were successfully labelled (Supplementary Fig. 7c). Cells incubated with **1** were not labelled (Supplementary Fig. 7c). These preliminary data indicate that both Gram-positive and Gram-negative bacteria can be labelled with the NAM method. Given the conservation of PG biosynthesis across all bacteria^2^, the labelling strategy has the potential to be applied to a broad range of bacterial strains, provided that the *amgK* and *murU* genes are present or can be introduced.

### Super-resolution microscopy reveals features of PG

**Figure 4:**
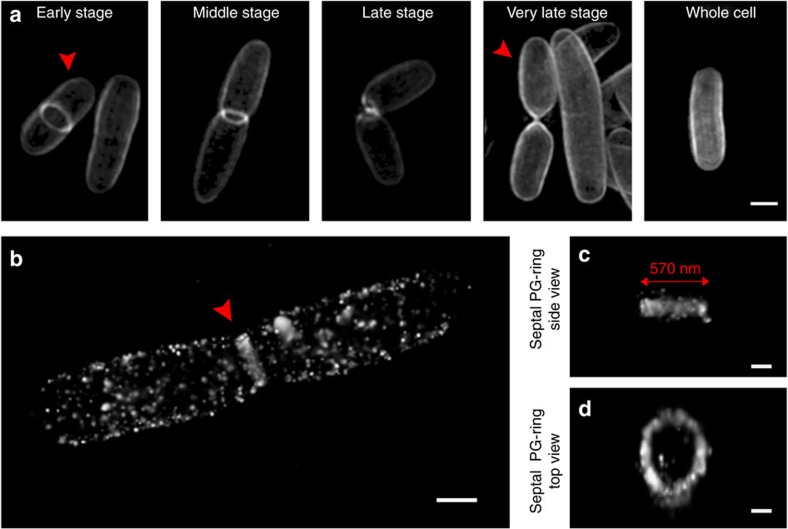
Visualization studies of the carbohydrate probes' incorporation into PG. (**a**) SIM images of 488 labelled *E. coli* ΔMurQ-KU cells at different cell division stages compiled from separate cell sample populations (scale bar, 1 μm). Images are representative of a minimum of five fields viewed per replicate with at least two technical replicates and the experiment was conducted in at least three biological replicates. (**b**) Three-dimensional STORM image of cells treated with **3** and labelled with AzCy5, whole-cell view (scale bar, 0.5 μm). Two-dimensional and 3D renderings are provided in Supplementary Movies 3 and 4, respectively. From cell shown in (**b**,**c**) side view of Septal PG-ring and (**d**) top view of Septal PG-ring. All images were generated and distances were calculated within the Zen programme as described in Methods (scale bars, 0.2 μm (**c**,**d**)). Images are representative of a minimum of two fields viewed per replicate with at least two technical replicates and the experiment was conducted in two biological replicates.

Although other methods have been reported to image PG through the introduction of fluorescent amino acids or utilizing fluorescent antibiotics, this method is the first to visualize the carbohydrate features of PG. The labelled bacteria were used to study the distribution and incorporation of the carbohydrate probe at the single molecule level. Cells pulsed with NAM derivatives for different time intervals were observed using SIM (100–140 nm lateral resolution). At 15 min pulse length with **3**, the accumulation of fluorescent signal was low (Supplementary Fig. 8a). The amount and distribution of this label reached its maximum after 30 min pulse length (Supplementary Fig. 8b). Therefore, the 45 min pulse length with unnatural sugars was used to achieve the best labelling effect. Among these cells, we were able to visualize the glycans at a variety of stages in the bacterial cell division cycle (Fig. 4a). Interestingly, a clearly defined ring of septal PG was labelled and visualized (Fig. 3b, Supplementary Fig. 3a and Supplementary Movie 2). The location of this ring structure is consistent with what has been proposed for the *Z*-ring, a circular polymer of FtsZ (a homologue of tubulin), which serves as the scaffold of divisome assembly^48^. Thus, NAM probes can be used to label PG synthesis by both the cell elongation and cell division machineries, and will facilitate future experiments investigating the coordination between PG synthesis and cell division. This equatorial ring appears to shrink with the elongation of the bacterial cells and disappears once the cell division is nearly complete. The high incorporation of probe proved useful for visualizing global structural features of cell division.

To study the fluorescent PG at a higher resolution than SIM, *E. coli* ΔMurQ-KU cells pulsed with **3** for 15 min were imaged using 3D stochastic optical reconstruction microscopy (3D-STORM, 10–20 nm resolution)^49^. The 15 min pulse length was chosen over the 45 min pulse length previously used for SIM, because the high incorporation saturated the finite details of cell wall architecture and shorter time points proved more useful for STORM. Moreover, a short time labelling period (15 min, less than one doubling time (Supplementary Fig. 1)) proves more useful in the study of nascent PG biosynthesis, as the cells would just start to load and incorporate the probes. Evenly distributed fluorescent signals on the cell wall were observed as opposed to the internal cellular environment (Fig. 4b and Supplementary Movies 3 and 4). We were also able to visualize concentrated signals localized in the septal PG-ring with a diameter, from a constricting cell, estimated to be 570 nm (Fig. 4c,d), consistent with what was reported for the *Z*-ring using labelled FtsZ^50^. From a selected region of the labelled cell, we were able to recognize a network of fluorescent carbohydrate strands that appeared to be interconnected (Supplementary Fig. 9a,b). The side view of this region showed that the fluorescent signal is on the surface of bacterial cells (Supplementary Fig. 9c). In some 3D-STORM images, ring-like structures (ranging from 90 to 180 nm) in the PG were observed (Supplementary Fig. 9d–f and Supplementary Movie 5), indicating that nascent bacterial PG synthesis could follow the pattern of either a circular arrangement of carbohydrate residues or a closed intersecting network of PG chains. This pattern could result from a specific carbohydrate loading process during PG biosynthesis or the presence of protein complexes/lipid rafts. However, the pattern could also be a consequence of incomplete NAM incorporation or click fluorescent labelling (Supplementary Fig. 6). It is possible that smaller carbohydrate rings exist, as we have reached the resolution limit of STORM^51^. Importantly, the STORM control in which *E. coli* ΔMurQ-KU cells pulsed with **1** for 15 min and treated with AzCy5 did not show any substantial signal (Supplementary Fig. 9g).

### Tracking of bacterial invasion and breakdown in macrophages

**Figure 5:**
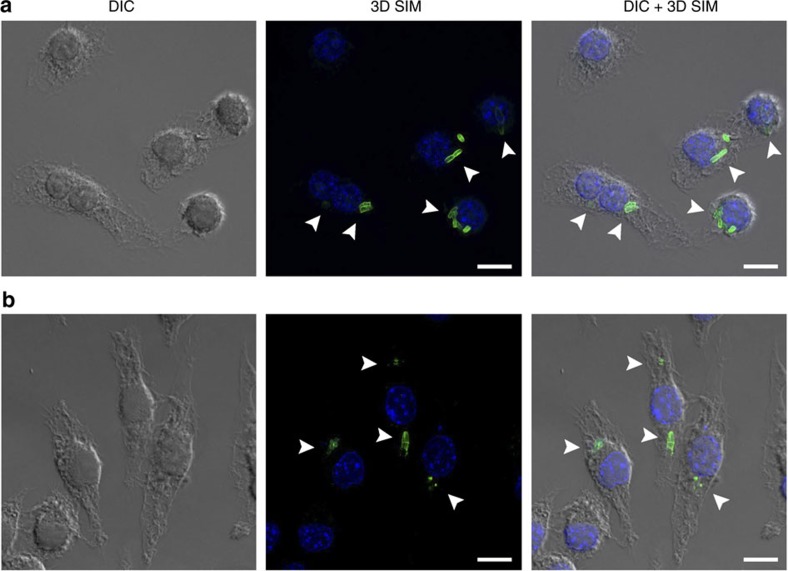
J774 mouse macrophage cells invaded by remodeled *E. coli*ΔMurQ-KU coli DMurQ-KU cells. (**a**) *E. coli* ΔMurQ-KU cells pre-treated with **3** for 45 min were then used to invade J774 cells for 1 h. Cells were fixed and Az488 was clicked into remodelled bacterial PG (green). Whole bacterial cells were visualized as shown with white arrows. Cellular DNA was labelled with 4,6-diamidino-2-phenylindole (blue) (scale bar, 10 μm). (**b**) Cells treated as in **a**, in which deformed bacterial cells with released fluorescent fragments were visualized as shown with white arrows. Fluorescent images are maximum intensity projections of *z*-stacks. Three-dimensional renderings are provided in Supplementary Movies 6 and 8. Images are representative of a minimum of three fields viewed per replicate with at least two technical replicates and invasion experiments were conducted in at least three biological replicates.

To determine whether this method would be useful in studying the interaction between NAM-based PG fragments and the innate immune system, NAM-labelled bacterial invasion of mammalian cells was studied. Bioorthogonally modified *E. coli* ΔMurQ-KU cells were incubated with J774 macrophage cells and were then subsequently labelled via click chemistry. SIM images confirm that the invasion of modified bacteria into the macrophage cytosol was successfully tracked, while unlabelled bacterial cells were not visualized (Fig. 5, Supplementary Fig 10a,b and Supplementary Movies 6 and 7). A population of macrophage cells contained deformed bacterial cells with released fluorescent fragments (Fig. 5b), which are confirmed to be inside the cells (Supplementary Fig. 10c and Supplementary Movie 8). As time increased, less whole bacterial cells were present inside the macrophage, while more fragments appeared (Supplementary Fig. 11a). We note that it is not clear whether these fragments are generated by macrophage digestion after invasion, or the normal break down of remodelled bacterial cells. When the remodelled bacterial cells were incubated in the DMEM medium in the absence of macrophage, a gradual decrease of bacterial cells was observed, implying that the cells could be degrading (Supplementary Fig. 11b), suggesting that remodelled cells cannot respond to some environmental stresses to the same degree as the wild-type cells. However, we note that the remodelled cells grow well in rich media (Supplementary Fig. 1). This labelling method allows selective tracking of the bacterial cell consumption and specific visualization of glycan units of PG upon macrophage infection, which can be used in future studies towards the identification and characterization of naturally occurring immunostimulatory NAM-containing PG fragments.

## Discussion

We have developed a robust method to study the NAM carbohydrate backbone in the *E. coli* PG and applied this method to examine fundamental questions in bacterial cell wall structure and bacterial invasion. To begin the investigation, we desired a method to synthesize large quantities of NAM derivatives with bioorthogonal modifications at the 2-*N* position. The simplistic structure of the PG NAM unit can be quite deceiving and derivatives are not widely available. With few previous reports of manipulation at the 2-*N* position of this carbohydrate^52^,^53^,^54^, we sought to modify an existing method developed by our lab to access large quantities of bioorthogonally tagged NAMs. As a highlight, the robust azido protecting group displayed at the 2-*N* position of the glycan can be unmasked late in the synthesis, to allow for optimized acylation to install bioorthogonal modifications at the 2-*N*-acetyl position. These derivatives that were previously unavailable until now can be accessed in the necessary quantities. Armed with desired PG intermediates **2** and **3**, a detailed analysis of the substrate specificity of the PG recycling and biosynthetic enzymes revealed that the conserved PG biosynthetic pathway is promiscuous, allowing for the installation of modified NAM residues. This *in vitro* analysis along with the reports of other successful PG labelling, allowed us to implement unnatural NAM building blocks as a tool to label whole cells.

We showed that PG from both Gram-positive and Gram-negative bacteria are able to be remodelled, labelled and visualized using this incorporation strategy. As *E. coli* and *B. subtilis* are the canonical model systems for Gram-negative and Gram-positive bacterium, the NAM labelling method should be applicable to a broad range of bacteria due to the conserved nature of the PG biosynthetic machinery. The data suggest that these labelled bacteria will also be useful in studying bacterial cell wall recycling in many bacterial strains. Interestingly, Mayer and colleagues^55^ recently reported that three Gram-positive bacterial strains, *Staphylococcus aureus*, *B. subtilis* and *Streptomyces coelicolor* that naturally have the *murP* and/or *murQ* genes, can use NAM in their cell wall recycling machinery. We propose that upon insertion of the *amgK* and *murU* genes into these bacterial strains, their PG would be remodelled and labelled with the NAM derivatives **2** and **3**, as demonstrated in our *B. subtilis* 3A38-KU work.

The SIM images (Fig. 4a and Supplementary Movie 2) presented with the NAM labelling method agree with the literature precedent for whole-cell PG labelling via fluorescently labelled amino acids^25^,^26^,^27^,^28^,^29^,^30^,^31^. Patterns of evenly distributed fluorescent signal along the cell body and probe concentration at the septal division site appear similar, but using SIM we are able to view the carbohydrates in higher resolution. However, helical wall patterns were not observed in the case of *B. subtilis* labelling as previously reported in fluorescently labelled ramoplanin and vancomycin PG^32^.

The super-resolution images of the NAM-labelled bacterial cells (Fig. 4 and Supplementary Movies 2–4) show that PG carbohydrate intermediates accumulate as a septal PG ring during cell division, providing preliminary evidence to the theory that PG biosynthesis and bacterial division are coordinated^56^. We suggest that these studies will be useful as an application to investigate the molecular details of the *Z*-ring. For example, co-localization of NAM probes and fluorescently labelled cell division enzymes may reveal the location and interaction between these enzymes and their associated NAM-containing carbohydrate intermediates. Although the *Z*-ring's structure has been characterized in detail at the protein level, the cell wall architecture, specifically at the carbohydrate level, is more challenging to address^57^.

A long-standing debate surrounds the orientation of the glycan chains in the bacterial cell wall^1^,^32^,^51^,^58^,^59^,^60^; models propose either a helical orientation or a linear glycan chain arrangement. For *E. coli*, the data presented in our study support the lack of a helical network at the carbohydrate level on the cell surface and proposes that the chains of glycans intersect to form a large circular network (Supplementary Fig. 9d–f and Supplementary Movie 5), with the helical structures derived from the peptide. Further experiments in which the PG is dual labelled (on both the carbohydrate and peptide) will be extremely valuable in understanding the way in which bacteria assemble this polymer.

The data also showed when remodelled *E. coli* was incubated with macrophages, not only were their engulfment by macrophages observed^21^,^22^,^30^, but also the breakdown of the bacterial PG was documented. These MDP-containing fragments could potentially be generated in two environments: (1) outside the cell during normal bacterial growth and division or (2) inside the cell during phagocytosis. Currently, MDP (Fig. 1a) is the standard bacterial PG fragment used by immunologists, as this synthetic fragment generates an immune response^5^,^12^,^13^,^14^. The ability to install probes on the conserved NAM carbohydrate will be useful in identifying the biologically relevant fragments of PG. Using this method, we have already potentially identified larger fragments of bacterial PG such as mono-, di- and tetra-saccharide compounds (**13**–**17**, Supplementary Fig. 6a–c). We note that these fragments were identified using small culture volumes (100 ml, Methods), implying that the method will be sensitive enough to identify fragments produced during macrophage infection.

This work began with the construction of a scalable synthesis of bioorthogonal NAM derivatives. We then developed a method to metabolically label the NAM glycan backbone of *E. coli*, *P. putida* and *B. subtilis* utilizing the promiscuity of cell wall recycling and biosynthetic machineries in whole cells. The results reveal fundamental architectural details of the glycan chains of the PG and further enable us to track the engulfment and breakdown of bacteria in macrophages.

Commensal and pathogenic bacteria are believed to produce PG fragments containing the core glycan NAM unit. Misrecognition of these molecular calling cards by the innate immune system can lead to the development of inflammatory bowel disease. Importantly, a long-standing debate around the biological relevance of the immunoactive synthetic fragment MDP remains unclear due to a lack of NAM-based probes. The development of these NAM probes and a method to incorporate them into the carbohydrate backbone of PG is a critical progression toward answering these questions. Selective labelling of the NAM residue at the core of PG has advanced the way the production and destruction of the polymer is visualized and studied in the presence or absence of immune cells. It is a true complement to the methods that previously exist to label PG and when used in concert the entire PG could be illuminated.

## Methods

### Note

For synthetic procedures, please see Supplementary Methods.

### Bacteria strains and plasmids

*E. coli* DH5α and BL21 (DE3) strains were from the laboratory stock. *E. coli murQ* knockout stain was obtained from the Coli Genetic Stock Center, CGSC (number 9928). *MurQ* gene was replaced with the kanamycin resistant gene^61^. *E. coli* strains were grown in liquid or solid Luria broth (LB) medium, supplemented with appropriate antibiotics: carbenicillin (100 μg ml^−1^), kanamycin (50 μg ml^−1^) or chloramphenicol (34 μg ml^−1^). *P. putida* strain KT2440 was purchased from ATCC (ATCC number 47054). *P. putida* was grown in LB medium or M9 minimal medium with NAM (0.2% w/v) as the carbon source. *B. subtilis* 3A38, a single point mutant of wild type *B. subtilis* strain 168, which has increased competence^62^, was obtained from Bacillus Genetic Stock Center. This cell strain was cultured in liquid or solid LB medium. Plasmid pGEX-6P-1, which contains a glutathione *S*-transferase (GST) affinity tag gene, was used in protein expression and purification. Plasmid pBBR1MCS was used for exogenous genes expression in *E. coli* strains. Plasmid pDG1662 was used to integrate the cloned gene cluster into *B. subtilis* 3A38 chromosome at the *amyE* locus, by a double-crossover recombination. Supplementary Information Table 2 shows a complete list of bacterial strains and plasmids used in this study. J774 macrophage cells were purchased from ATCC (catalogue number TIB-67), grown in DMEM supplemented with 10% heat-inactivated fetal bovine serum (Atlantic Biological) and 0.1% penicillin–streptomycin (Sigma-Aldrich) (unless otherwise noted) and routinely tested for mycoplasma (MycoAlert PLUS Mycoplasma Detection Kit, Lonza).

### Construction of expression plasmids and bacterial strains

Full-length *P. putida amgK* and *murU* were PCR amplified from *P. putida* genome DNA with 5′-BamHI and 3′-XhoI restriction sites (primer sets are PpAmgK-For/PpAmgK-Rev and PpMurU-For/PpMurU-Rev, respectively). PCR products of *amgK* and *murU* were subsequently ligated into pGEX-6P-1 vector to generate pGEX-PpAmgK plasmids and pGEX-PpMurU plasmids. *E. coli murC*, *murD*, *murE* and *murF* were PCR amplified from *E. coli* genome DNA. *E. coli murC* was inserted into pGEX-6P-1 vector with 5′-SalI and 3′-NotI restriction sites to generate pGEX-EcMurC plasmid (primer set is EcMurC-For/EcMurC-Rev). M*urD* and *murE* were inserted into pGEX-6P-1 with 5′-EcoRI and 3′-XhoI sites to get pGEX-EcMurD and pGEX-EcMurE plasmids (primer sets are EcMurD-For/EcMurD-Rev, EcMurE-For/EcMurE-Rev, respectively). *MurF* was inserted into the same vector using 5′-BamHI and 3′-XhoI sites to get pGEX-EcMurF vector (primers are EcMurF-For/EcMurF-Rev). Exogenous AmgK and MurU expression plasmid pBBR-KU was constructed from pBBR1MCS vector, using the PCR product of *amgK* and *murU* cluster, and the 5′-KpnI and 3′-HindIII restriction sites (primer set: pBBRKU-For/pBBRKU-Rev). The forward primer contained about 40 bp of the upstream region of *amgK* and *murU* cluster of *P. putida* genome DNA^41^. All inserted genes were confirmed by sequencing with plasmid sequencing primers (for pGEX-6 P-1 vector, using primer set 5GEX/3GEX, for pBBR1MCS vector, using primer set M13F (-21)/M13R). A complete list of primer sequences used in this study is shown in Supplementary Table 3.

*E. coli* ΔMurQ-KU and *E. coli* KU cell lines were constructed by transforming pBBR-KU vector into *E. coli* ΔMurQ and *E. coli* DH5α competent cells, respectively. Expression and function of AmgK and MurU enzymes were proved by a fosfomycin-susceptible agar diffusion assay. DNA of *amgK* and *murU* gene cluster with 5′-BamHI and 3′-HindIII restriction sites was cloned into pDG1662 plasmid to yield pDG-KU vector (primer set: pDGKU-For/pDGKU-Rev). pDG-KU vector was transformed into *B. subtilis* 3A38-competent cells as described^63^. Transformants after genomic recombination were selected on LB plate with chloramphenicol (5 μg ml^−1^) and marked as *B. subtilis* 3A38-KU strain. A complete list of primer sequences used in this study is shown in Supplementary Table 3.

### Protein expression and purification

Reconstructed pGEX expression plasmids were transformed into BL21(DE3) competent cells. After transformation, a 10 ml overnight BL21 cell culture was inoculated into 1 L fresh LB medium supplemented with 100 μg ml^−1^ carbenicillin antibiotics and incubated until OD_600nm_ reached 0.6. The expression of GST-tagged proteins was induced with 1 mM isopropyl-1-thio-β-D-galactoside at 18 °C for 20 h. Induced cells were harvested by centrifugation (4,000 *g*, 30 min) and resuspended in 20 ml GST lysis buffer (150 mM NaCl, 50 mM Tris, 2 mM dithiothreitol (DTT) pH 7.0, containing one protease inhibitor cocktail tablet from Roche). Cells were disrupted by two passes through a French Press at 10,000 psi and centrifuged at 27,000 *g* for 2 × 15 min to remove the cell debris. The supernatant was loaded onto a protein purification column with Glutathione Sepharose 4 Fastflow beads (GE Healthcare) and incubated at 4 °C for 1 h. The flow through was released and column was washed five times with 20 ml GST wash buffer (500 mM NaCl, 50 mM Tris, 1 mM DTT, 1 mM EDTA pH 7.0). After the washes, 10 ml GST elution buffer (150 mM NaCl, 50 mM Tris, 1 mM DTT and 1 mM EDTA pH 7.0) was added with an appropriate amount of PreScission Protease. The column was incubated at 4 °C overnight and purified protein was collected. For long-term storage at −20 °C, glycerol (20% final concentration) was added into the protein solution.

### Enzymatic reaction conditions

Activity and promiscuity of purified enzymes were studied in the enzymatic reactions. Products were analysed by HRLC/MS (Supplementary Information). Conditions for each enzymatic reaction are as follows:

AmgK: to 100 mM Tris buffer pH 7.9, 2.0 mM of one NAM derivative (**1** (Sigma-Aldrich) or **2** and **3**), 4.0 mM ATP and 1.0 mM MgCl_2_ was added 1.0 μg purified AmgK enzyme per 100 μl reaction sample. The reaction was incubated at room temperature for 2 h (ref. 41).

MurU: to 100 mM Tris buffer pH 7.9, 2.0 mM, **1a** to **3a** MurNAc-1P substrates, 4.0 mM UTP (Sigma-Aldrich) and 0.5 U of baker's yeast inorganic pyrophosphatase (Sigma-Aldrich) was added 1.0 μg purified MurU enzyme per 100 μl reaction sample. The reaction was incubated at 37 °C for 3 h (ref. 41).

MurC: to 100 mM Tris buffer pH 7.9, 2.0 mM of one UDP-MurNAc derivative (**1b**–**3b**), 15 mM (NH_4_)_2_SO_4_, 15 mM MgCl_2_, 2.5 mM 2-mercaptoethanol, 4.0 mM L-Ala, 4.0 mM ATP and 1.0 mM DTT was added 1.0 μg purified MurC enzyme per 100 μl reaction sample. The reaction was incubated at room temperature for 3 h (ref. 64).

MurD: to 100 mM Tris buffer pH 7.9, 2.0 mM of one UDP-MurNAc-L-Ala derivative (**1c**–**3c**, Supplementary Information), 4.0 mM D-Glu, 4.0 mM ATP and 2.0 mM MgCl_2_ was added 1.0 μg purified MurD enzyme per 100 μl reaction sample. The reaction was incubated at room temperature for 3 h (ref. 65).

MurE reaction: to 100 mM Tris buffer pH 7.9, 2.0 mM of one UDP-MurNAc-L-Ala-D-Glu derivative (**1d**–**3d**, Supplementary Information), 4.0 mM meso-DAP (Sigma-Aldrich), 4.0 mM ATP, 2.0 mM MgCl_2_ and 1.0 mM DTT was added 1.0 μg purified MurE enzyme per 100 μl reaction sample. The reaction was incubated at room temperature for 3 h (ref. 66).

MurF reaction: to 100 mM Tris buffer pH 7.9, 2.0 mM of one UDP-MurNAc-L-Ala-D-Glu-m-DAP derivative (**1e**–**3e**, Supplementary Information), 4.0 mM D-Ala-D-Ala (Sigma-Aldrich), 4.0 mM ATP and 2.0 mM MgCl_2_ was added 1.0 μg purified MurF enzyme per 100 μl reaction sample. The reaction was incubated at room temperature for 3 h (ref. 67).

### Bacterial cell growth curve study

Exponentially growing *E. coli* ΔMurQ-KU cells were diluted to OD_600nm_ 0.04. 1 ml of cell culture was incubated in 5 ml sterilized tubes. 0.2% (w/v) sugar substrates **1**–**3** and 200 μg ml^−1^ fosfomycin were added into experimental samples, and water was used as control. Three replicates samples were used for each study. Cells were incubated at 37 °C and OD_600nm_ was measured (Eppendorf 6136) and recorded every 20 min for 140 min in total. When additional dosage of NAM substrates **1**–**3** was added, they were added at 0.2% (w/v). Cell growth curves were formulated with GraphPad Prism 6 software.

### Bacterial cell wall remodelling and labelling

Overnight pre-cultured *E. coli* ΔMurQ-KU cells, *P. putida* cells or *B. subtilis* 3A38-KU cells were inoculated into fresh LB medium and were incubated until the OD_600nm_ was about 0.600. 1 ml of cells were collected by centrifugation at 6,000 *g* for 5 min. *E. coli* ΔMurQ-KU and *B. subtilis* 3A38-KU cells were resuspended in 200 μl LB medium and *P. putida* cells were resuspended in same amount of M9 minimal medium with 0.2% (w/v) Glucose. One of the NAM sugars **1**-**3** [0.2% (w/v)], and 200 μg ml^−1^ fosfomycin were added into both cell samples, whereas 1 mM isopropyl-1-thio-β-D-galactoside was only added to the *E. coli* cell samples. All cells were incubated at 37 °C for time ranging from 15 to 60 min, depending on the desired experiments. Cells were then collected (6,000 *g*, 5 min) and washed with 500 μl 1 × PBS buffer twice. Cells were resuspended in 200 μl 1:2 tert-butanol:water to prepare for the click reaction. To the bioorthogonally tagged bacterial cells was sequentially added 1 mM CuSO_4_ solution, 128 μM Tris[(1-benzyl-1H-1,2,3-triazol-4-yl)methyl]amine, 1.2 mM freshly prepared (+)− sodium (L) ascorbate (Sigma-Aldrich) and either 20 μM of Az488 or Alk488, or 2 μM AzCy5 or AlkCy5 (Sigma Aldrich, Supplementary Note). Cells were incubated at room temperature for 30 min (ref. 30). Cells were washed four times with 1 × PBS. The cells were resuspended in 100 μl 1 × PBS and prepared for imaging. All other *E. coli* strains were remodelled and labelled as *E. coli* ΔMurQ-KU cells.

### Structured illumination microscopy

Cover glasses (Zeiss; 22 mm × 22 mm) were treated with 0.1 mg ml^−1^ poly-L-lysine (Sigma-Aldrich) for 3 h at room temperature. Cover glasses were washed three times with deionized (DI) water and were air-dried at room temperature. Ten to twenty microlitres of labelled cells were loaded onto the centre of cover glass and incubated at room temperature for 30 min, to allow cell adherence. Cells were rinsed three times with 1 × PBS and fixed with 500 μl of 4% paraformaldehyde (stored under N_2_) at 4 °C for 30 min. Cover glasses with fixed cells were washed with 1 × PBS for three times and laid onto glass slides (Fisher Scientific) with 5 μl of ProLong Diamond Antifade Mountants (ThermoFisher) to cover the whole cover glass. SIM images were taken on a Zeiss Elyra PS.1 microscope with Plan-Apochromat × 63/1.4 Oil differential interference contrast (DIC) M27 objective. Excitation of Az488/Alk488 and AzCy5/AlkCy5 were achieved with 488 and 642 nm laser excitations, respectively. Camera exposure time was set to 100.0 ms and the raw data contained 5 rotations and 0.110 μm *z*-stack interval. SIM images were reconstructed from raw data with Carl Zeiss ZEN 2012. Processing and filtering settings were kept constant and image intensity was preserved with the raw image scale option in Zen 2012. Zen 2012 or Zen 2 software was used to generate two-dimensional (2D) and 3D images, respectively. Measurements were made in Zen 2012 using the line measurement tool from individual XY slices or YZ cross-sectional views.

### Three-dimensional STORM imaging

Cells were pulsed with **3** for 15 min and then labelled as described above with AzCy5 and fixed to poly-L-lysine-coated cover glass-bottom dishes (Matek, Inc). An oxygen scavenging buffer (10% glucose (w/v; Sigma-Aldrich 49163-100 ml), 62.5 μg ml^−1^ Catalase (Sigma-Aldrich C3115-50MG), 600 μg ml^−1^ Glucose Oxidase (Sigma-Aldrich G0543-50KU)) and imaging buffer containing 10 mM Cysteamine hydrochloride (MEA; Sigma-Aldrich M6500-25G) and 1 × PBS (pH 8.0)^68^,^69^,^70^ was added immediately before image acquisition. Three-dimensional STORM images were taken on the Zeiss Elyra PS.1 microscope with a Plan-Apochromat × 63/1.4 Oil DIC M27 objective using 642 nm laser excitation and a 655 nm long-pass filter. Camera exposure time was set to 18 ms with an electron-multiplying charge-coupled device gain of 300. Raw data were processed with Carl Zeiss ZEN 2 and molecules were filtered to 10 nm × 10 nm × 60 nm (XYZ) localization precision. Zen 2012 or Amira 6 software was used to generate 2D and 3D images.

### Flow cytometry

Bacteria labelled with either 488 or Cy5 were washed and resuspended in 100 μl 1 × PBS. Flow cytometry was performed on an Accuri C6 instrument, with DI water back flushing before and after each sample. Samples were vortexed for 10 s before each run. Cells (100,000) were analysed for each sample in triplicate and fluorescence intensities for R1-gated samples were measured. Histograms of fluorescence intensities (height) were generated and overlaid. Statistical data for mean FL-4H and mean FL-1H supplied in Supplementary Table 4 based on equal population number. The s.d. generated from technical triplicates.

### PG digestions and HPLC/MS analysis

Two 100 ml samples of *E. coli Δ*MurQ-KU cells were remodelled with **3** and then fluorescently labelled with Az-488 via click chemistry as described above. The same amount of cells was treated with **1** as control. All the samples were washed four times with 12 ml of 1 × PBS and finally stored as a dry pellet at −80 °C. Labelled and control cells were confirmed by microscopy as above. To digest the PG, the cell pellets were resuspended in 5 ml of digestion buffer (25 mM NaCl, 50 mM Tris, 2 mM EDTA pH 7.9), then freshly prepared lysozyme was added into each sample (1 mg ml^−1^ of the final concentration). Samples were incubated with agitation at 37 °C for 3 days, with the addition of 1 mg ml^−1^ freshly prepared lysozyme every ∼24 h. Digested samples were centrifuged for 5 s and the supernatant was filtered through Amicon Ultra 3K Filter Devices and lyophilized into a green/blue gel-like material, which was then dissolved in a minimal amount of DI water (20 μl). The samples were resolved on an Acquity UPLC BEH C18 column 2.1 × 50 mm (Waters) using a Dionex UHPLC coupled to a Q-Exactive Orbitrap (Thermo Fisher Scientific). The LC method was a 0.5 ml min^−1^ linear gradient starting from 0% A to 50% B in 4 min. Eluent A was 0.1% formic acid in water and Eluent B was 0.1% formic acid in acetonitrile. The absorbance of the eluting peaks was measured at 505 nm and further subjected high-resolution mass analysis on the Q-Exactive. All data were processed and analysed on a Thermo Xcalibur Qual Browser. All species not only showed the expected mass within ±5 p.p.m., but also the correct isotopic pattern.

### Macrophage invasion and immunostaining

The day before seeding the cells, sterile cover glasses (Fischer Scientific, catalogue number 12-545-80) were coated with 500 μl of 0.1 mg ml^−1^ poly-L-ornithine (Sigma-Aldrich) in 24-well plates overnight. One day before bacterial invasion, the poly-L-ornithine was removed and the cover glasses were washed with DMEM medium without antibiotics twice. J774 macrophages (provided by M. Parent, purchased ATCC catalogue number TIB-67) were seeded on these cover glasses in 24-well plates with DMEM medium (Methods) without antibiotics (penicillin–streptomycin) at a density of 1 × 10^5^ cells per well. For invasion with remodelled *E. coli* ΔMurQ-KU cells, bacteria were grown and remolded with modified NAM derivative **3** as described above. The bacteria (5 × 10^5^ cells) were added to the macrophage for different time lengths 20, 40, 60 and 80 min. After incubation, the medium was removed and fresh medium with gentamicin (1:1,000) was added to kill extracellular bacteria for 30 min at 37 °C. After 30 min, the medium was removed and cells were rinsed twice with 1 × PBS at room temperature. Cells were fixed with 4% formaldehyde in 1 × PBS for 10 min at room temperature. Cells were rinsed with 1 × PBS and then permeabilized with 1% Triton X-100 for 10 min at room temperature. Fixed cells were then rinsed in 1 × PBS containing 3% BSA and 0.1% Triton X-100 for 3 × 5 min. After rinsing cells in 1 × PBS, the click reaction was performed as described above in 1 × PBS with 0.01% BSA and 0.1% Triton X-100 for 30 min. After 30 min, the cells were rinsed in 1 × PBS and then washed in 1 × PBS containing 3% BSA and 0.1% Triton X-100 for 5 × 8 min. The cells were mounted on glass slides with 4,6-diamidino-2-phenylindole (Invitrogen) and ready for imaging.

### Data availability

The data that support the findings of this study are available from the corresponding author upon request.

## Additional information

**How to cite this article:** Liang, H. *et al*. Metabolic labelling of the carbohydrate core in bacterial peptidoglycan and its applications. *Nat. Commun.* **8**, 15015 doi: 10.1038/ncomms15015 (2017).

**Publisher's note:** Springer Nature remains neutral with regard to jurisdictional claims in published maps and institutional affiliations.

## Supplementary Material

Supplementary Information Supplementary Figures, Supplementary Tables, Supplementary Note, Supplementary Methods and Supplementary References

Supplementary Movie 1 Z-stack of SIM images show the labeling of bacterial peptidoglycan. 2-D z-stack SIM images (seen in Fig. 3b) are generated from Carl Zeiss ZEN 2012 (Methods). E. coli ΔMurQ-KU cells were treated with 2 and labeled with AlkCy5 (red) via click chemistry.

Supplementary Movie 2 Video generated from 3-D SIM showing the rotation of 488-labeled bacterial cells. 3-D renderings are generated from SIM z-stacks with Carl Zeiss ZEN 2012 (seen in Supplementary Fig. 3a, Methods). E. coli ΔMurQ-KU cells were treated with 2 and labeled with Alk488 (green) via click chemistry

Supplementary Movie 3 Z-stack of STORM images show the labeling of bacterial peptidoglycan. 2-D z-stack STORM images (seen in Fig. 4b and Supplementary Fig. 9a) are generated from Carl Zeiss ZEN 2012 (Methods). E. coli ΔMurQ-KU cells were treated with 3 for 15 min and labeled with AzCy5 via click chemistry.

Supplementary Movie 4 Video generated from 3-D STORM showing the rotation of labelled bacterial cells. 3-D renderings are generated from STORM z-stacks with Carl Zeiss ZEN 2 (seen in Fig. 4b and Supplementary Fig. 9a, Methods). E. coli ΔMurQ-KU cells were treated with 3 for 15 min and labelled with AzCy5 via click chemistry. Renderings are rotated 360 degrees around the y-axis.

Supplementary Movie 5 Video generated from 3-D STORM showing ring-like structures of peptidoglycan. 3-D renderings are generated from STORM z-stacks with Carl Zeiss ZEN 2 and video is made with Amira 6 software. E. coli ΔMurQ-KU cells were treated with 3 for 15 min and labeled with AzCy5 via click chemistry. Ring-like structures are highlighted with red circles.

Supplementary Movie 6 3-D projections showing J774 cells with fluorescent labeled bacterial cells inside. 3-D renderings are generated from SIM z-stacks with Carl Zeiss ZEN 2012 (seen in Fig. 5a, Methods). J774 cells are invaded for 1 h with E. coli ΔMurQ-KU cells that were pre-treated with 3 for 45 min. Cells were fixed and remodeled bacterial peptidoglycan was labeled with Az488 via click chemistry (green). Cellular DNA was stained with DAPI (blue). Whole bacterial cells were visualized inside the J774 cells. Renderings are rotated 360 degrees around the y-axis.

Supplementary Movie 7 3-D projections showing the engulfment of remodeled bacterial cell into J774 cell. 3-D renderings are generated from SIM z-stacks with Carl Zeiss ZEN 2012 (seen in Supplementary Fig. 10b, Methods). Cells were treated as described in Supplementary Movie 6. One dividing bacterial cell was visualized in the process of engulfment into the J774 cells. Renderings are rotated 360 degrees around the y-axis.

Supplementary Movie 8 3-D projections showing J774 cells with deformed bacterial cells and fluorescent fragments inside. 3-D renderings are generated from SIM z-stacks with Carl Zeiss ZEN 2012 (seen in Fig. 5b, Methods). Cells were treated as described in Supplementary Movie 6. Deformed bacterial cells with released fluorescent fragments were visualized inside the J774 cells. Renderings are rotated 360 degrees around the y-axis.
