# Supplementary material for: Metabolic labelling of the carbohydrate core in bacterial peptidoglycan and its applications
Source: Nat Commun. 2017 Apr 20;8:15015. doi: 10.1038/ncomms15015 (PMC5411481; doi:10.1038/ncomms15015)
Supplement: Supplementary Information — Supplementary Figures, Supplementary Tables, Supplementary Note, Supplementary Methods and Supplementary References [file ncomms15015-s1.pdf]

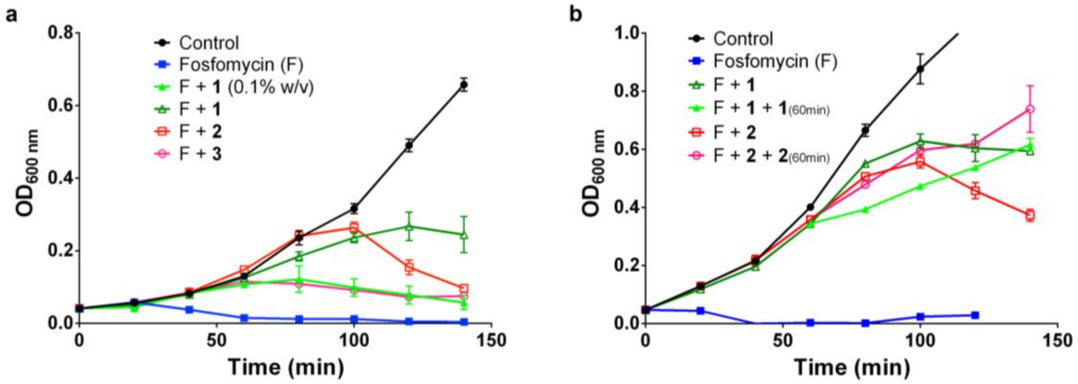

Supplementary Figure 1 | Growth curve analysis of *E. coli* ΔMurQ KU cells treated with fosfomycin and supplemented with NAM derivatives. a, *E. coli* ΔMurQ KU cell growth study in the absence (control) and presence of fosfomycin, supplemented with 0.2% w/v of **1**, **2**, or **3**. **b,** Growth curve analysis as described in (**a**), with a second dose (0.2% w/v) of NAM **1** or **2** added to *E. coli* ΔMurQ KU cells at 60 minutes. Experiments shown in (**a**) and (**b**) were conducted at least 3 times each. Standard deviations (s.d.) in both **a** and **b** are from 3 biological replicates.

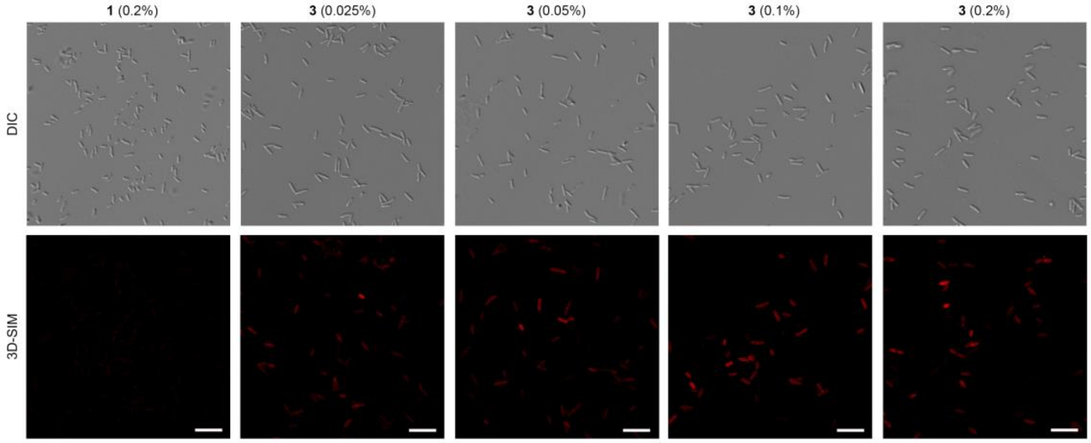

Supplementary Figure 2 | Fluorescence imaging of *E. coli* ΔMurQ KU cells remodeled with different concentrations of 3 and labeled with Cy5. Differential interference contrast (DIC) and two dimensional Z-stack SIM images of cells treated with **1** or varying concentrations of **3** then clicked with Cy5 (red) (scale bars, 10 μm for SIM). Images are representative of a minimum of 5 fields viewed per replicate with at least 2 technical replicates and the experiment was conducted in at least 3 biological replicates.

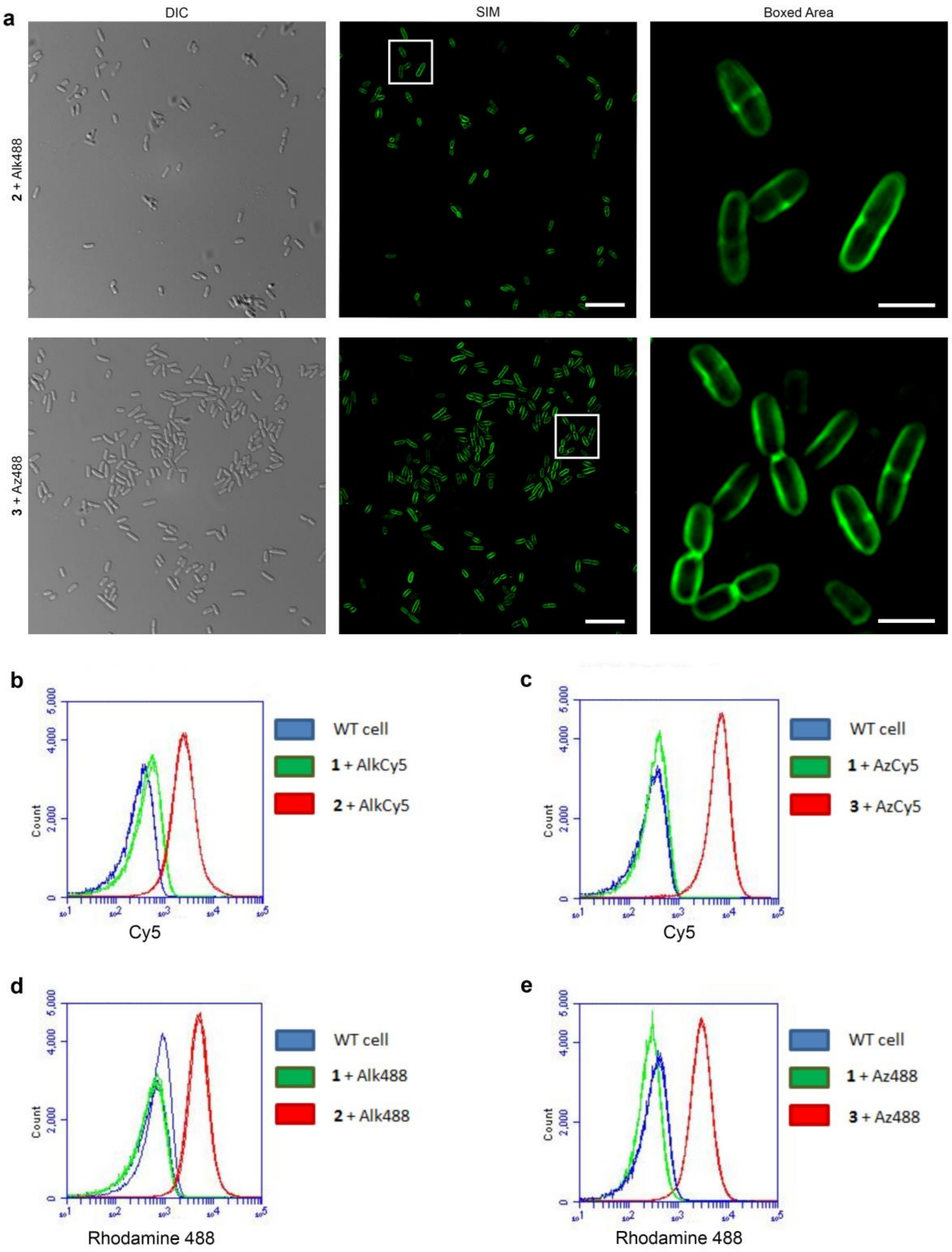

Supplementary Figure 3 | Fluorescence imaging of *E. coli* ΔMurQ KU cells labeled with 488 and FACS studies. **a**, DIC and two dimensional Z-stack SIM images of cells treated with **2** or **3** then clicked with 488 (green) (scale bars, 10 μm for SIM, 2 μm for

boxed area). Images are representative of a minimum of 5 fields viewed per replicate with at least 2 technical replicates and the experiment was conducted in at least 3 biological replicates. **b-e**, Cell population labeling efficiency measured through FACS with *E. coli* ΔMurQ KU. Cells were labeled with either AlkCy5 (3 biological replicates, 3 technical replicates), AzCy5 (2 biological replicates, 3 technical replicates), Alk488 (2 biological replicates and 3 technical replicates) or Az488 (2 biological replicates and 3 technical replicates). WT cells refer to untreated *E. coli* ΔMurQ KU. Overlays are of 3 technical replicates of one representative biological sample.

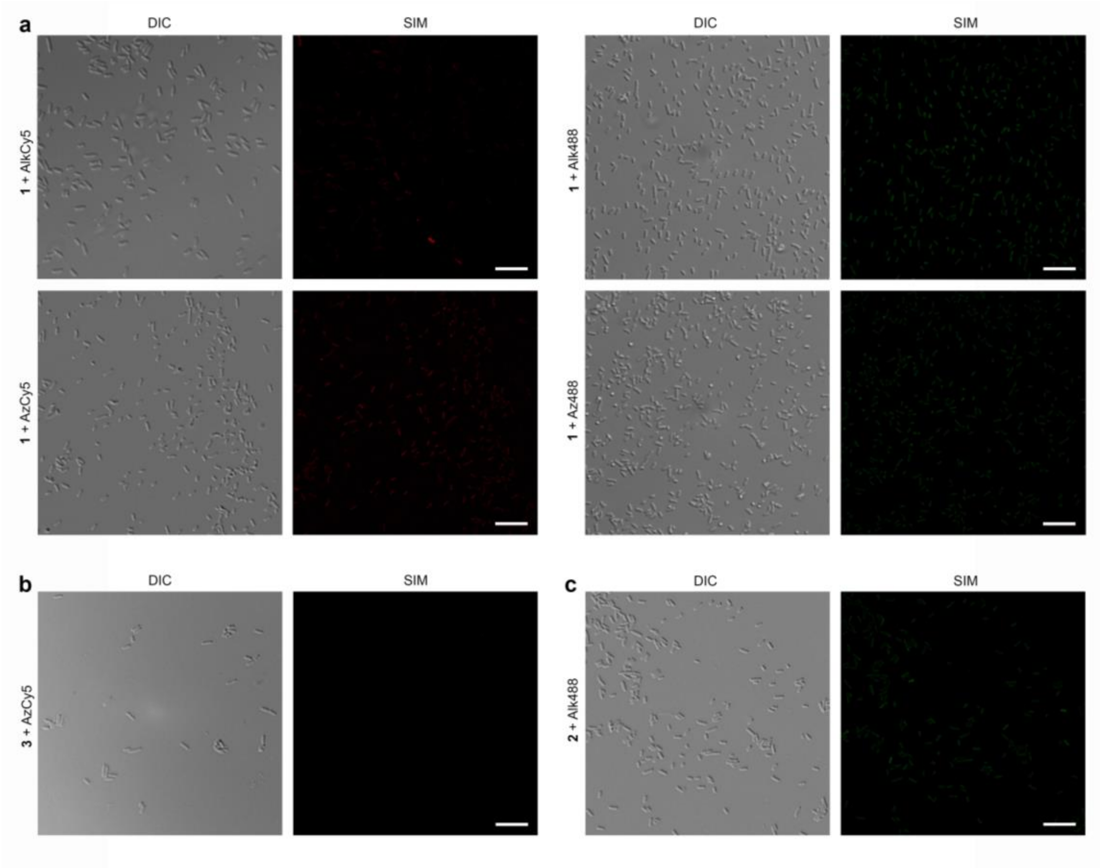

Supplementary Figure 4 | Fluorescence imaging of *E. coli* ΔMurQ KU controls, *E. coli* ΔMurQ-pBBR, and *E. coli* DH5α cells. a, *E. coli* ΔMurQ KU cells were supplemented with **1** and treated under click conditions (scale bars, 10 μm). Images are representative of a minimum of 3 fields viewed per replicate with at least 2 technical replicates and the experiment was conducted in at least 3 biological replicates. **b**, *E. coli* ΔMurQ pBBR supplemented with compound **3** treated with AzCy5. Images are representative of a minimum of 5 fields viewed per replicate with at least 2 technical replicates and the experiment was conducted in at least 2 biological replicates. **c,** *E. coli* DH5α cells supplemented with compound **2** and treated with Alk488. Only background labeling was observed in all samples (scale bars, 10 μm). Images are representative of a

minimum of 5 fields viewed per replicate with at least 2 technical replicates and the experiment was conducted in at least 2 biological replicates.

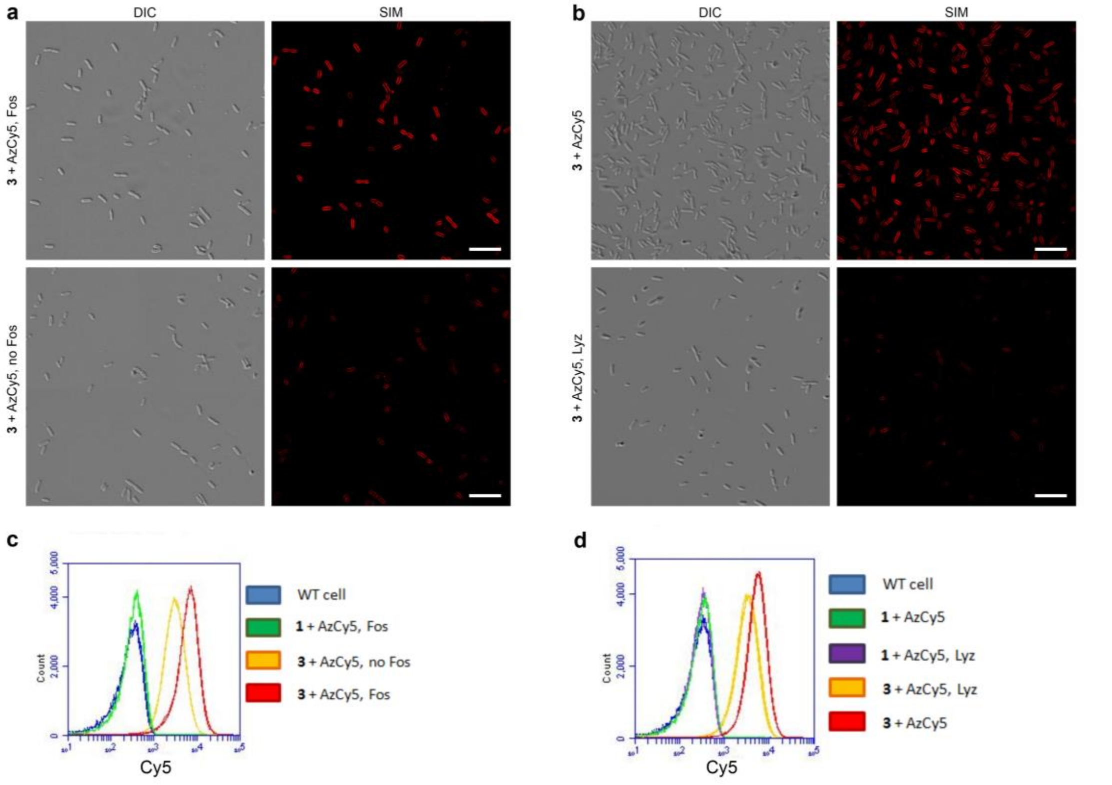

Supplementary Figure 5 | Peptidoglycan incorporation studies with fosfomycin and lysozyme treatment. a, *E. coli* ΔMurQ-KU (WT cell) cells grown in the presence or absence of fosfomcin (Fos), NAM derivative **3**, IPTG, and clicked with AzCy5 (scale bars, 10 μm). Images are representative of a minimum of 5 fields viewed per replicate with at least 2 technical replicates and the experiment was conducted in at least 3 biological replicates. **b,** *E. coli* ΔMurQ-KU cells supplemented with NAM derivative **3**, IPTG, grown treated or untreated with Lysozyme (Lyz) for 40 min, and clicked with AzCy5 (scale bars, 10 μm). Images are representative of a minimum of 5 fields viewed per replicate with at least 2 technical replicates and the experiment was conducted in at least 2 biological replicates **c,** Cell population labeling efficiency measured through

FACS with *E. coli* ΔMurQ KU cells in the presence or absence of fosfomycin (Fos). Cells were incubated with either **1** or **3**, with or without Fos, and then labeled with AzCy5 (3 biological replicates for each condition with 3 technical replicates were conducted). WT cell refers to untreated *E. coli* ΔMurQ KU. Overlays are of 3 technical replicates of one representative biological sample. **d,** Cell population labeling efficiency measured through FACS with *E. coli* ΔMurQ KU cells in the presence or absence of lysozyme (Lyz). Cells were incubated with either **1** or **3**, treated with or without Lyz, then labeled with AzCy5 (2 biological replicates for each condition with 3 technical replicates were conducted). WT cell refers to untreated *E. coli* ΔMurQ KU. Overlays are of 3 technical replicates of one representative biological sample.

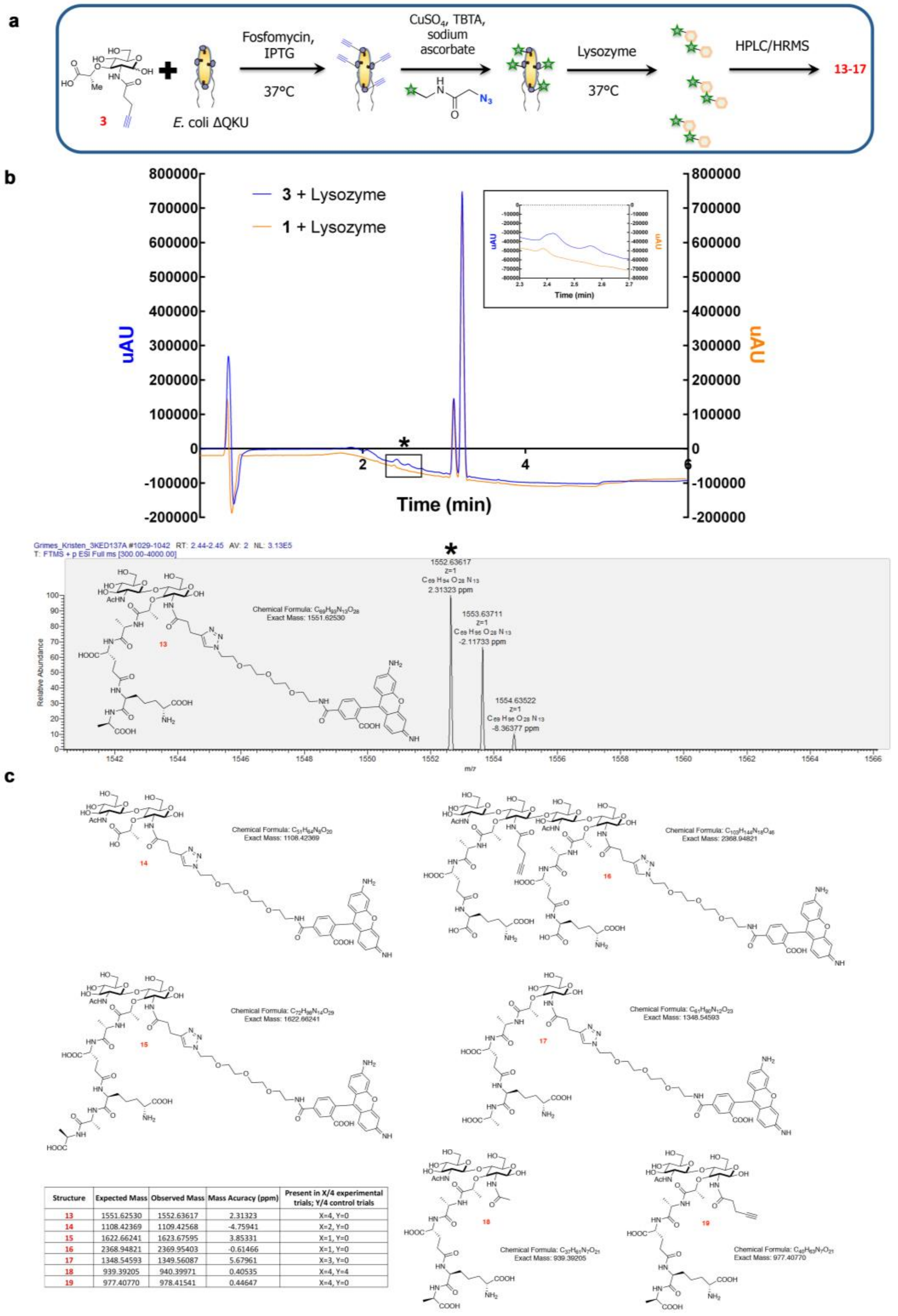

Structure	Expected Mass	Observed Mass	Mass Acuracy (ppm)	Present in X/4 experimental trials; Y/4 control trials
13	1551.62530	1552.63617	2.31323	X=4, Y=0
14	1108.42369	1109.42568	-4.75941	X=2, Y=0
15	1622.66241	1623.67595	3.85331	X=1, Y=0
16	2368.94821	2369.95403	-0.61466	X=1, Y=0
17	1348.54593	1349.56087	5.67961	X=3, Y=0
18	939.39205	940.39971	0.40535	X=4, Y=4
19	977.40770	978.41541	0.44647	X=4, Y=0

Supplementary Figure 6 | Lysozyme digestion *E. coli* ΔMurQ KU cells labeled with 488; HPLC/MS fragment identification. a, Overall experimental strategy to identify fluorescent PG fragments in *E. coli* ΔMurQ KU cells labeled with 488. **b,** UV absorbance

HPLC trace (505 nm) of *E. coli* ΔMurQ KU cells remodeled with **1** (control trials) or **3** (experimental trials), clicked with Az 488, then treated with lysozyme [**1** + Lyz (orange trace), **3** + Lyz (blue trace), respectively] (top). Boxed area is zoomed in at retention time 2.3-2.7 min. HRMS spectrum for the blue trace (**3** + Lyz) in the boxed retention time in (**b**) corresponding to compound **4** (bottom). **c,** Chemical structures, expected masses, and observed masses with mass accuracy (ppm) of PG fragments 1) **13-17** present in *E. coli* ΔMurQ KU cells treated as in **a**, which are not present in *E. coli* ΔMurQ KU cells treated with control compound **1**; 2) natural PG lysozyme product **18** present in both control and experimental samples and 3) alkyne-PG lysozyme product **19** present only in experimental samples. In addition, the number of times each compound was found per control and experimental paired trial are indicated. For example, **13** was present in four (X) out of four experimental trials and not found zero (Y) of the four corresponding control trials. The experiment was conducted in 2 technical replicates of 2 biological replicates.

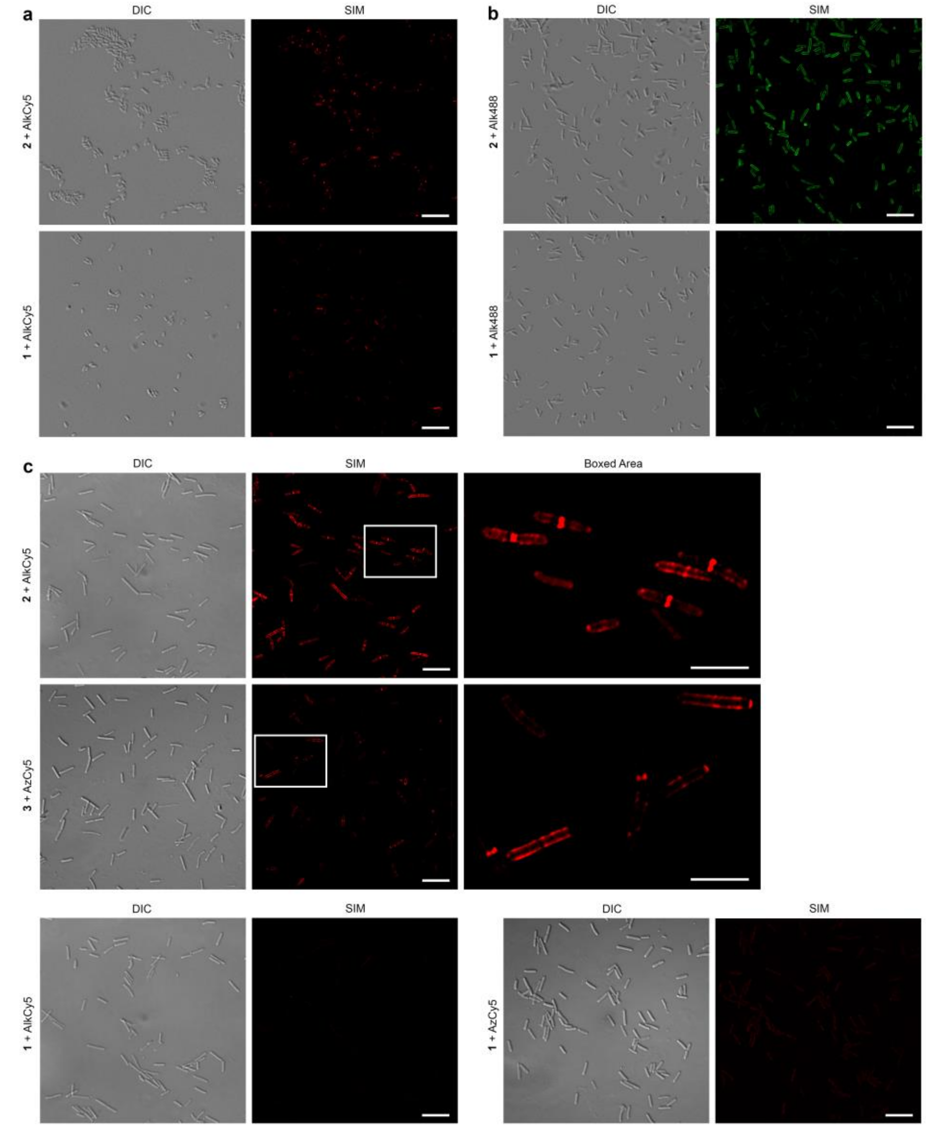

Supplementary Figure 7 | Fluorescence imaging of labeled *P. putida, E. coli* KU, and *B. subtilis* 3A38 KU-cells. a, *P. putida*, **b,** *E. coli* KU, and **c,** *B. subtilis* 3A38-KU cells were supplemented with **1, 2** or **3**, separately. Cells were subjected to click chemistry with AlkCy5 (red) or Alk488 (green). Cells treated with compound **2** or **3** were labeled and visualized with SIM while the control cells treated with compound **1** showed only

background fluorescence (scale bars, 10 μm for SIM, 5 μm for Boxed Area). Images are representative of a minimum of 3 fields viewed per replicate with at least 2 technical replicates and the experiment was conducted in at least 2 biological replicates.

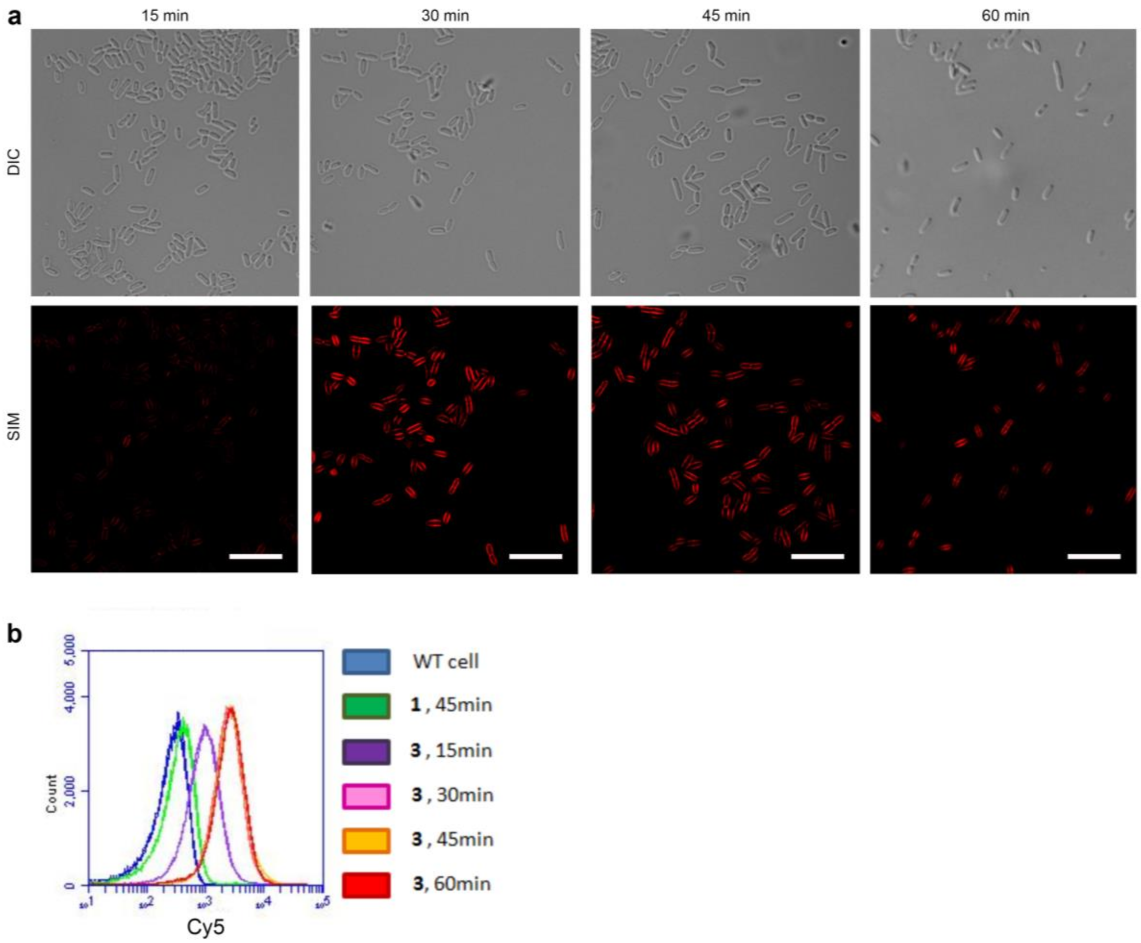

Supplementary Figure 8 | Time course labeling study of *E. coli* ΔMurQ-KU cells. a, *E. coli* ΔMurQ-KU cells pulsed with **3** for 15, 30, 45, and 60 min followed by click chemistry with AzCy5 (scale bars, 10 μm). Images are representative of a minimum of 3 fields viewed per replicate with at least 2 technical replicates and the experiment was conducted in 2 biological replicates. **b,** Cell population labeling measurements of *E. coli* ΔMurQ KU cells treated as in (**a**) measured through FACS. WT cell refers to untreated *E. coli* ΔMurQ KU cells. Experiment was performed in 2 biological replicates with 3 technical replicates. Overlays are of 3 technical replicates of one representative biological sample.

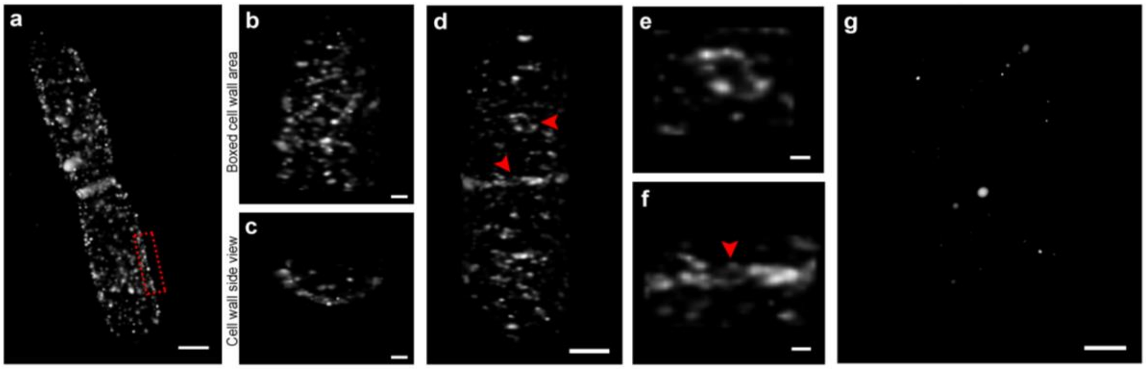

Supplementary Figure 9 | 3-D STORM image of *E. coli* ΔMurQ-KU and view of circular rings and negative control. a, 3-D STORM image whole cell view, same as Fig. 4b (scale bar, 0.5 μm). Two and three-dimensional renderings are provided in Supplementary Movies 3 and 4, respectively. **b,** Boxed cell wall area with linear intersecting fluorescent patterns and **c**, side view of the cell wall edge. All images were generated and distances were calculated within the Zen program as described in methods (scale bars in **b-c**, 0.2 μm). **d,** Front view of an *E. coli* ΔMurQ-KU cell treated with **3** and AzCy5 showing (scale bar, 0.5 μm) **e,** large and **f,** small fluorescent ring structures (scale bars, 0.1 μm). **g,** Top view of a group of *E. coli* ΔMurQ-KU cells treated with **1** and labeled with AzCy5 (scale bars, 1 μm). Images are representative of a minimum of 3 fields viewed per replicate with at least 2 technical replicates and the experiment was conducted in 2 biological replicates.

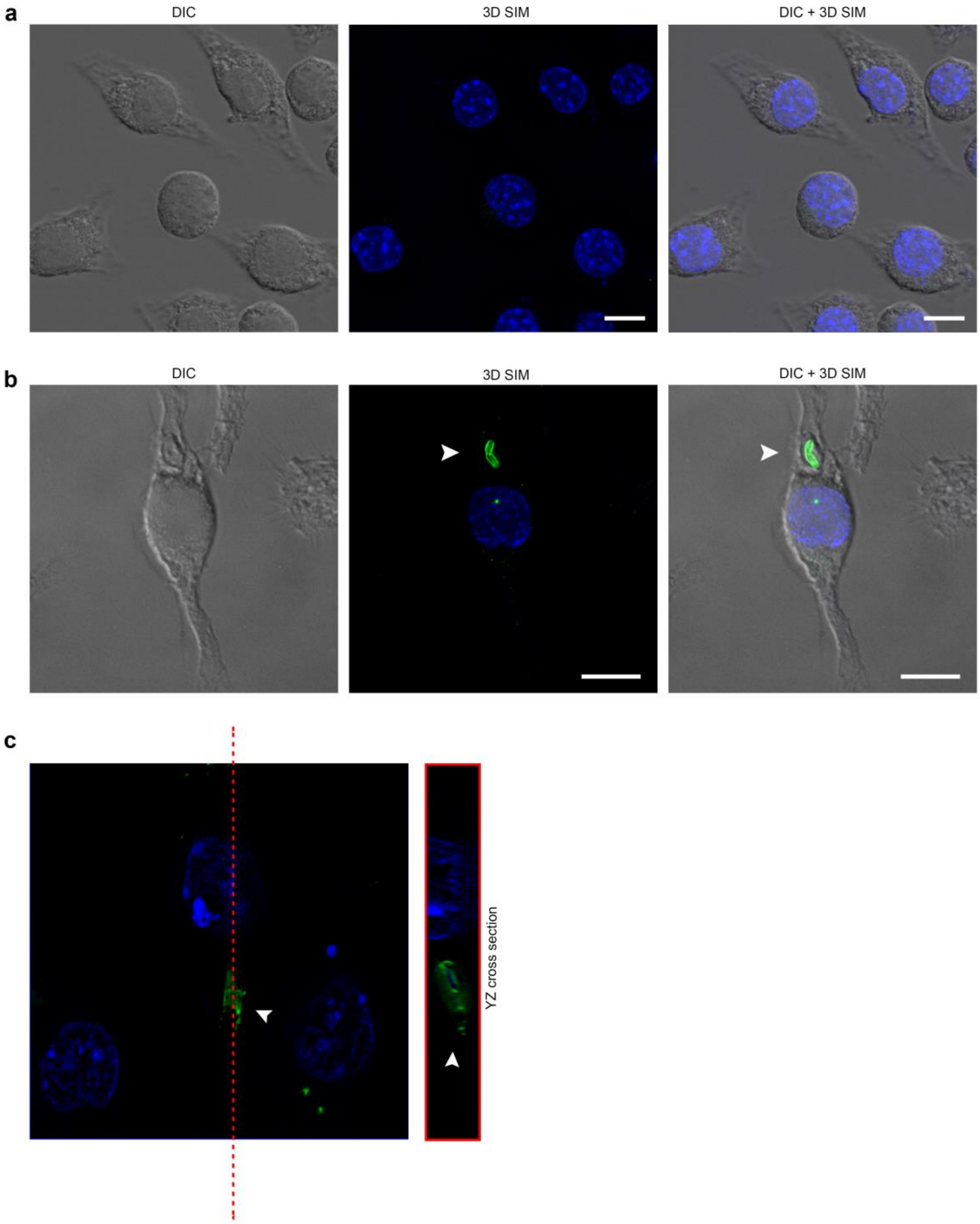

Supplementary Figure 10 | J774 macrophage invasion and cross section view. a, *E. coli* ΔMurQ-KU cells pre-treated with **1** for 45 min were then used to invade J774 cells for 1 h. Cells were fixed and treated with Az488 (green) and click conditions. SIM microscopy shows J774 macrophages without any 488 fluorescence. Cellular DNA was

labeled with DAPI (blue) (scale bars, 10 μm). Images are representative of a minimum of 3 fields viewed per replicate with at least 2 technical replicates and the experiment was conducted in at least 3 biological replicates. **b,** All cells treated the same as described in Fig. 5a and imaged with SIM. A dividing bacterial cell was visualized as shown with white arrows (scale bars, 10 μm). Images are representative of a minimum of 3 fields viewed per replicate with at least 2 technical replicates and the experiment was conducted in 2 biological replicates. Three-dimensional rendering of **b** is provided in Supplementary Movie 7. **c,** Cross section view of J774 macrophages invaded with remodeled and labeled *E. coli* ΔMurQ-KU cells. This image is taken from the same cell shown in Fig. 5b and reveals the fragments are inside the macrophage cell. Cellular DNA was labeled with DAPI (blue).

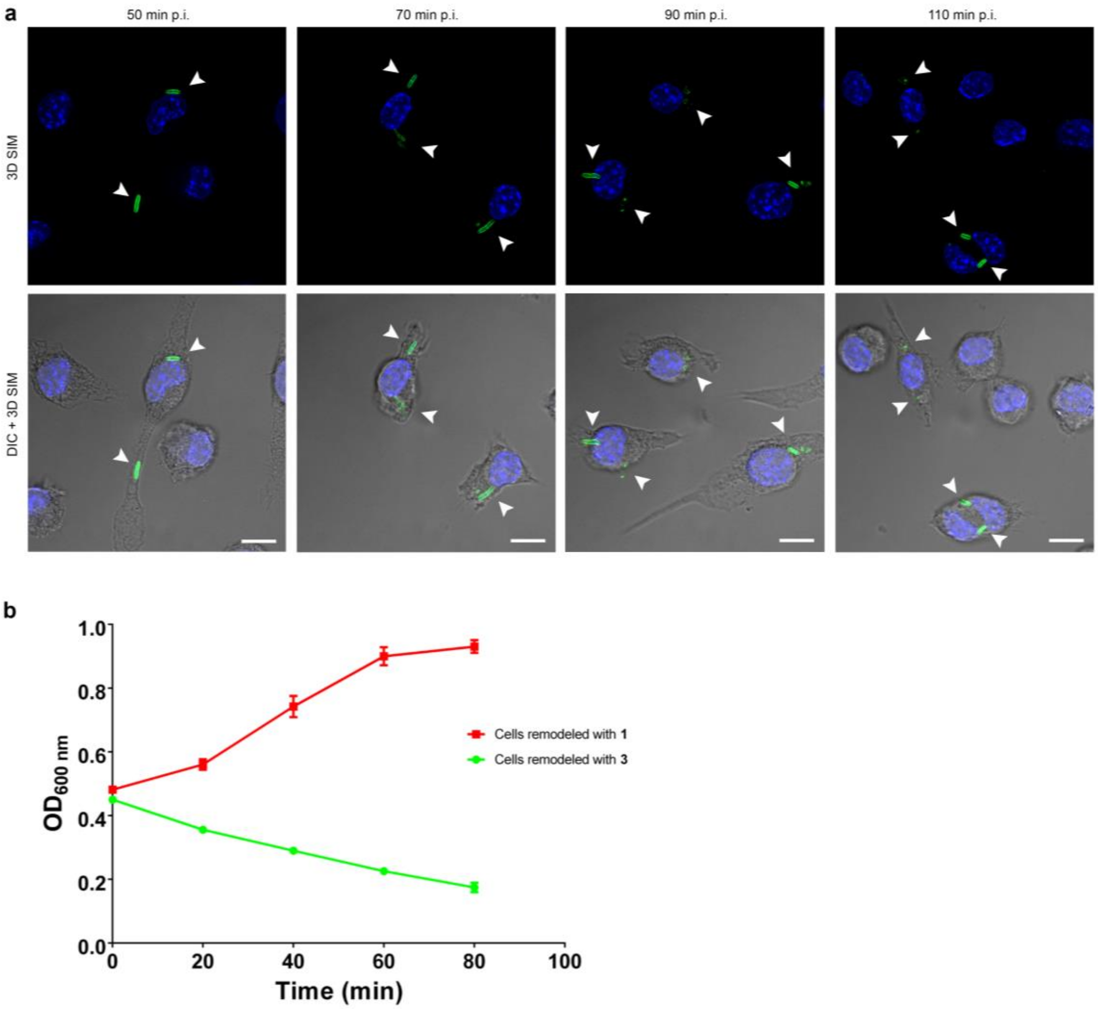

Supplementary Figure 11 | Time course study of J774 macrophage invasion. a, *E. coli* ΔMurQ KU cells pre-treated with **3** for 45 min were then used to infect J774 cells for 20, 40, 60 or 80 minutes. Macrophage cells were treated with gentamycin for an additional 30 min. then collected at total time points 50, 70, 90 and 110 min post invasion (p.i.). Cells were fixed and Az488 was clicked into remodeled bacterial peptidoglycan (green). DAPI (blue) was used for cellular DNA staining. Three-dimensional SIM imaging (top) and DIC + 3-D SIM overlay (bottom) are shown. Arrowheads point to corresponding labeled bacterial cells or fragments. Images shown are one representative field for each time point (scale bars, 10 μm). Fluorescent images are maximum intensity

projections of *z*-stacks. Images are representative of a minimum of 3 fields viewed per replicate with at least 2 technical replicates and the experiment was conducted in at least 3 biological replicates. **b,** *E. coli* ΔMurQKU cell condition study as described in **a** in DMEM medium (Methods) without antibiotics (pen-strep), macrophage or gentamycin treatment. Cells were not provided additional PG building blocks or antibiotics during this DMEM treatment. Experiments were conducted at least 2 times. Standard deviations (s.d.) in **b** are from 3 biological replicates.

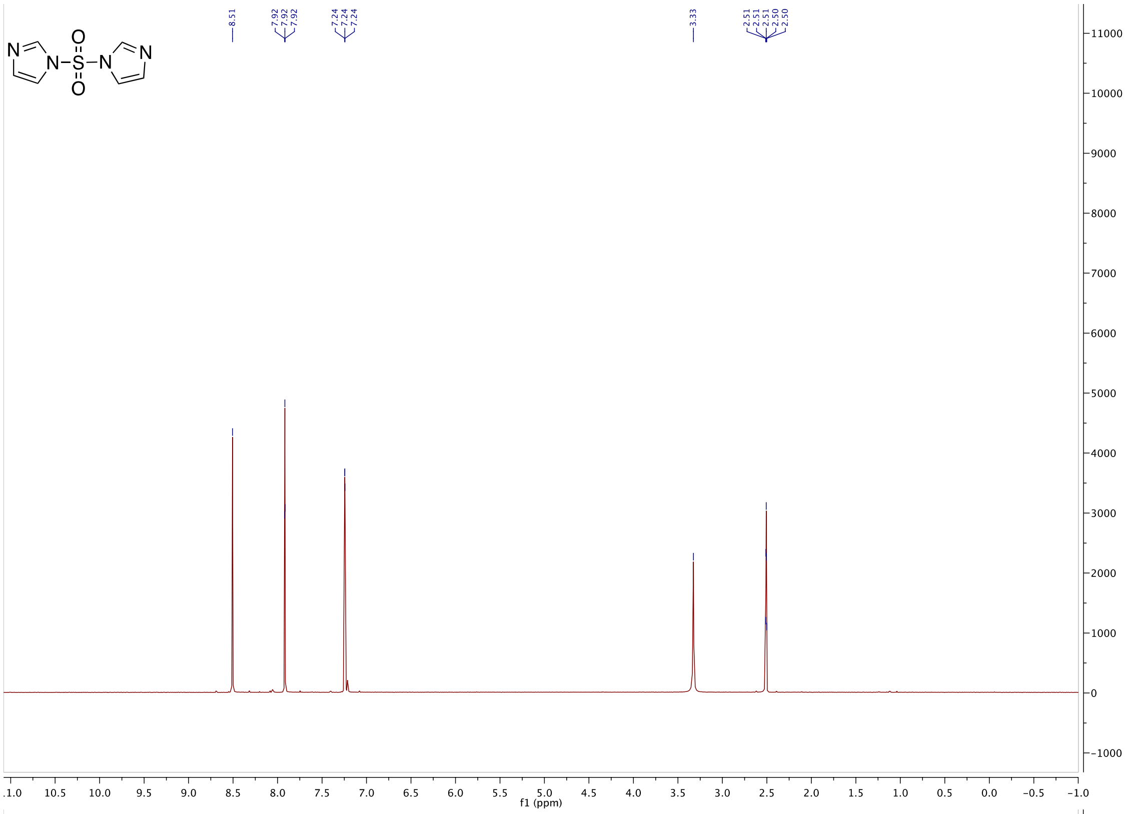

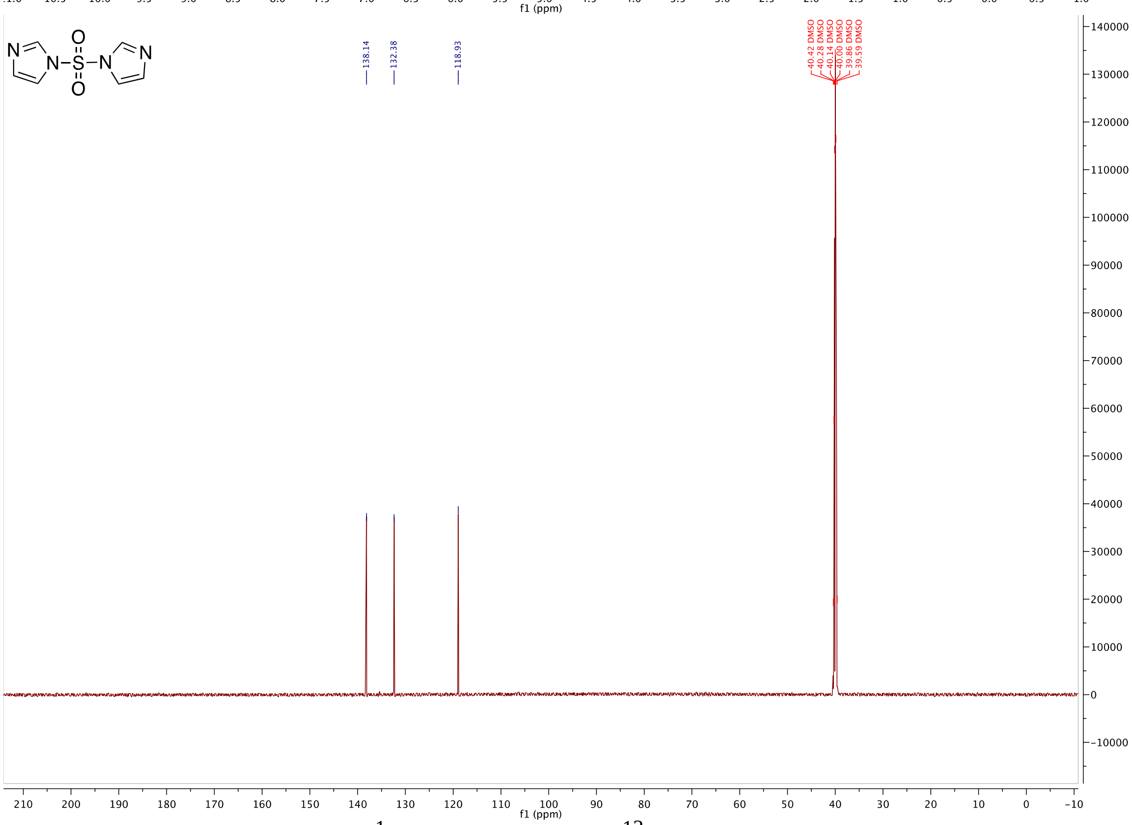

Supplementary Figure 12 | ^{1}H NMR (top) and ^{13}C NMR (bottom) of N,N' – sulfuryldiimidazole

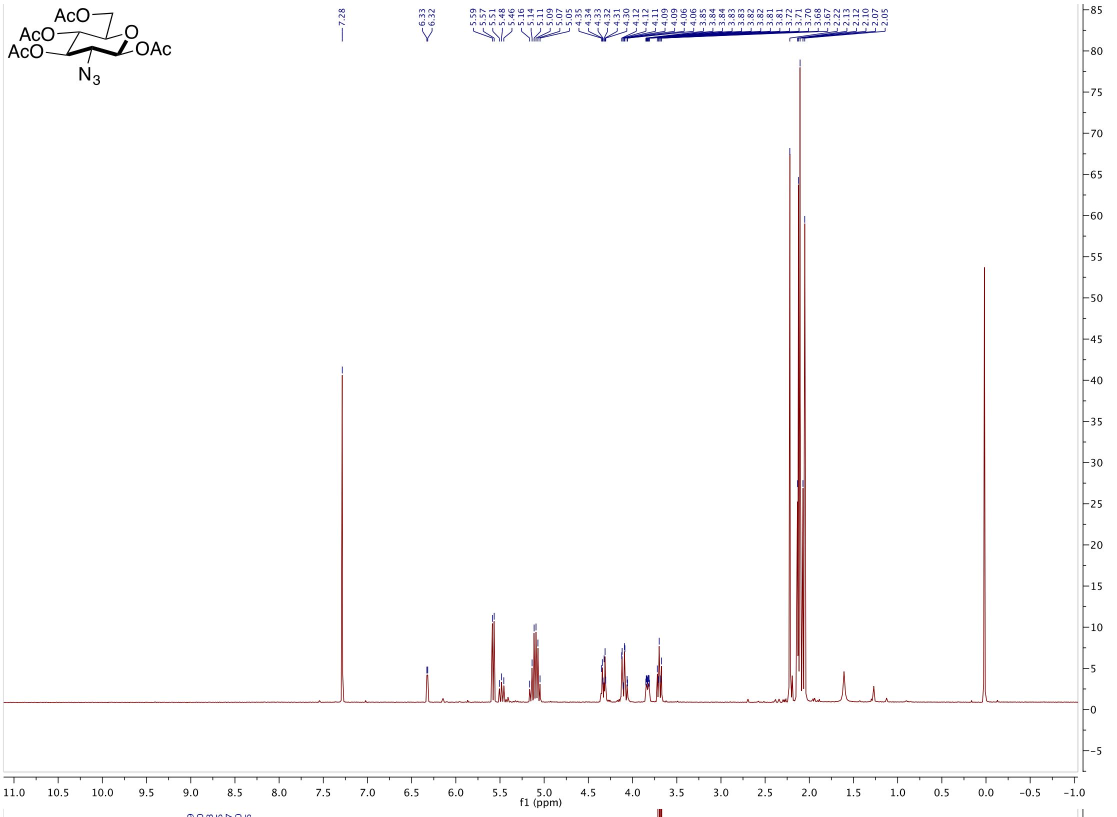

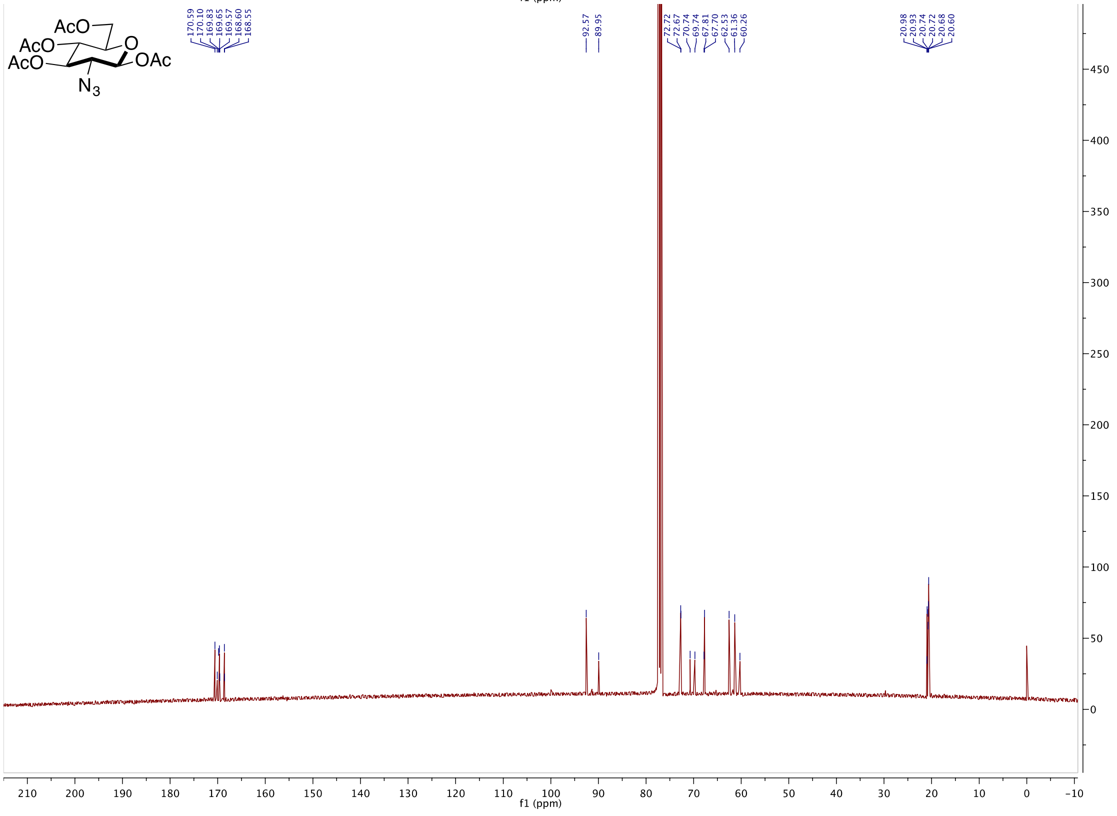

Supplementary Figure 13 | ^{1}H NMR (top) and ^{13}C NMR (bottom) of **4**

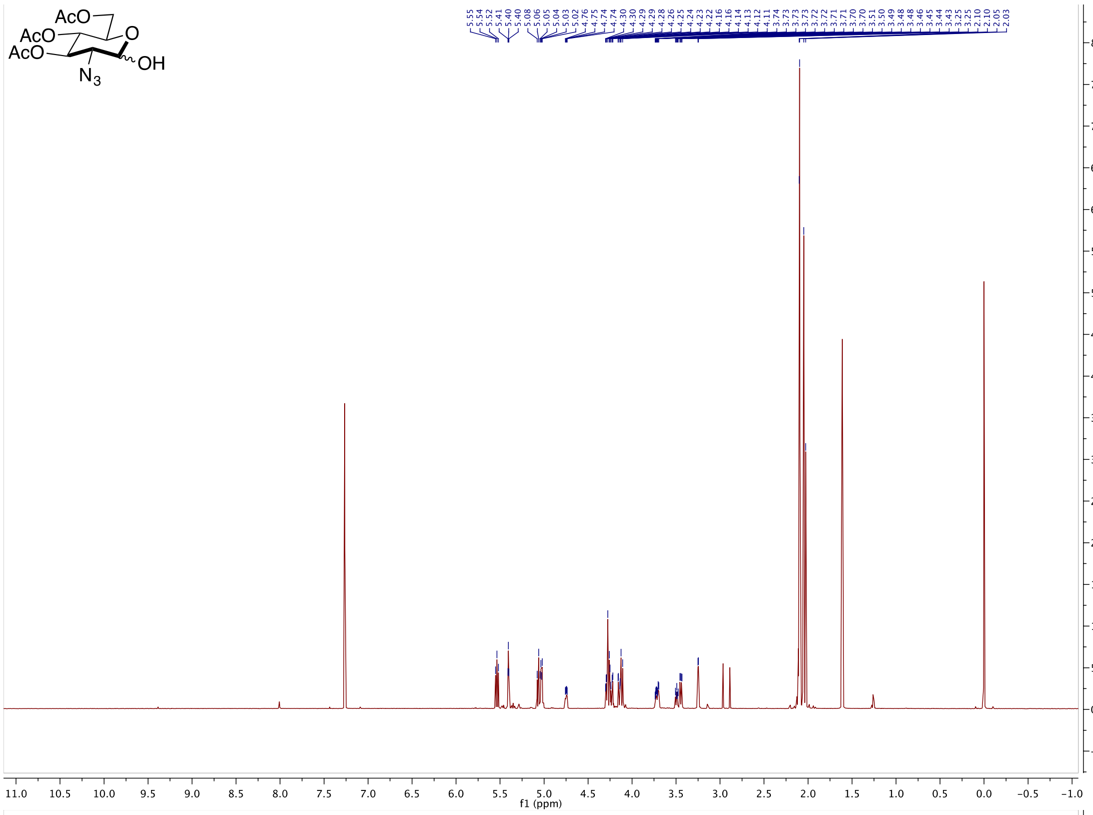

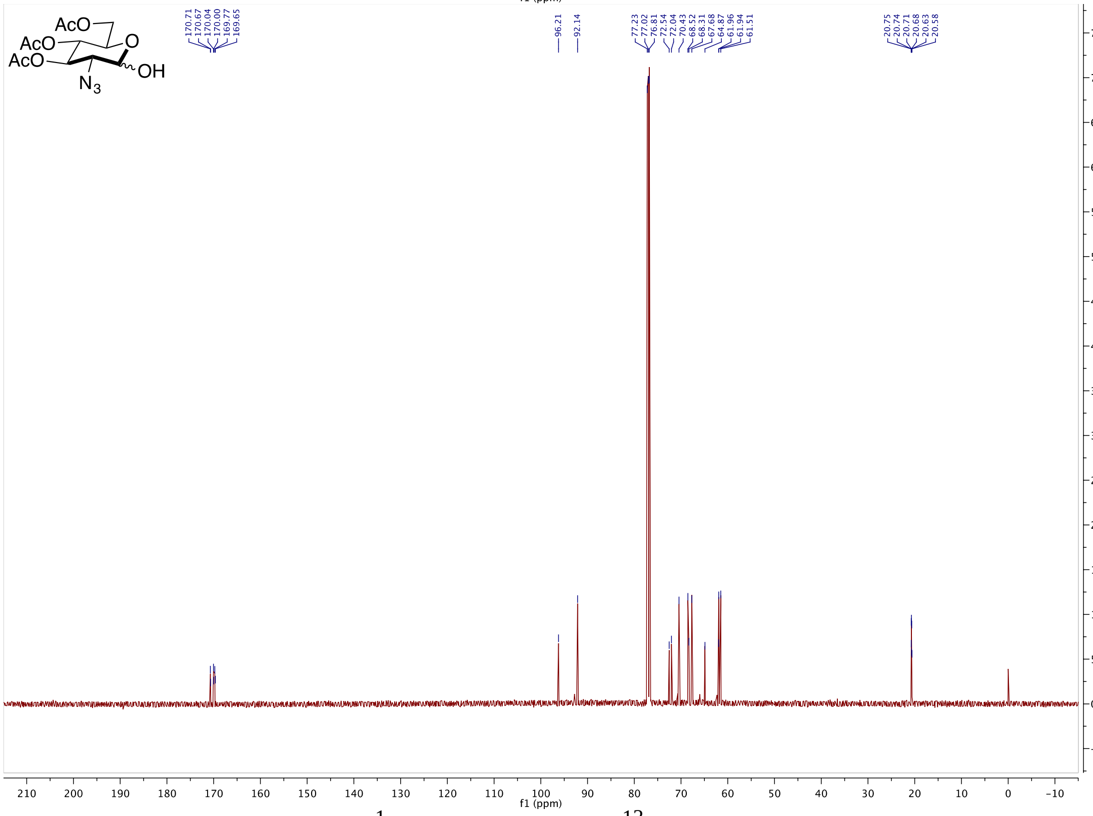

Supplementary Figure 14 | ^{1}H NMR (top) and ^{13}C NMR (bottom) of **5**

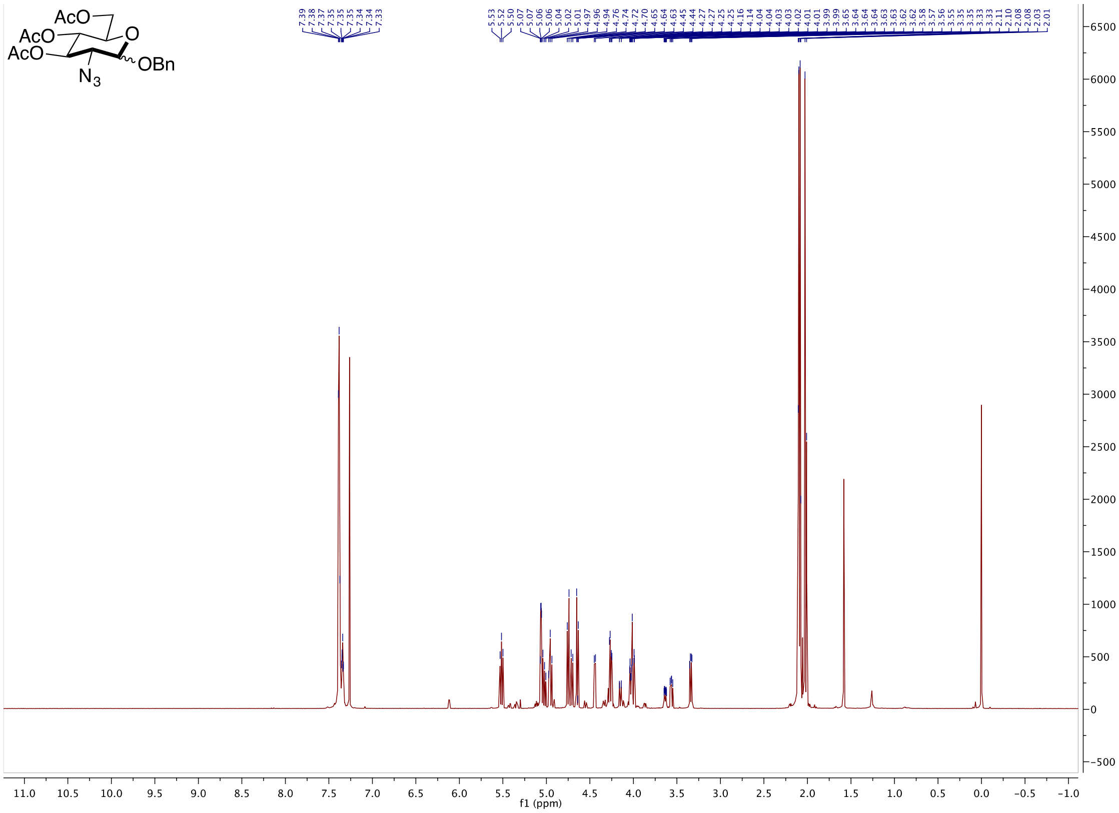

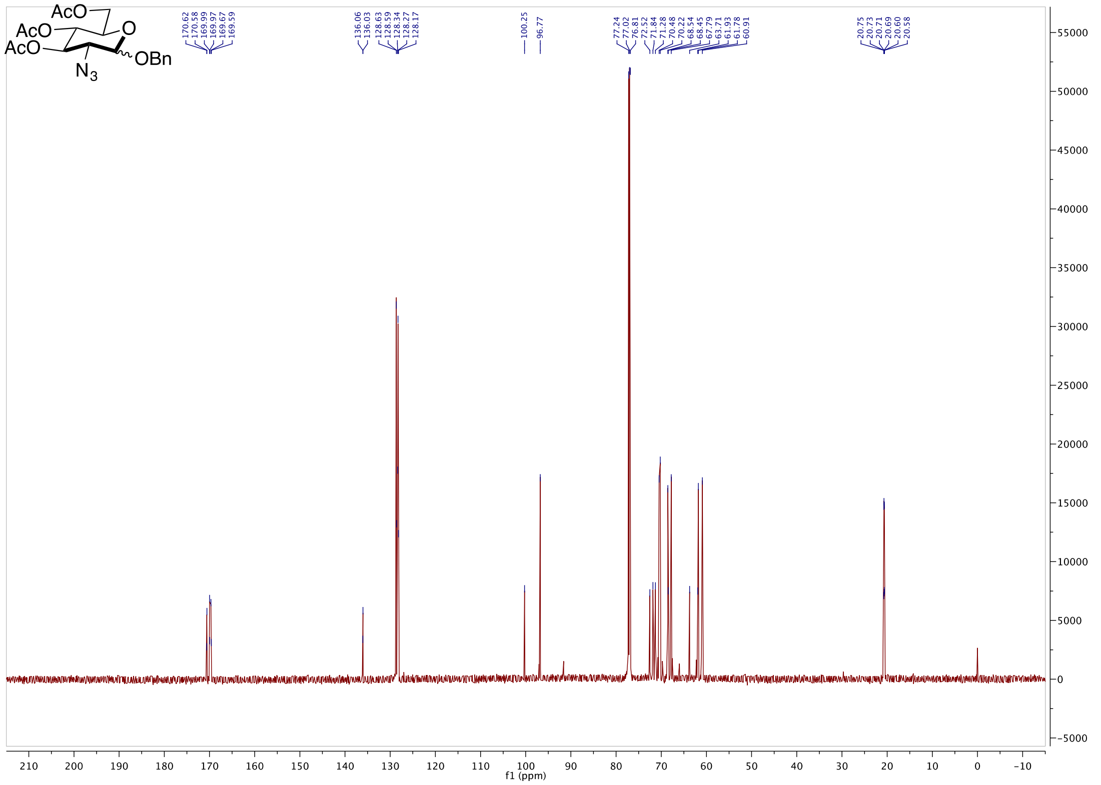

Supplementary Figure 15 | ^{1}H NMR (top) and ^{13}C NMR (bottom) of **6**

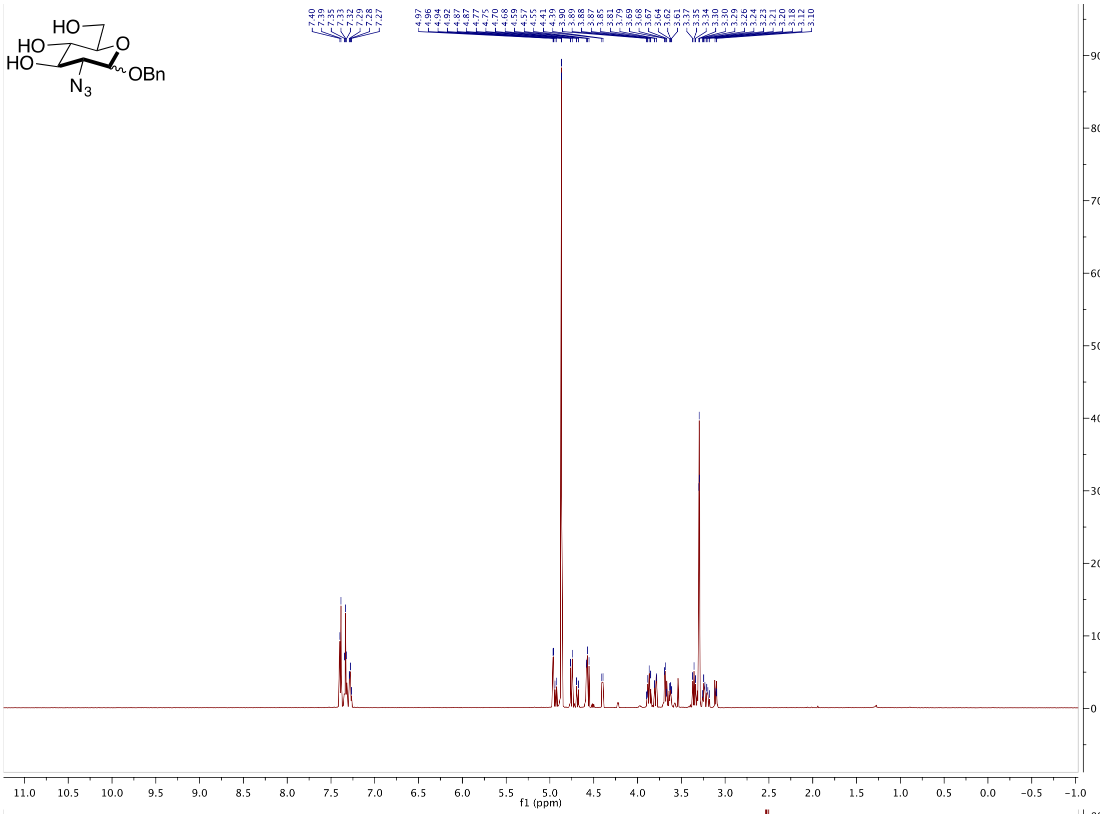

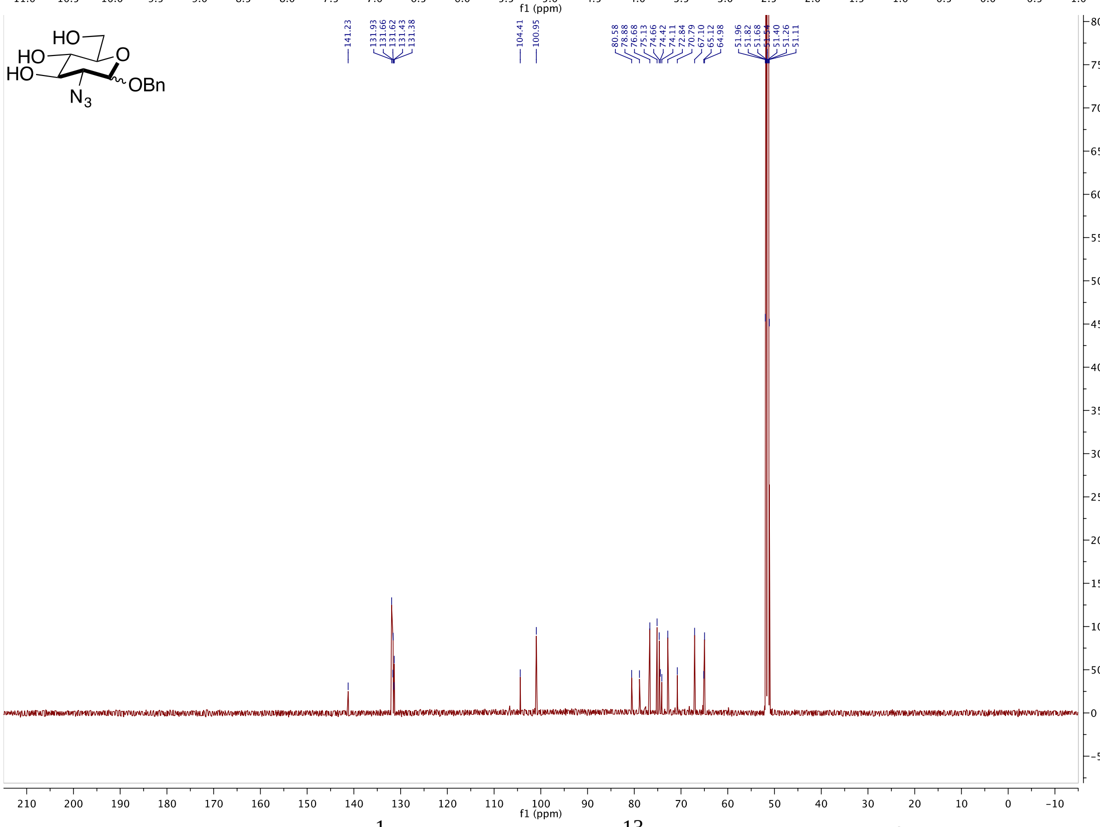

Supplementary Figure 16 | ^{1}H NMR (top) and ^{13}C NMR (bottom) of **7**

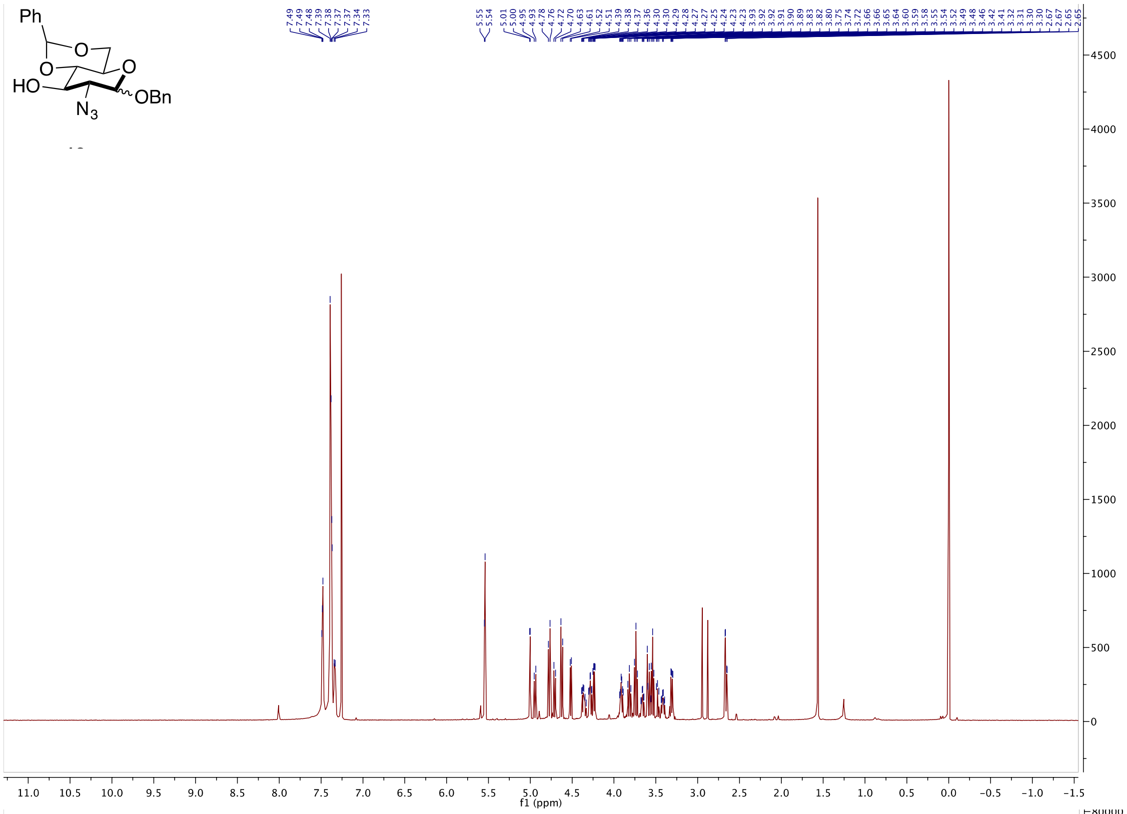

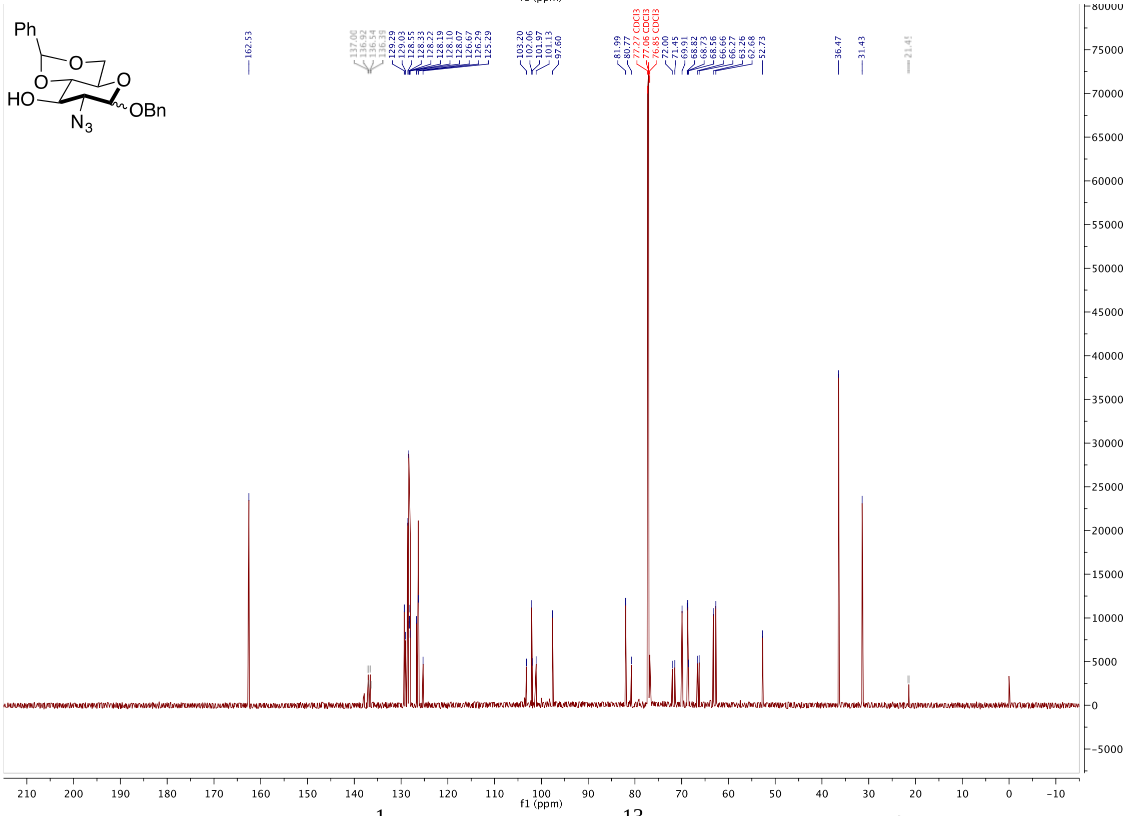

Supplementary Figure 17 | ^{1}H NMR (top) and ^{13}C NMR (bottom) of **8**

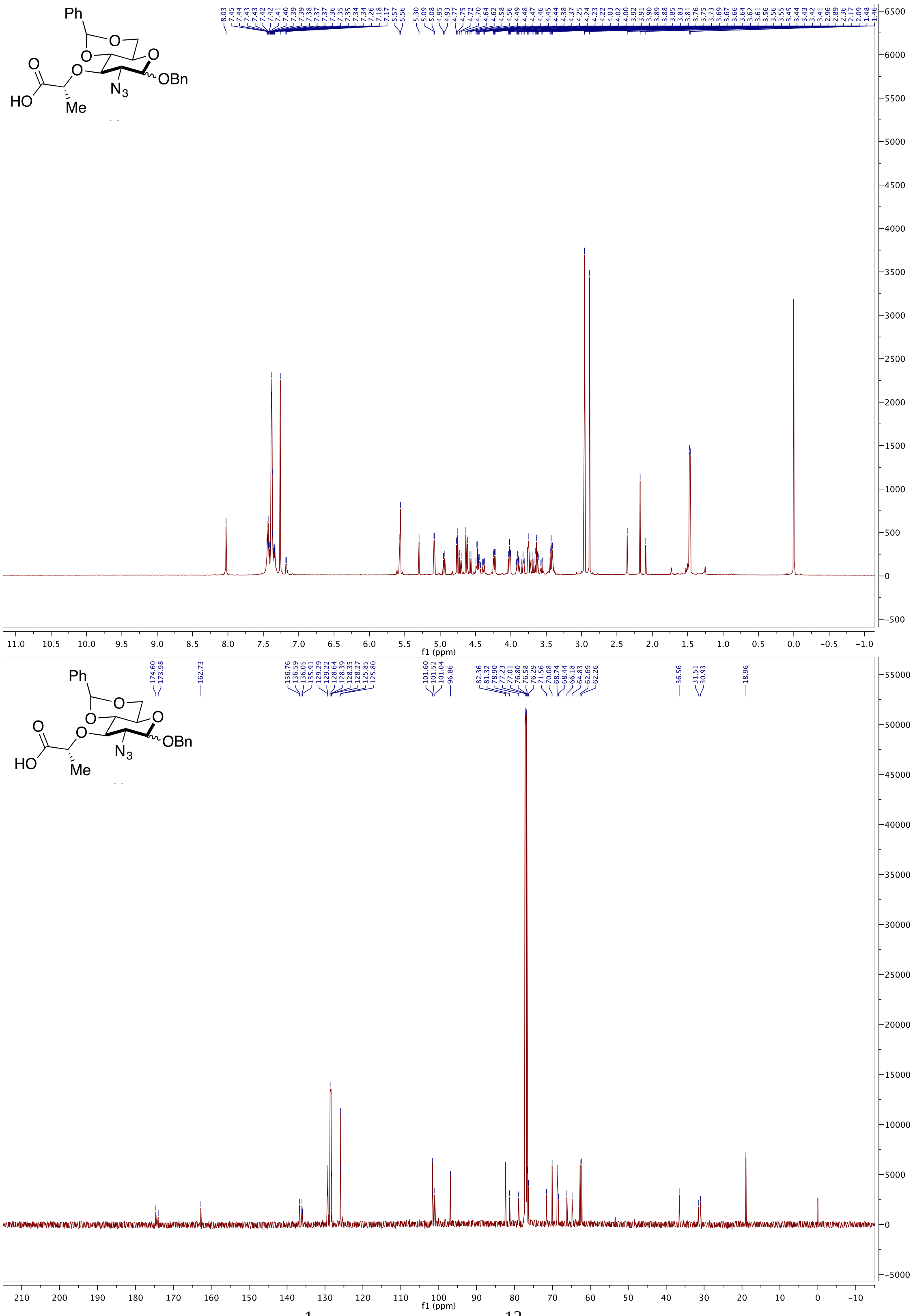

Supplementary Figure 18 | ^{1}H NMR (top) and ^{13}C NMR (bottom) of **9**

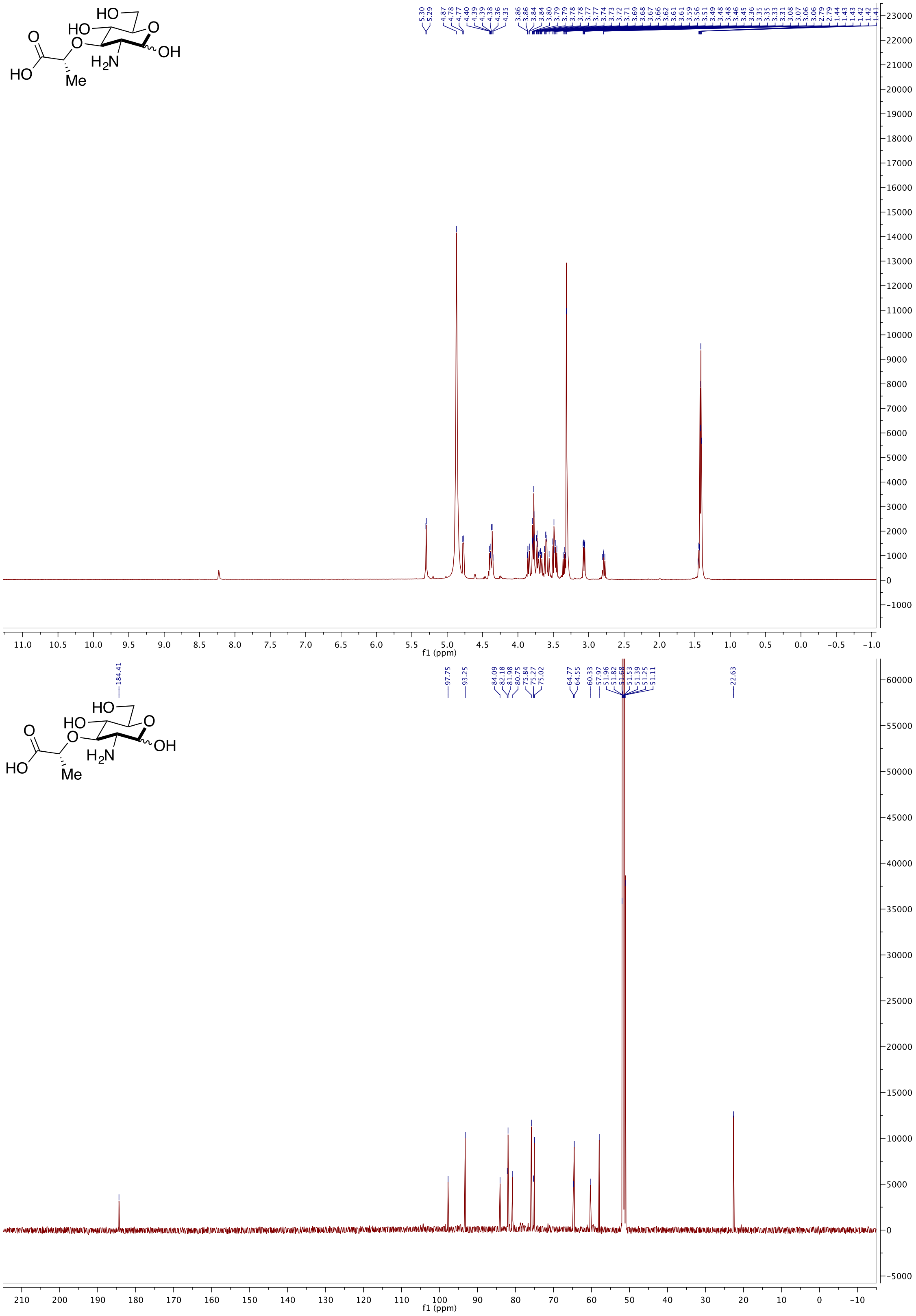

Supplementary Figure 19 | ^{1}H NMR (top) and ^{13}C NMR (bottom) of **10**

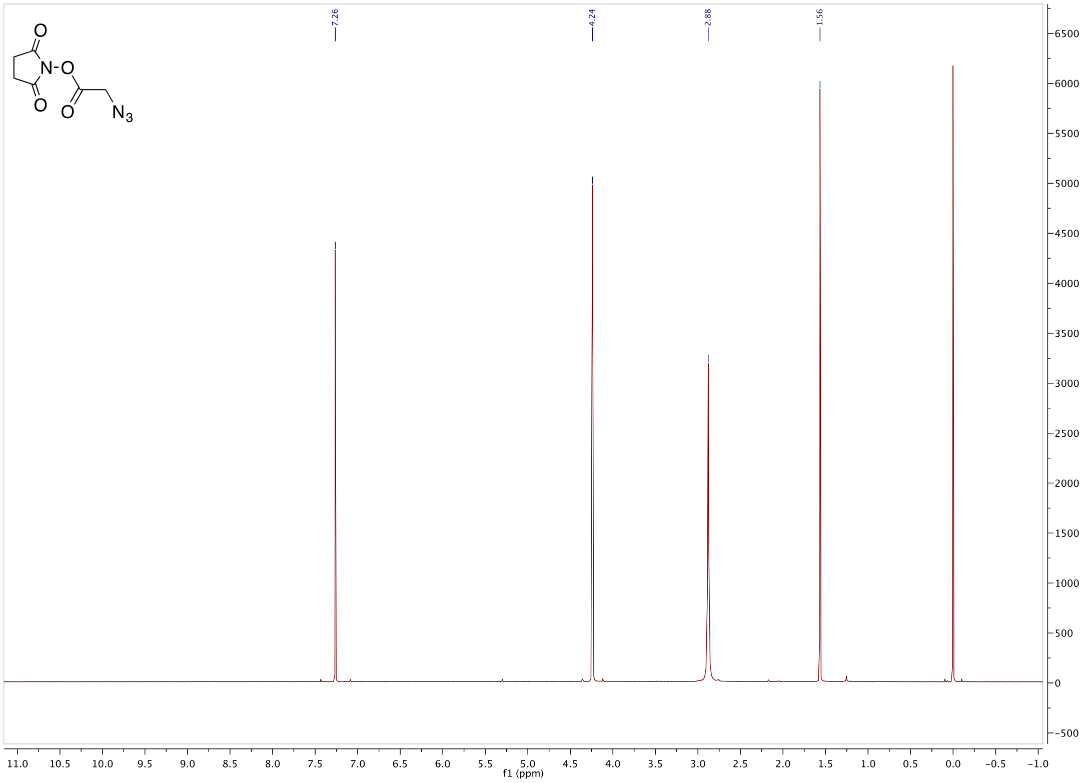

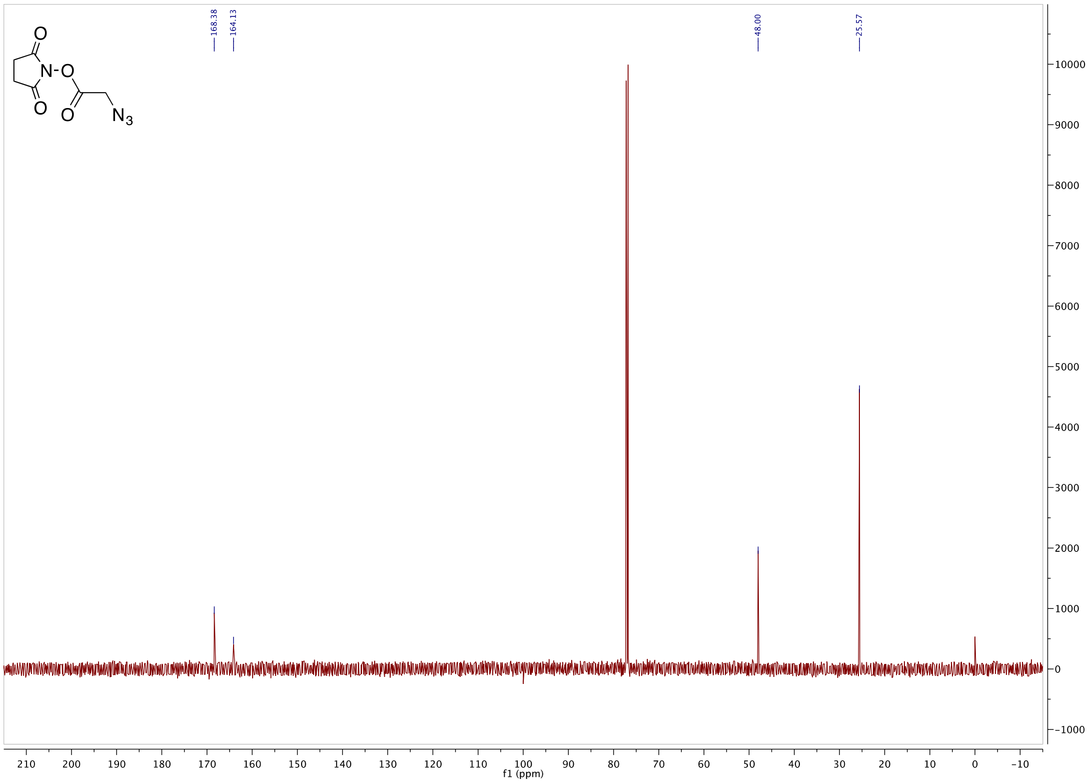

Supplementary Figure 20 | ^{1}H NMR (top) and ^{13}C NMR (bottom) of **11**

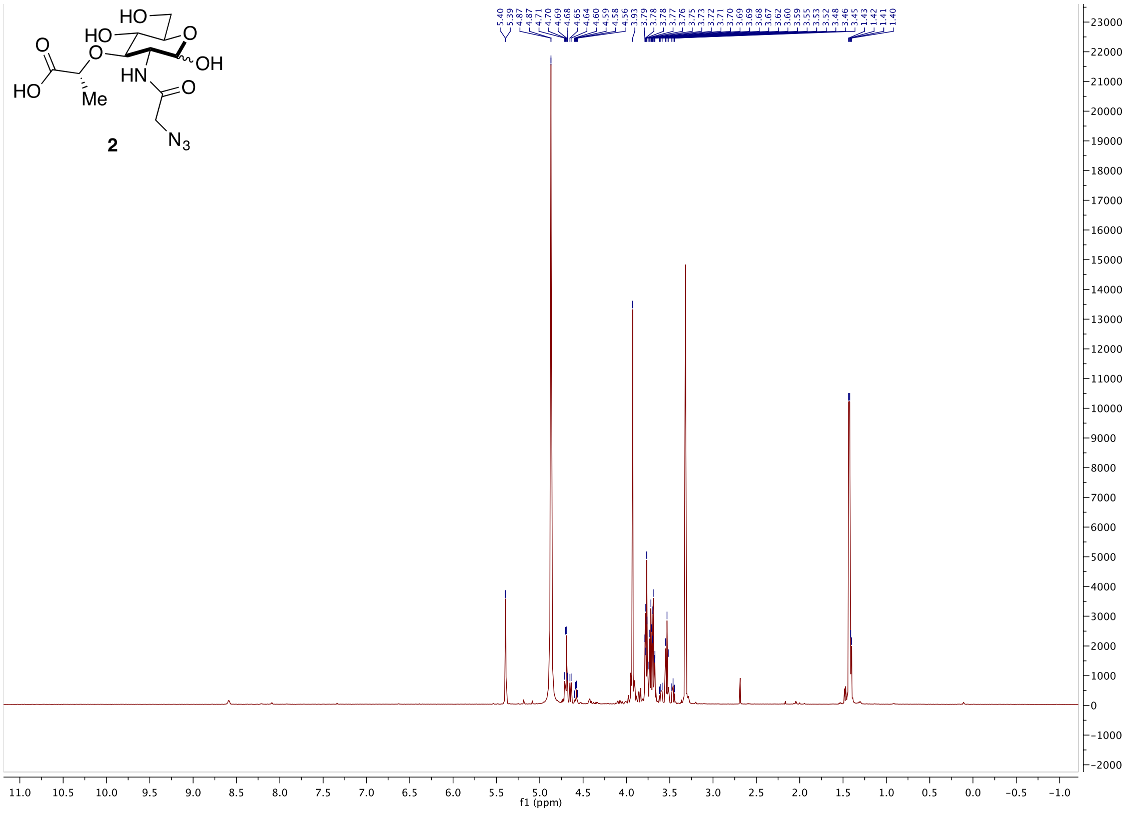

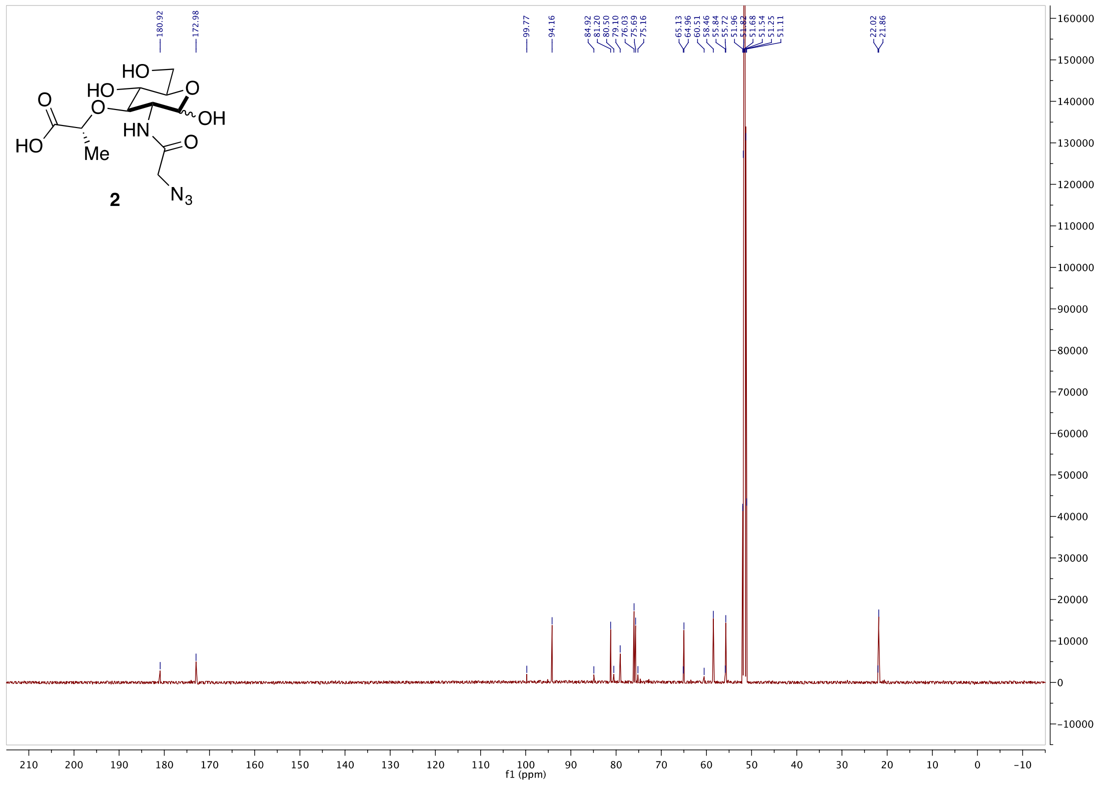

Supplementary Figure 21 | ^{1}H NMR (top) and ^{13}C NMR (bottom) of **2**

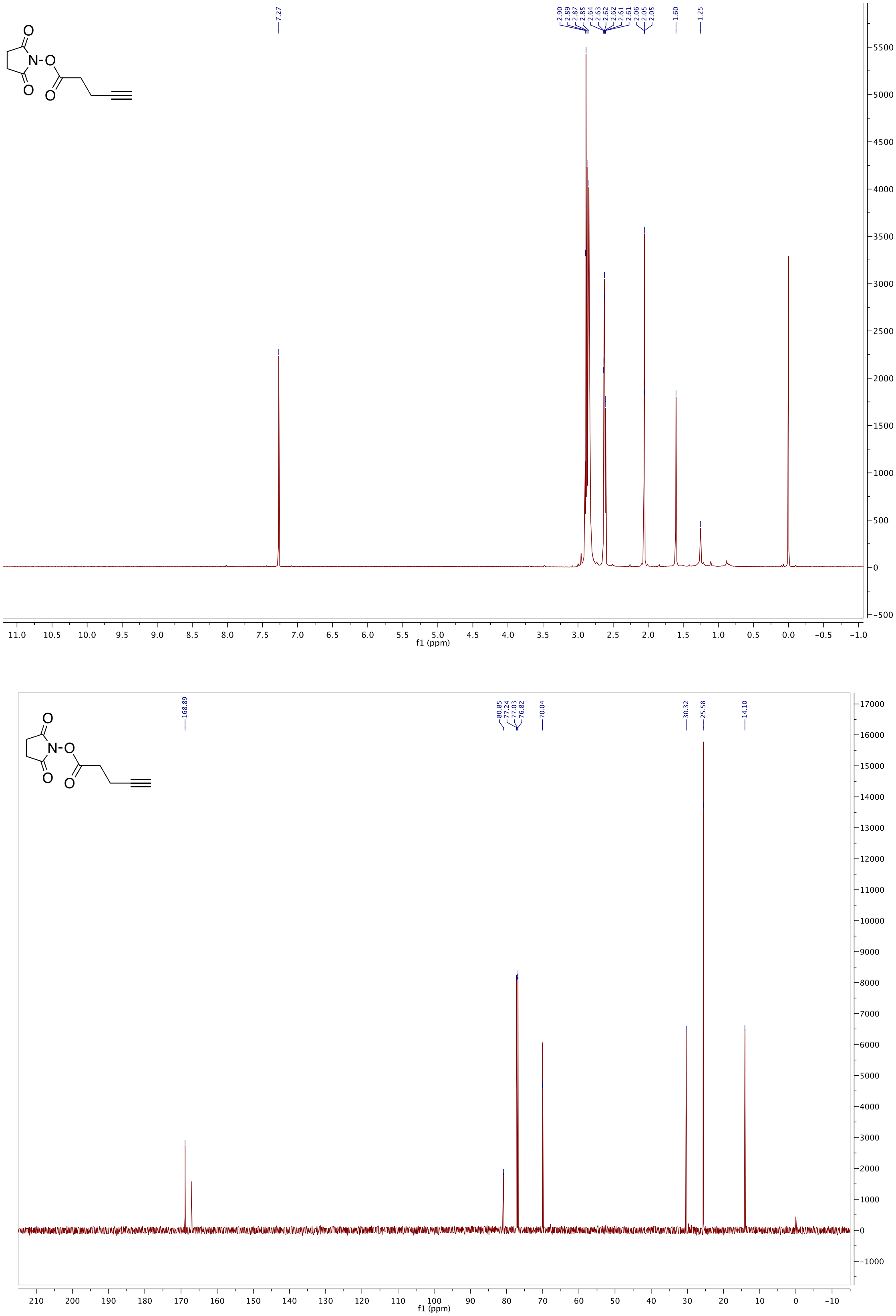

Supplementary Figure 22 | ^{1}H NMR (top) and ^{13}C NMR (bottom) of **12**

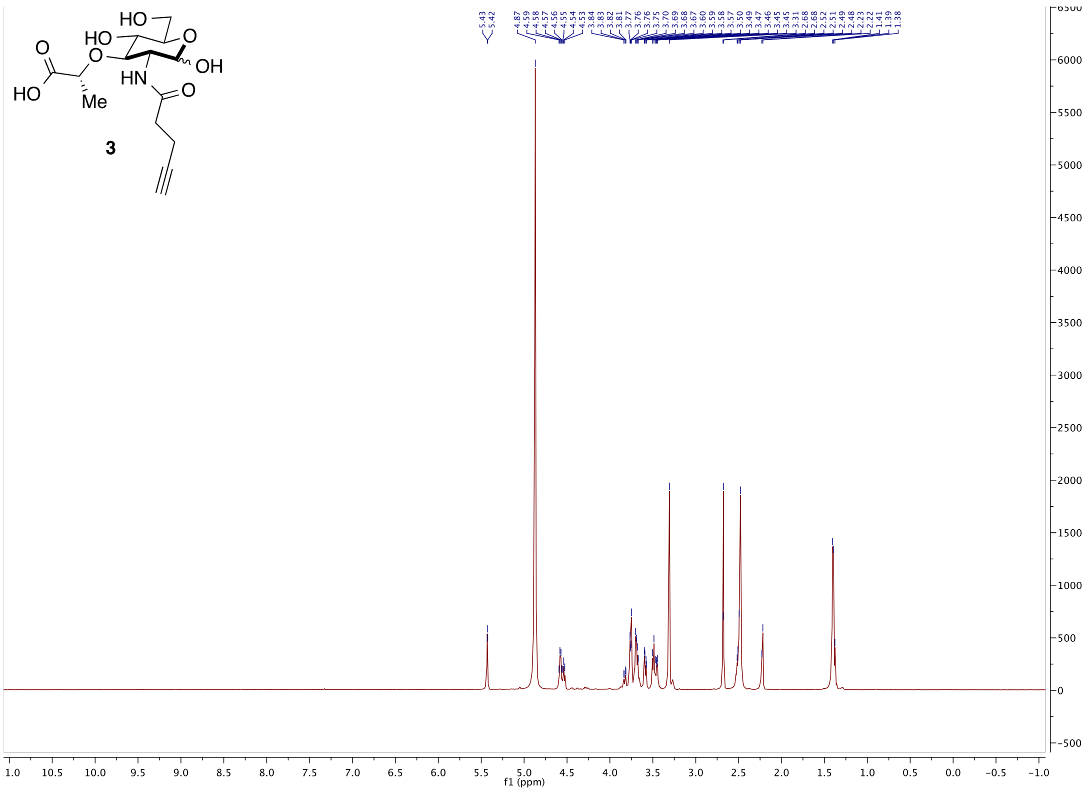

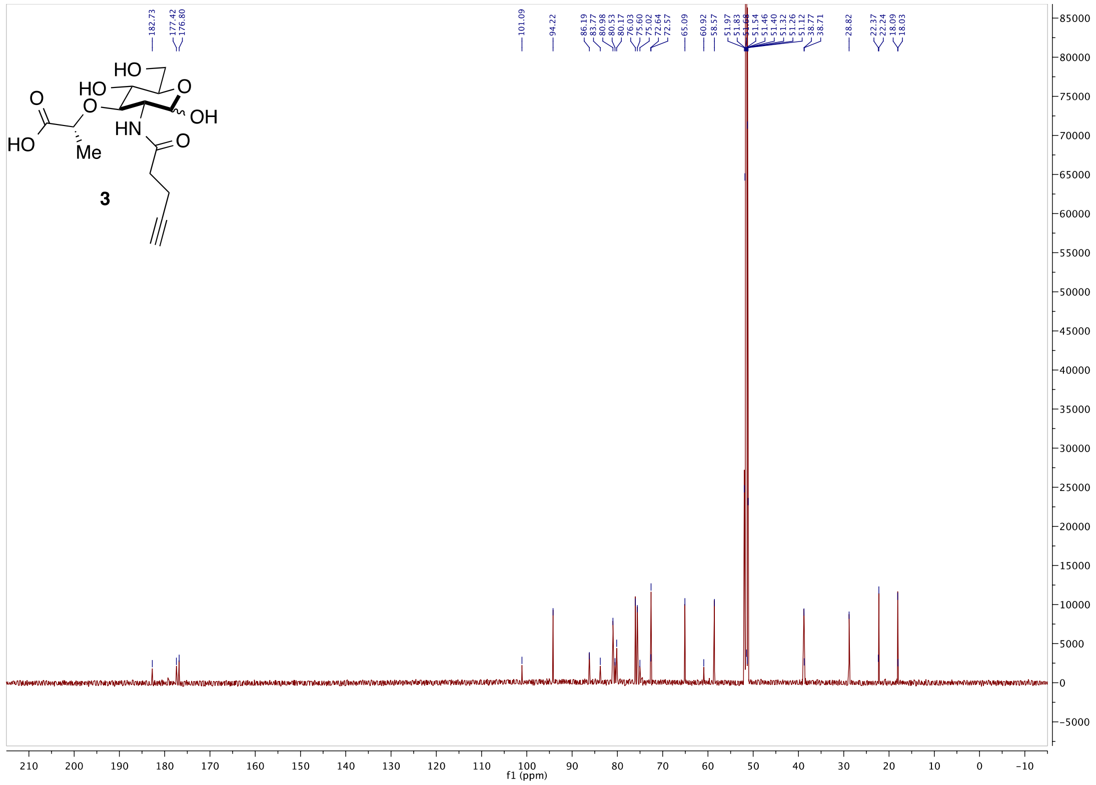

Supplementary Figure 23 | ^{1}H NMR (top) and ^{13}C NMR (bottom) of **3**

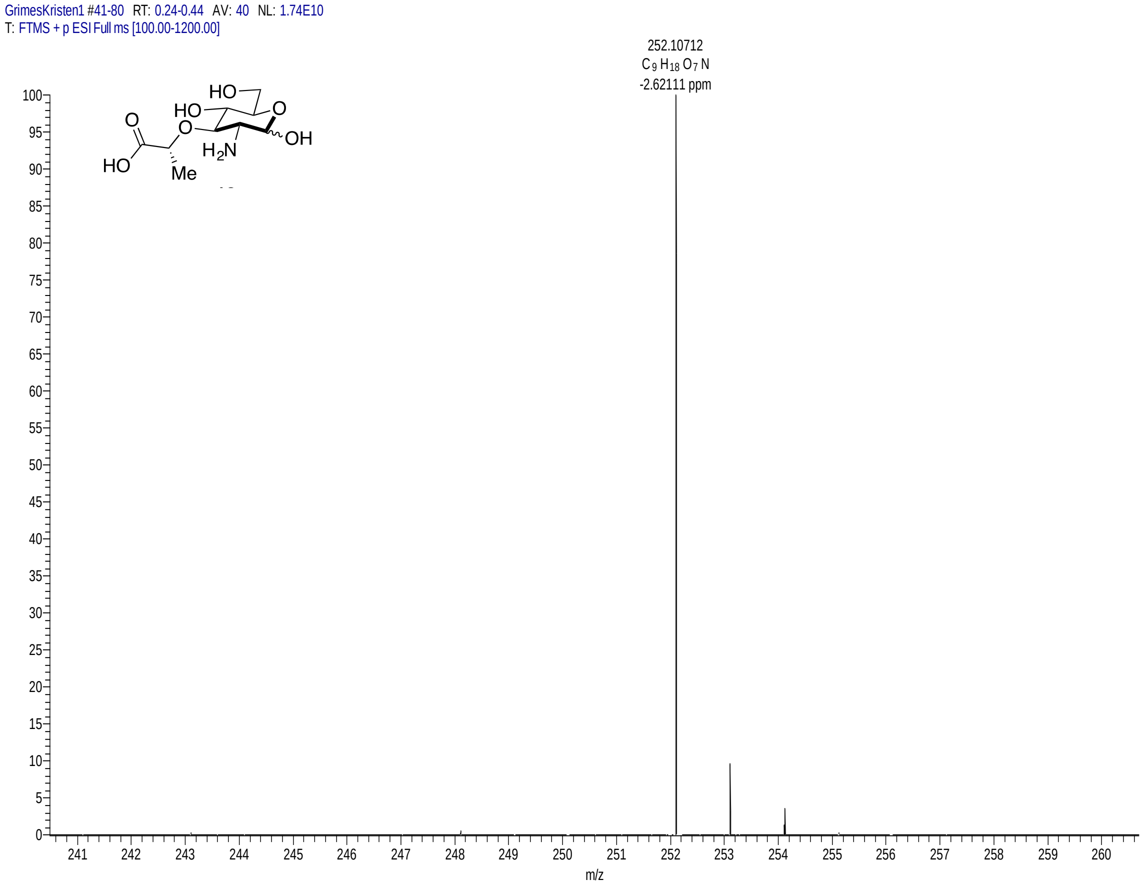

Supplementary Figure 24 | HRMS of **10**

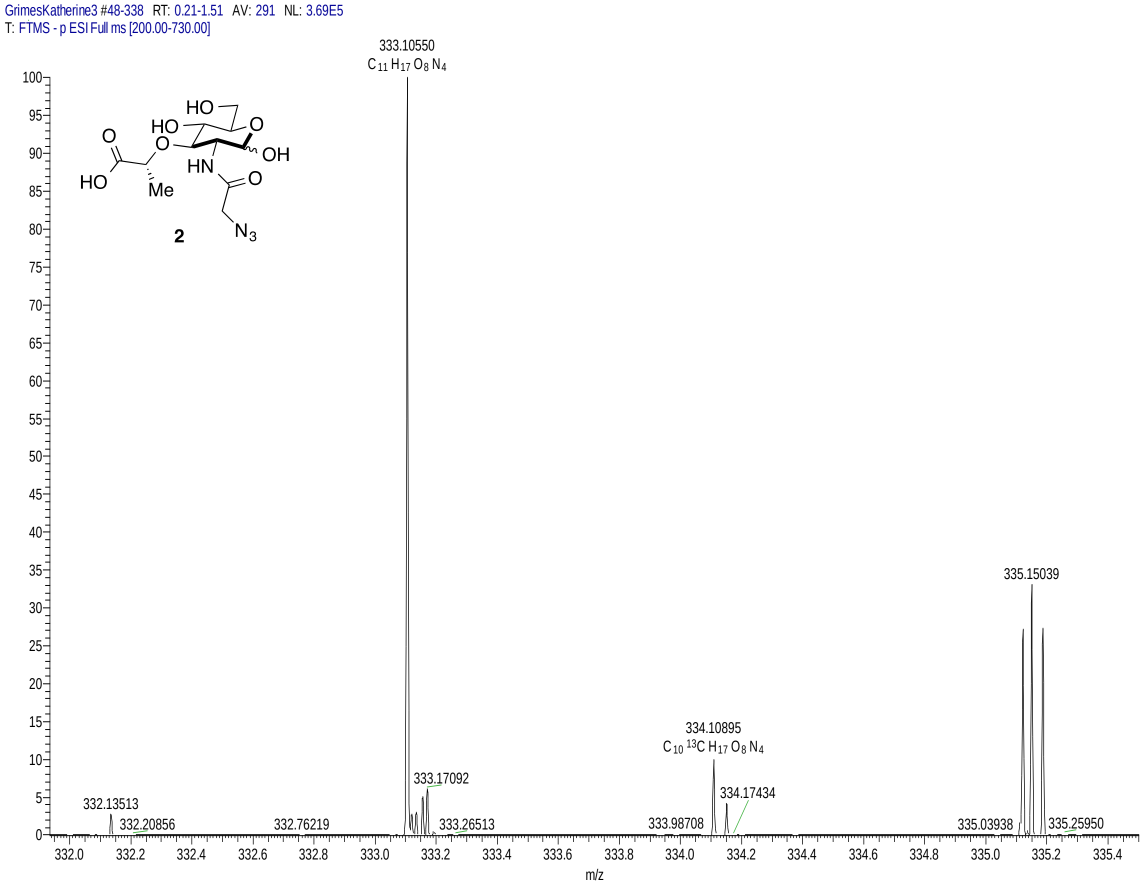

Supplementary Figure 25 | HRMS of **2**

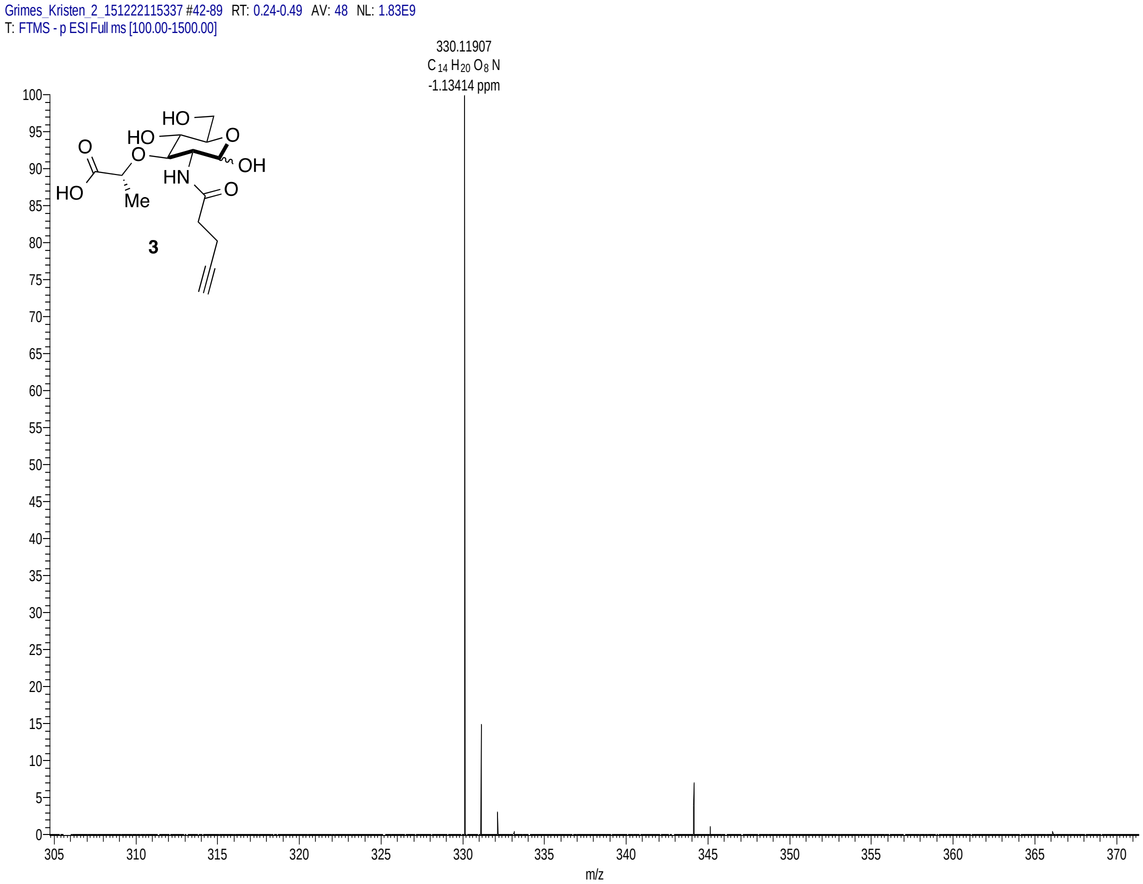

Supplementary Figure 26 | HRMS of **3**

Supplementary Table 1 | Characterization of chemoenzymatic intermediates. Library of a, UDP NAM (1b) and derivatives with b, azide (2b), c, alkyne (3b): *iii*) MurC, L-Alanine; *iv*) MurD, D-Glutamic Acid; *v*) MurE, *m*DAP; *vi*) MurF, D-Ala D-Ala. Tablulated high resolution LC/MS of compounds **(A)** 1a-1f, **(B)** 2a-2f, **(C)** 3a-3f.

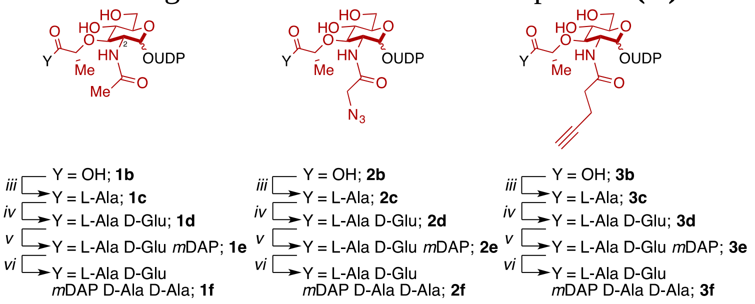

A

Substrate	Theoretical m/z [M-H]$^-$	Experimental m/z [M-H]$^-$	Mass Accuracy (ppm)
1a	372.07012	372.06973	-1.05664
1b	678.09542	678.09616	1.11018
1c	749.13254	749.13224	-0.39368
1d	878.17513	878.17506	-0.07446
1e	1050.25992	1050.26837	8.04613
1f	1192.33415	1192.34325	7.62979

B

Substrate	Theoretical m/z [M-H]$^-$	Experimental m/z [M-H]$^-$	Mass Accuracy (ppm)
2	333.10519	333.10550	1.02316
2a	413.07152	413.07118	-0.82734
2b	719.09682	719.09674	-0.11316
2c	790.13393	790.13352	-0.52473
2d	919.17653	919.17665	0.1337
2e	1091.26132	1091.26979	7.76217
2f	1233.33555	1233.34259	5.71541

C

Substrate	Theoretical m/z [M-H]$^-$	Experimental m/z [M-H]$^-$	Mass Accuracy (ppm)
3	330.11944	330.11907	-1.13414
3a	410.08577	410.08588	0.25584
3b	716.11107	716.11112	0.06551
3c	787.14819	787.14795	-0.30439
3d	916.19078	916.19134	1.81106
3e	1088.27557	1088.27551	-0.05489
3f	1230.3498	1230.34827	-1.23915

Supplementary Table 2. List of bacterial strains and plasmids

Strain	Genotype	Reference
Escherchia coli		
DH5α	*endA1 hsdR17* (r_K^-, m_K^-) *supE44 thi-1 recA1 gyrA*(Nalr) *relA1 Δ(lacZYA-argF)U169 deoR* φ80 *dlacΔ(lacZ)M15*	this study
BL21 (DE3)	*E. coli* B F^- *ompT gal dcm lon* $hsdS_B$(rB^-mB^-) λ(DE3)	this study
ΔmurQ	Keio Collection, ΔmurQ757::kan	[1]
E. coli ΔmurQ-KU	ΔmurQ strain with pBBR-KU plasmid	this study
E. coli ΔmurQ-pBBR	ΔmurQ strain with pBBR1MCS plasmid	this study
E. coli KU	DH5α strain with pBBR-KU plasmid	this study
Pseudomonas putida		
KT2440	Wild type, rmo- mod+	this study
Bacillus subtilis		
3A38	NCIB 3610 *comI*^{Q12L}	[2]
3A38 KU	3A38 strain with intergrated *amgK, murU*	this study

Plasmids	Description
pGEX-6P-1	Ap^r, expression vector, GST tag, lac promoter
pBBR1MCS	Cam^r, expression vector, lac promoter
pDG1662	*amyE*'...'*amyE*, Cam^r, Spc^r, intergration verctor for *B. subtilis*
pGEX-PpAmgK	*P. putida amgK* in pGEX-6P-1, BamHI, XhoI
pGEX-PpMurU	*P. putida murU* in pGEX-6P-1, BamHI, XhoI
pGEX-EcMurC	*E. coli murC* in pGEX-6P-1, SalI, NotI
pGEX-EcMurD	*E. coli murD* in pGEX-6P-1, EcoRI, XhoI
pGEX-EcMurE	*E. coli murE* in pGEX-6P-1, EcoRI, XhoI
pGEX-EcMurF	*E. coli murF* in pGEX-6P-1, BamHI, XhoI
pBBR-KU	*P. putida amgK, murU* in pBBR1MCS, KpnI, HindIII
pDG-KU	*P. putida amgK, murU* in pDG1662, BamHI, HindIII

Supplementary Table 3. | List of primer sequences.

Name	Sequence[a]
PpAmgK-For	GCTAGGATCCATGCCGGAACATGATG
PpAmgK-Rev	GATACTCGAGTTATGCACGTGCGCCTG
PpMurU-For	GCACTGGATCCATGAAAGCCATGATTC
PpMurU-Rev	GCATCCTCGAGTTATGCACGTTCACC
EcMurC-For	GACGGTCGACTCATGAATACACAACAAT TGG
EcMurC-Rev	ATACGCGGCCGCTCAGTCATGTTGTTCTTC
EcMurD-For	GAAGCGAATTCATGGCTGATTATCAGGG
EcMurD-Rev	AGTCGCTCGAGTCAACCTAACTCCTTCG
EcMurE-For	ATACGGAATTCATGACACTCGACAGCCG
EcMurE-Rev	ATAGCTCGAGTCATGCAATCACCCCCAG
EcMurF-For	GAGCGGATCCATGATTAGCGTAACCCTT AG
EcMurF-Rev	ATCGCTCGAGCTAACATGTCCCATTCTC
pBBRKU-For	GATAGGTACCATGACCCGCTTGACGGCT AG
pBBRKU-Rev	GTATAAGCTTTCAGGCGCGCTCGC
5GEX	GGGCTGGCAAGCCACGTTTGGTG
3GEX	CCGGGAGCTGCATGTGTCAGAGG
M13F(-21)	TGTAAAACGACGGCCAGT
M13R	CAGGAAACAGCTATGAC
pDGKU-For	GATAGGATCCTGACCCGCTTGACGGCTAG
pDGKU-Rev	GTATAAGCTTTCAGGCGCGCTCGC

[a]restriction sites are underlined.

Supplementary Table 4. Mean Fluorescence Intensities. Mean fluorescence intensity values and standard deviations (SD) from 3 technical replicates per sample. Mean FL4-H values used for Cy5 chromophores and Mean FL1-H values used for 488 chromophores. Calculations based upon equal population number.

**Figure**	**Sample**	**Mean FL4-H or FL1-H ± SD**
Supplementary Fig. 3b	WT cell	301.24 ± 24.73
Supplementary Fig. 3b	**1** + AlkCy5	425.69 ± 11.91
Supplementary Fig. 3b	**2** + AlkCy5	3065.64 ± 45.11
Supplementary Fig. 3c	WT cell	268.55 ± 10.24
Supplementary Fig. 3c	**1** + AzCy5	342.60 ± 7.34
Supplementary Fig. 3c	**3** + AzCy5	6995.80 ± 131.49
Supplementary Fig. 3d	WT cell	595.31 ± 164.26
Supplementary Fig. 3d	**1** + Alk488	506.32 ± 22.64
Supplementary Fig. 3d	**2** + Alk488	5672.13 ± 198.11
Supplementary Fig. 3e	WT cell	339.40 ± 13.57
Supplementary Fig. 3e	**1** + Az488	271.71 ± 6.53
Supplementary Fig. 3e	**3** + Az488	3286.41 ± 57.91
Supplementary Fig. 5c	WT cell	286.55 ± 10.24
Supplementary Fig. 5c	**1** + AzCy5, Fos	342.60 ± 7.34
Supplementary Fig. 5c	**3** + AzCy5, no Fos	3415.44 ± 17.75
Supplementary Fig. 5c	**3** + AzCy5, Fos	6661.13 ± 279.18
Supplementary Fig. 5c	WT cell	254.99 ± 1.08
Supplementary Fig. 5d	**1** + AzCy5	299.99 ± 1.18
Supplementary Fig. 5d	**1** + AzCy5, Lyz	285.88 ± 5.72
Supplementary Fig. 5d	**3** + AzCy5, Lyz	3735.65 ± 164.02
Supplementary Fig. 5d	**3** + AzCy5	6080.5 ± 116.78
Supplementary Fig. 8b	WT cell	265.44 ± 10.34
Supplementary Fig. 8b	**1**, 45 min	367.77 ± 8.65
Supplementary Fig. 8b	**3**, 15 min	1124.32 ± 22.21
Supplementary Fig. 8b	**3**, 30 min	2924.81 ± 34.65
Supplementary Fig. 8b	**3**, 45 min	3240.56 ± 32.20
Supplementary Fig. 8b	**3**, 60 min	3064.56 ± 42.42

Supplementary Note. Fluorophore chemical structures and abbreviations

AzCy5 = Cy5-azide, Sigma

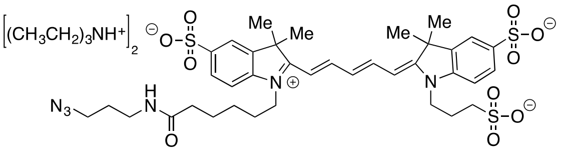

AlkCy5 = Cy5-alkyne, Sigma

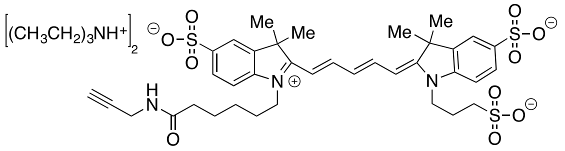

Az488 = Rhodamine 110 Azide (Azide Fluor 488, Sigma)

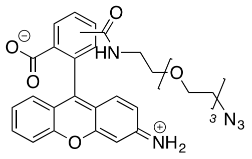

Alk488 = Rhodamine 110-PEG4-alkyne (Fluor 488-Alkyne, Sigma)

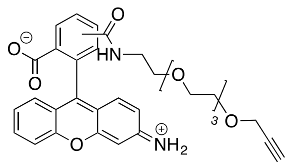

Supplementary Methods.

Materials.

All reagents were purchased from Sigma Aldrich, Fisher Scientific, Alfa Aesar or Invitrogen and used without further purification, unless otherwise noted. NMR solvents were purchased from Cambridge Isotope Laboratories, Inc.

Synthetic Procedures.

General Procedures. Unless otherwise noted, all reactions were performed in flame or oven dried flasks equipped with rubber septa, positive pressure of nitrogen, and magnetic stirring. All solvents were anhydrous and transferred via stainless steel syringe or cannula. Reactions were monitored by electrospray ionization liquid chromatography mass spectrometry (ESI LC-MS) and thin layer chromatography (TLC) in which glass plates coated with silica gel (250 μm, Silica Gel HL, Sorbent Technologies) were used and visualized with shortwave 254 nm UV light or developed upon heating with *p*-anisaldehyde or $KMnO_4$. Flash chromatography was carried out on silica gel (60 Å, 40-63 μm, Sorbent Technologies). Analytical and semi-preparative HPLC was performed on an Agilent Series 1100 using a Phenomenex® Luna 5 μm C18 column (250 x 10.00 nm). Preparative HPLC purification was performed on a Waters 2767 Sample Manager with HPLC and SQD2 MS using a Sunfire® Prep C18 OBD 5μm 19x100mm or 4.6x50mm columns.

Instrumentation. All NMR spectra were recorded on Bruker AV 400 MHz and AV III 600 MHz spectrometers. Proton chemical shifts were recorded in parts per million (ppm) on the δ scale, downfield from tetramethylsilane and referenced from an internal standard

of residual protium in the NMR solvents ($CHCl_3$: δ 7.26, D_2HCOD 3.30). Data for ^{13}C NMR were reported in ppm downfield from tetramethylsilane and referenced based on the chemical shift from the carbon resonances of the solvent ($CDCl_3$: δ 77.16, CD_3OD 49.0). NMR data were reported as follows: chemical shift, multiplicity (s = singlet d = doublet, t = triplet, q = quartet, m = multiplet, br = broad), coupling constant in Hz, integration, and assignment based on two dimensional COSY, HSQC and HMBC experiments. Low-resolution mass spectra (LRMS) were obtained on a Shimadzu LCMS 2020 instrument using electrospray ionization (ESI), while high-resolution mass spectra (HR-MS), ESI mode, were obtained on a Thermo Q-Exactive Orbitrap at the Mass Spectroscopy Facility at the Department of Chemistry, University of Delaware. IR spectra were obtained on an IR100 with an ATR probe. Purity of novel compounds was assessed quantitatively through analytical HPLC/MS on a Waters 2767 Sample Manager with HPLC and SQD2 MS using a Sunfire® C18 5μm 6x50mm column. Flow Cytometry experiments were performed on an Accuri C6 Flow Cytometer. Structured Illumination Microscopy (SIM) images were taken on a Zeiss Elyra PS1 super-resolution microscope at the Delaware Biotechnology Institute Bioimaging Center. 3D STORM was performed at Carl Zeiss Microscopy, LLC One Zeiss Drive Thornwood, NY 10594.

Synthetic Procedures: Synthesis of NAM derivatives **2-3**

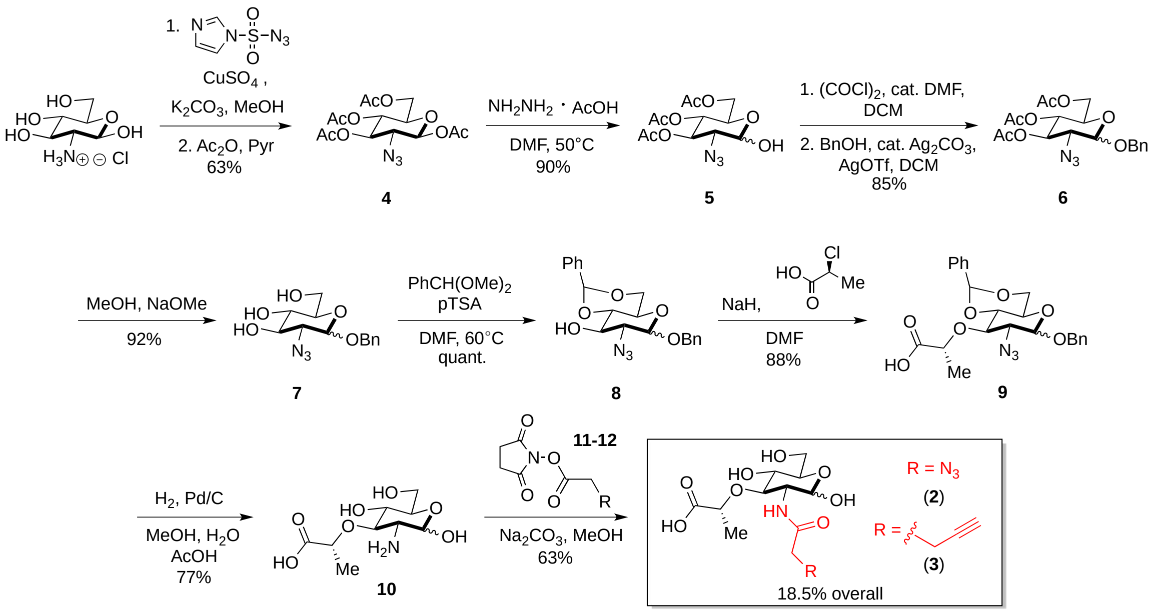

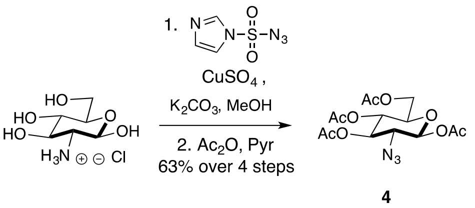

4

(2*S*,3*R*,4*R*,5*S*,6*R*)-6-(acetoxymethyl)-3-azidotetrahydro-2*H*-pyran-2,4,5-triyl triacetate (4)

Preparation of diazotransfer reagent imidazole-1-sulfonyl azide[3]:

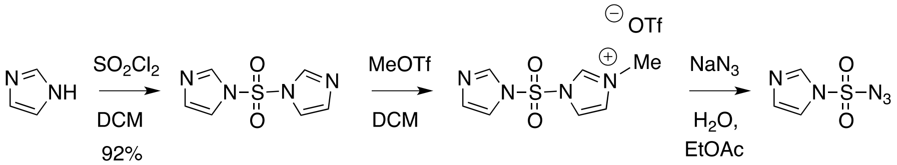

To a solution of imidazole (79.00 g, 1.17 mol, 4.75 eq) in 633 mL of anhydrous dichloromethane was added a solution of sulfuryl chloride (20.0 mL, 0.25 mol, 1.0 eq) in 119 mL of anhydrous dichloromethane via cannulae transfer at 0°C under N_2. Reaction warmed to room temperature and continued to stir under N_2 for 16 hours. The reaction was filtered and the filtrate condensed under reduced pressure to yield and off white solid. The solid was recrystallized in 80 mL of refluxing isopropanol to yield N,N' – sulfuryldiimidazole as a colorless crystalline solid (44.97 g, 92%). ^{1}H NMR (600 MHz, DMSO-d6) δ 8.51 (s, 2 H, H-1), 7.92 (t, J = 1.5 Hz, 2 H, H-2), 7.26 – 7.24 (m, 2 H, H-3). ^{13}C NMR (151 MHz, DMSO-d6) δ 138.14 (C-1), 132.38 (C-2), 118.93 (C-3).

N,N' – sulfuryldiimidazole (5.00 g, 25.2 mmol, 1.0 eq) was then suspended in 50 mL of anhydrous dichloromethane at 0°C under N_2. Methyl trifluoromethanesulfonate (2.56 mL, 22.7 mmol, 0.9 eq) was added dropwise over 15 minutes at 0°C. The reaction stirred at 0°C for 2 hours. The solvent was decanted off and 3-(imidazole-1-sulfonyl)-1-methyl-3H-imidazol-1-ium triflate was isolated as a white solid was washed three times each with 50 mL of cold dichloromethane and dried under high vacuum for 10 minutes and immediately used in the next reaction.

3-(Imidazole-1-sulfonyl)-1-methyl-3H-imidazol-1-ium triflate (9.31 g, 25.2 mmol, 1.0 eq) was dissolved in 30 mL of deionized H_2O followed by 30 mL of ethyl acetate at 0°C. This solution stirred at 0°C for 30 minutes. NaN_3 (1.97 g, 30.24 mmol, 1.2 eq) was added slowly and the reaction mixture stirred at 0°C for 1 hour. The phases were separated and the organic layer was collected, dried over Na_2SO_4, and filtered. The filtrate containing the imidazole-1-sulfonyl azide was used directly in the diazotransfer reaction without further purification.

Preparation of (2*S*,3*R*,4*R*,5*S*,6*R*)-6-(acetoxymethyl)-3-azidotetrahydro-2*H*-pyran-2,4,5-triyl triacetate (4). To the imidazole-1-sulfonyl azide ethylacetate solution (40 mL,

25.2 mmol) was added sequentially D glucosamine HCl (6.52 g, 30.2 mmol, 1.2 eq), 82 mL of anhydrous methanol, K_2CO_3 (6.27 g, 45.4 mmol, 1.8 eq), and anhydrous $CuSO_4$ (0.0483g, 0.302 mmol, 0.012 eq) at room temperature under N_2. The reaction continued to stir at room temperature for 16 hours. The reaction was filtered over celite and washed with 20 mL methanol. The solvent was then evaporated under reduced pressure and dried on the high vacuum overnight to yield a light yellow foam. To the light yellow foam was added 52 mL of anhydrous pyridine at 0°C under N_2. To this mixture was added a solution of Ac_2O (17 mL, 176 mmol, 7.0 eq) and DMAP (0.2709 g, 2.22 mmol, 0.088 eq) dropwise at 0°C. The reaction warmed slowly to room temperature and continued to stir for 20 h. Product formation was confirmed by TLC (3:2 hexanes : ethyl acetate r_f: 0.5) with PAA staining. The reaction mixture was diluted with 100 mL of deionized water. The water layer was extracted three times with ethyl acetate (200 mL total). The organic layers were combined and washed three times with 1N HCl. The organic layer was dried over Na_2SO_4, filtered, and condensed. The brown oily residue was purified by flash chromatography 3:2 hexanes : ethyl acetate to yield tan foam (5.88g, 63% over 4 steps). ^{1}H NMR (400 MHz, Chloroform-*d*) (Anomers 0.67α : 1.00β) δ 6.32 (d, *J* = 3.7 Hz, 1 H, H-1α), 5.58 (d, *J* = 8.6 Hz, 1 H, H-1β), 5.52 – 5.45 (m, 1 H, H-3α), 5.09 (dt, *J* = 17.9, 9.4 Hz, 2 H, H-3β, H-4β), 4.33 (dt, *J* = 12.6, 3.8 Hz, 2 H, H-6'α, H-6'β), 4.09 (td, *J* = 12.4, 2.1 Hz, 2 H, H-5α, H-6β), 3.83 (ddt, *J* = 8.0, 4.3, 2.0 Hz, 2 H, H-5β, H-6α), 3.73 – 3.67 (m, 2 H, H-2α, H-2β), 2.22 (s, 4H, Ac), 2.13 (s, 2H, Ac), 2.12 (s, 3 H, Ac), 2.10 (s, 4 H, Ac), 2.07 (s, 2 H, Ac), 2.05 (s, 3 H, Ac). ^{13}C NMR (101 MHz, Chloroform-*d*) δ 170.59 (carbonyl), 170.10 (carbonyl), 169.83 (carbonyl), 169.65 (carbonyl), 169.57 (carbonyl), 168.60 (carbonyl), 92.57 (C-1β), 89.95 (C-1α), 72.72 (C-3β), 72.67 (C-5β), 70.74 (C-3α), 69.74 (C-4α), 67.81 (C-4β), 67.70 (C-5α), 62.53 (C-2β), 61.36 (C-6α,β), 60.26 (C-2α), 20.98 (Ac), 20.93 (Ac), 20.74 (Ac), 20.72 (Ac), 20.68 (Ac), 20.60 (Ac). LRMS (ESI-Pos) for $C_{14}H_{19}N_3O_9$ (373.11) : $[M+Na]^+$ = 396.10. IR (ATR probe): Azide – 2111.54 cm^{-1} (medium).

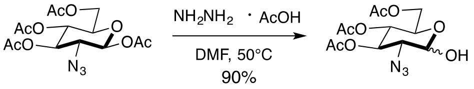

4 5

(2*R*,3*S*,4*R*,5*R*)-2-(acetoxymethyl)-5-azido-6-hydroxytetrahydro-2*H*-pyran-3,4-diyl diacetate (5). 4 (4.25 g, 11.4 mmol, 1.0 eq) and hydrazine acetate (1.26 g, 13.7 mmol, 1.2 eq) was dissolved in 11.7 mL of anhydrous *N*,*N* dimethylformamide under N_2. Reaction warmed to 50°C and continued to stir under N_2 for 20 minutes. TLC 10% EtOAc/DCM confirmed product formation and disappearance of starting material. Reaction was cooled to room temperature, diluted with 12 mL of dichloromethane. The organic layer was washed with deionized water, saturated $NaHCO_3$ and brine. The organic layer was dried over Na_2SO_4, filtered and condensed to yield a yellow oil. The crude product was purified with flash chromatography with a gradient of 100% DCM to 10% EtOAC/DCM to 20% EtOAc/DCM. The purified product was isolated as a colorless oil (3.40 g, 90%). ^{1}H NMR (600 MHz, Chloroform-*d*) (Anomers 1.00α : 0.62β) δ 5.57 – 5.50 (t, 1 H, H-3α), 5.40 (t, *J* = 3.4 Hz, 1 H, (H-1α), 5.06 (t, *J* = 9.7 Hz, 1 H, H-4α), 5.04 – 5.01 (m, 1 H, H-3β), 4.75 (dd, *J* = 7.8, 4.3 Hz, 1 H, H-1β), 4.31 – 4.26 (m, 1 H, H-5α),

4.26 – 4.21 (m, 1 H, H-5β), 4.15 (dd, J = 12.4, 2.3 Hz, 1 H, H-6β), 4.12 (d, J = 10.5 Hz, 1 H, H-6α), 3.74-3.68 (m, 1 H, H-4β), 3.52-3.47 (m, 1 H, H-2β), 3.44 (dd, J = 10.5, 3.2 Hz, 1 H, H-2α), 3.25 (d, J = 3.1 Hz, 1 H, OH), 2.10 (d, J = 2.7 Hz, 9 H, Ac-α), 2.05 (s, 2 H, Ac-β), 2.03 (s, 1 H, Ac-β). ^{13}C NMR (151 MHz, $CDCl_3$) δ 170.71(carbonyl), 170.67 (carbonyl), 170.04 (carbonyl), 170.00 (carbonyl), 169.77 (carbonyl), 169.65 (carbonyl), 96.21(C-1β), 92.14 (C-1α), 72.54 (C-3β), 72.04 (C-4β), 70.43(C-3α), 68.52 (C-4α), 68.31 (C-5β), 67.68 (C-5α), 64.87 (C-2β), 61.96 (C-6β), 61.94 (C-6α), 61.51 (C-2α), 20.75 (CH_3), 20.74 (CH_3), 20.71 (CH_3), 20.68 (CH_3), 20.63 (CH_3), 20.58 (CH_3). LRMS (ESI-Pos) for $C_{12}H_{17}N_3O_8$ (331.10) : $[M+Na]^+$ = 354.10

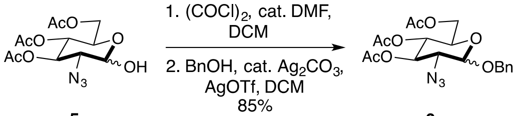

5
6

(2*R*,3*S*,4*R*,5*R*)-2-(acetoxymethyl)-5-azido-6-(benzyloxy)tetrahydro-2*H*-pyran-3,4-diyl diacetate (6). 5 (4.97 g, 15.0 mmol, 1.0 eq) was dissolved in 150 mL of anhydrous dichloromethane under N_2 at room temperature with 4Å activated molecular sieves. Anhydrous DMF (233 μL, 3.00 mmol, 0.2 eq) was added and the reaction stirred for 35 minutes. $(COCl)_2$ (2M, 9.01 mL) was added dropwise and the reaction stirred at room temperature for 1.5 hours. The reaction was filtered, washed with DCM and the solvent was evaporated under reduced pressure without heat. The yellow oil was then coevaporated twice with benzene and dried under high vacuum for 20 minutes. In a separate reaction flask, Ag_2CO_3 (41.4 g, 0.150 mol, 10 eq), AgOTf (0.088 g, 3.45 mmol, 0.23 eq), and anhydrous BnOH (7.77 mL, 75.1 mmol, 5.0 eq) were suspended in 400 mL of anhydrous dichloromethane under N_2 with 4Å molecular sieves. The mixture was cooled to 0°C and stirred for 15 minutes. At the same time, the yellow oily intermediate was dissolved in 170 mL of anhydrous dichloromethane under N_2 with 4Å molecular sieves and stirred at room temperature for 15 minutes. The solution containing the intermediate was added dropwise to the reaction flask. The reaction slowly warmed to room temperature and continued to stir for 15 hours. The reaction mixture was filtered over celite. Product formation was confirmed by LC/MS and TLC (30% EtOAc/Hex). The organic layer was washed three times with deionozed water, dried over Na_2SO_4, filtered and condensed. The resulting residue was purified with flash chromatography 0% to 16% EtOAc in hexanes. Purified product was isolated as a colorless oil (5.39 g, 85%). ^{1}H NMR (600 MHz, Chloroform-*d*) (Anomers 1.00α : 0.31β) δ 7.44 – 7.30 (m, 10 H, aromatic), 5.56 – 5.47 (m, 1 H, H-3α), 5.07 (m, 1 H, H-4α), 5.06 (d, J = 3.6 Hz, 1 H, H-1α), 5.05 – 5.00 (m, 2 H, H-4β, H-3β), 4.98 – 4.93 (m, 1 H, β benzyl methylene), 4.75 (d, J = 11.9 Hz, 1 H, α benzyl methylene), 4.71 (d, J = 11.8 Hz, 1 H, β benzyl methylene), 4.64 (d, J = 11.9 Hz, 1 H, α benzyl methylene), 4.44 (d, J = 8.1 Hz, 1 H, H-1β), 4.26 (dd, J = 12.2, 4.3 Hz, 1 H, H-6β), 4.15 (dd, J = 12.3, 2.2 Hz, 1 H, H-6'α), 4.05 – 4.03 (m, 1 H, H-6α), 4.03 – 4.01 (m, 1 H, H-5α), 3.99 (d, J = 2.2 Hz, 1H, H-6'β), 3.63 (ddd, J = 9.9, 4.7, 2.3 Hz, 1 H, H-5β), 3.56 (dd, J = 10.1, 8.1 Hz, 1 H, H-2β), 3.34 (dd, J = 10.6, 3.6 Hz, 1 H, H-2α), 2.11-2.01 (6s, 12 H, Ac). ^{13}C NMR (151 MHz, Chloroform-*d*) δ 170.58 (carbonyl) , 169.99 (carbonyl), 169.67 (carbonyl), 136.03 (aromatic), 128.63 (aromatic), 128.59 (aromatic), 128.34 (aromatic), 128.27 (aromatic), 128.17 (aromatic), 100.25 (C-

1β), 96.77 (C-1α), 72.52 (H-3β), 71.84 (C-5β), 71.28 (C- β benzyl methylene), 70.48 (C-3α), 70.22 (C- α benzyl methylene), 68.54 (H-4α), 68.45 (H-4β), 67.79 (H-5α), 63.71 (C-2β), 61.93 (C-6β), 61.78 (C-6α), 60.91 (C-2α), 20.75 (CH_3), 20.73 (CH_3), 20.71 (CH_3), 20.69 (CH_3), 20.60 (CH_3), 20.58 (CH_3). LRMS (ESI-Pos) for $C_{19}H_{23}N_3O_8$ (421.15) : $[M+Na]^+$ = 444.14.

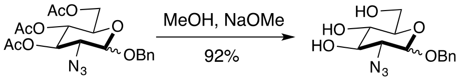

6 7

(2*R*,3*S*,4*R*,5*R*)-5-azido-6-(benzyloxy)-2-(hydroxymethyl)tetrahydro-2*H*-pyran-3,4-diol (7). 6 (2.50 g, 5.93mmol, 1.0 eq) was dissolved in 30 mL anhydrous methanol under N_2. 0.5 M NaOMe (0.7 eq, 8.3mL, from fresh bottle) was added dropwise. Reaction stirred at room temperature for 4 hours. Product formation was determined complete by TLC (10% MeOH/DCM). Reaction quenched with IRA H^+ resin in MeOH until reaction reached pH 4. The resin was filtered and the solvent was condensed under reduced pressure. Product was isolated as a colorless oil (1.67 g, 92%). ^{1}H NMR (600 MHz, Methanol-d_4) (Anomers 1.00α : 0.38β) δ 7.39-7.28 (m, 10 H, aromatic), 4.96 (d, J = 3.5 Hz, 1 H, H-1α), 4.93 (d, J = 11.8 Hz, 1 H, H-β benzyl methylene), 4.76 (d, J = 11.9 Hz, 1 H, H-α benzyl methylene), 4.69 (d, J = 11.8 Hz, 1 H, H-β benzyl methylene), 4.59 – 4.54 (d, J = 11.9 Hz, 1 H, H-α benzyl methylene), 4.40 (d, J = 7.9 Hz, 1 H, H-1β), 3.91 – 3.84 (m, 2 H, H-6β, H-3α), 3.80 (d, J = 12.0 Hz, 1 H, H-6'α), 3.69 (d, J = 5.8 Hz, 2 H, H-6'β, H-6α), 3.65 – 3.61 (m, 2 H, H-5β, H-5α), 3.35 (t, J = 9.3 Hz, 2 H, H-4β, H-4α), 3.24 (d, J = 8.6 Hz, 1 H, H-3β), 3.21 – 3.17 (m, 1 H, H-2β), 3.11 (d, J = 10.1 Hz, 1 H, H-2α). ^{13}C NMR (151 MHz, Methanol-d_4) δ 141.18 (aromatic), 131.93 (aromatic), 131.66 (aromatic), 131.62 (aromatic), 131.43 (aromatic) 131.38 (aromatic), 104.41 (C-1β), 100.95 (C-1α), 80.58 (C-5β), 78.88 (C-3β), 76.68 (C-5α), 75.13 (C-3α), 74.66 (C-4α), 74.42 (C-β benzyl methylene), 74.11, (C-4β), 72.84 (C-α benzyl methylene), 70.79 (C-2β), 67.10 (C-2α), 65.12 (C-6β), 64.98 (C-6α). LRMS (ESI-Pos) for $C_{13}H_{17}N_3O_5$ (295.12) : $[M+Na]^+$ = 318.30.

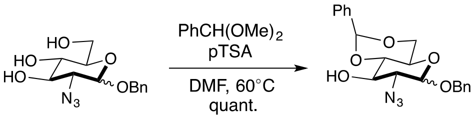

7 8

(2*S*,4a*R*,7*R*,8*R*,8a*S*)-7-azido-6-(benzyloxy)-2-phenylhexahydropyrano[3,2-*d*][1,3]dioxin-8-ol (8). 7 (2.70 g, 9.15 mmol, 1.0 eq), pTSA (0.348g, 1.83 mmol, 0.2 eq), and $PhCH(OMe)_2$ (4.12 mL, 27.5 mmol, 3.0 eq) were dissolved in 22 mL DMF under N_2. The reaction was put under vacuum and heated to 60°C for 1.5 hours. $PhCH(OMe)_2$ (4.12 mL, 27.5 mmol, 3.0 eq) was added to the reaction at 60°C and continued to stir under vacuum for 1.5 hours. TLC (100% DCM) confirmed that the reaction was complete. Once the vacuum was removed and the flask cooled to room temperature, the reaction was quenched with 15 mL of saturated $NaHCO_3$ and stirred for 20 minutes. The reaction was diluted with DCM and extracted three times. The organic layers were combined and

washed three times with 1N HCl. The organic layer was dried over Na_2SO_4, filtered, and condensed to yield a yellow oil. The product was purified with column chromatography (0 to 5% EtOAc in hexanes to 50% EtOAc in hexanes). The clean product was isolated as a colorless oil (quantitative). ^{1}H NMR (600 MHz, Chloroform-*d*) (Anomers 1.00α : 0.49β) δ 7.52 – 7.46 (m, 4 H, aromatic), 7.42 – 7.37 (m, 11 H, aromatic), 7.34 (d, *J* = 7.3 Hz, 2 H, aromatic), 5.55 (s, 1 H, H-β 4,6-benzylidine), 5.54 (s, 1 H, H-α 4,6-benzylidine), 5.00 (d, *J* = 3.7 Hz, 1 H, H-1α), 4.94 (d, *J* = 11.7 Hz, 1 H, H-β benzyl methylene), 4.77 (d, *J* = 12.0 Hz, 1 H, H-α benzyl methylene), 4.71 (d, *J* = 11.7 Hz, 1 H, H-β benzyl methylene), 4.62 (d, *J* = 12.0 Hz, 1 H, H-α benzyl methylene), 4.52 (d, *J* = 8 Hz, 1 H, H-1β), 4.37 (dd, *J* = 10.5, 5.0 Hz, 1 H, H-6β), 4.28 (td, *J* = 9.7, 2.5 Hz, 1 H, H-3α), 4.24 (dd, *J* = 10.3, 4.9 Hz, 1 H, H-6’α), 3.91 (td, *J* = 10.0, 5.0 Hz, 1 H, H-5α), 3.82 (t, *J* = 10.3 Hz, 1 H, H-6’β), 3.74 (t, *J* = 10.3 Hz, 1 H, H-6α), 3.66 (td, *J* = 9.4, 2.5 Hz, 1 H, H-3β), 3.61 – 3.56 (m, 1 H, H-4β), 3.56 – 3.52 (m, 1 H-4α), 3.50 – 3.46 (m, 1 H, H-2β), 3.41 (td, *J* = 9.6, 5.0 Hz, 1 H, H-5α), 3.31 (dd, *J* = 10.0, 3.7 Hz, 1 H, H-2α), 2.67 (d, *J* = 2.6 Hz, 1 H, 3-OH α), 2.65 (d, *J* = 2.6 Hz, 1 H, 3-OH β). ^{13}C NMR (151 MHz, Chloroform-*d*) δ 162.53 (DMF), 137.00 (aromatic), 126.92 (aromatic), 136.54 (aromatic), 136.39 (aromatic), 129.29 (aromatic), 128.55 (aromatic), 128.44 (aromatic), 128.33 (aromatic), 128.22 (aromatic), 128.19 (aromatic), 128.16 (aromatic), 128.10 (aromatic), 128.07 (aromatic), 128.00 (aromatic),126.67 (aromatic), 126.29 (aromatic), 125.29 (aromatic), 103.20 (C-β 4,6-benzylidine), 102.06 (C-α 4,6-benzylidine), 101.13 (C-1β), 97.60 (C-1α), 81.99 (C-4α), 80.77 (C-4β), 72.00 (C-3β), 71.45 (C-β benzyl methylene), 69.91 (C-α benzyl methylene), 68.82 (C-3α), 68.73 (C6-α), 66.66 (C-2β), 66.27 (C-5β), 63.26 (C-6β), 62.68 (C-5α), 52.73 (C-2α), 36.47 (DMF), 31.43 (DMF). LC/MS (ESI-Pos) for $C_{20}H_{21}N_3O_5$ (383.15) : $[M+1]^+$ = 384.15 and $[2M+Na]^+$ = 789.30.

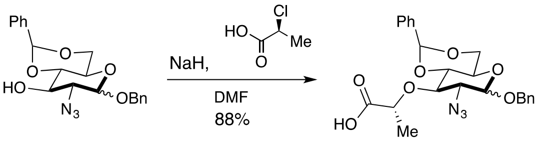

8 9

(*R*)-2-(((2*S*,4a*R*,7*R*,8*R*,8a*S*)-7-azido-6-(benzyloxy)-2-phenylhexahydropyrano[3,2-*d*][1,3]dioxin-8-yl)oxy)propanoic acid (9). **8** (2.1448 g, 5.6 mmol, 1.0 eq) was dissolved in 30 mL of DMF under N_2. NaH (60% in oil, 1.399g, 35.0 mmol, 6.25 eq) was added slowly and reaction stirred at room temperature under N_2 for 30 minutes. To the reaction was added (*S*)-(-)-2-chloropropionic acid (2.43 mL, 28.0 mmol, 5.0eq), with H_2 gas evolution observed. The reaction stirred for 30 minutes then a second addition of NaH (60% in oil, 1.399 g, 35.0 mmol, 6.25 eq) was added. The reaction stirred at room temperature for 16 hours. The reaction was quenched slowly with deionized water at 0°C. The pH of the solution was brought to 4 with the addition of 1N HCl. A gummy brown solid formed in the reaction flask. The aqueous liquid was decanted off and passed through a fine filter funnel. Any brown residue in the frit was dissolved in EtOAc and combined with the solid in the reaction flask. The aqueous filtrate was extracted three times with EtOAc. All organic solutions were combined and washed with 1N HCl, dried

over Na_2SO_4, filtered and condensed to yield a brown oily solid. The residue was purified by flash chromatography with a gradient of 2.5% MeOH/DCM with 0.01% AcOH to 5% MeOH/DCM with 0.01% AcOH. Isolated product as a light yellow foam (2.25 g, 88%). ^{1}H NMR (600 MHz, Chloroform-*d*) (Anomers 1.00α : 0.43β) δ 8.03 (s, 1 H, DMF), 7.43 (td, *J* = 7.8, 3.7 Hz, 5 H, aromatic), 7.41 – 7.38 (m, 14 H, aromatic), 7.36 – 7.33 (m, 3 H, aromatic), 5.56 (2s, *J* = 5.3 Hz, 2 H, H-β 4,6-benzylidine and H-α 4,6-benzylidine), 5.08 (d, *J* = 3.7 Hz, 1 H, H-1α), 4.94 (d, *J* = 11.6 Hz, 1 H, H-β benzyl methylene), 4.76 (d, *J* = 11.8 Hz, 1 H, H-α benzyl methylene), 4.71 (d, *J* = 11.6 Hz, 1 H, H-β benzyl methylene), 4.63 (d, *J* = 11.8 Hz, 1 H, H-α benzyl methylene), 4.57 (d, *J* = 8.0 Hz, 1 H, H-1β),4.51 – 4.42 (m, 2 H, H-muramic acid α, H-muramic acid β), 4.39 (dd, *J* = 10.5, 5.1 Hz, 1 H-6β), 4.23 (dd, *J* = 10.3, 4.9 Hz, 1 H, H-6'α), 4.02 (t, *J* = 9.5 Hz, 1 H, H-3α), 3.90 (td, *J* = 10.0, 4.9 Hz, 1 H, H-5α), 3.83 (t, *J* = 10.3 Hz, 1 H, H-6'β), 3.75 (t, *J* = 10.3 Hz, 1 H, H-6α), 3.69 (t, *J* = 9.2 Hz, 1 H, H-6β), 3.67 – 3.61 (m, 2 H, H-4β, H-4α), 3.56 (dd, *J* = 9.7, 8.1 Hz, 1 H, H-2β), 3.45 – 3.38 (m, 3 H, H-2α, H-5β and H-3β), 2.17 (s, 1 H, acetone), 1.47 (d, *J* = 6.9 Hz, 4H, CH_3 muramic acid α and β). ^{13}C NMR (151 MHz, Chloroform-*d*) δ 174.60 (carbonyl), 162.73 (carbonyl), 136.76 (aromatic), 136.05 (aromatic), 129.29 (aromatic), 129.22 (aromatic), 128.64 (aromatic), 128.39 (aromatic), 128.35 (aromatic), 128.27 (aromatic), 125.85 (aromatic), 125.80 (aromatic), 101.60 (C-α 4,6-benzylidine), 101.52 (C-β 4,6-benzylidine), 101.04 (C-1β), 96.86 (C-1α), 82.36 (C-4α), 81.32 (C4-β), 78.90 (C-3β), 76.58 (C-3α) , 76.29 (C-muramic acid) , 71.56 (C-β benzyl methylene), 70.08 (H-α benzyl methylene), 68.74 (C-6α), 68.44 (C-6β), 66.18 (C-5β), 64.83 (C-2β), 62.69 (C-5α), 62.26 (C-2α), 36.56 , 31.51 , 30.93 , 18.96 (C-CH_3 muramic acid). LRMS (ESI-Neg) for $C_{23}H_{25}N_3O_7$ (455.17) : $[M-H]^-$ = 454.15.

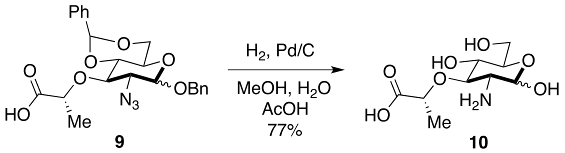

(*R*)-2-(((3*R*,4*R*,5*S*,6*R*)-3-amino-2,5-dihydroxy-6-(hydroxymethyl)tetrahydro-2*H*-pyran-4-yl)oxy) propanoic acid (10). 9 (0.987 g, 2.17 mmol, 1.0 eq) was suspended in a solution of 62 mL H_2O, 46 mL MeOH and 7.7 mL AcOH under N_2. Pd/C (0.950 g) was added to the reaction under N_2 and then a hydrogen atmosphere was introduced. The reaction stirred at room temperature under H_2 for 16 hours. LC/MS ESI pos m/z (M+H) 252 confirmed product formation. The H_2 was removed and the Pd was filtered over celite and rinsed with MeOH, then water, being careful not to dry out the Pd. The filtrate was condensed, dissolved in DI H_2O 0.1% formic acid (50mg/mL) and purified on the Waters preparative HPLC/MS with the method as follows: flow rate 20 mL/min, 0.1% formic acid in millipure H_2O as eluent A and 0.1% formic acid in HPLC grade acetonitrile as eluent B. Inlet file (A/B): 0-2 min 95/5, 2.5 min 20/80, 3-4 min 95/5. The product was collected based on $[M+H]^+$ 252 with a retention time between 1.5-2.25 min. The appropriate fractions were combined and lyophilized to give a white powder (0.350 g, 65% yield, 77% highest reported yield). ^{1}H NMR (600 MHz, Methanol-d_4) (Anomers 1.00α : 0.61β) δ 5.30 (d, *J* = 3.5 Hz, 1 H, H-1α), 4.77 (d, *J* = 8.4 Hz, 1 H, H-1β), 4.42 – 4.38 (m, 1 H, H-muramic acid β), 4.38 – 4.34 (m, 1 H, H-muramic acid α), 3.85 (dd, *J* =

11.9, 2.1 Hz, 1 H, H-6β), 3.79 (t, *J* = 2.5 Hz, 1 H, H-5α), 3.78 – 3.77 (m, 2 H, 6'α, 5α), 3.72 (dd, *J* = 12.3, 5.5 Hz, 1 H, 6α), 3.68 (dd, *J* = 11.9, 5.7 Hz, 1 H, H-6'β), 3.63 – 3.58 (m, 1 H, H-5β), 3.50 (d, *J* = 9.3 Hz, 1 H, H-3α), 3.48 (d, *J* = 2.8 Hz, 1 H, H-4α), 3.46 (d, *J* = 8.7 Hz, 1 H, H-4β), 3.37 – 3.33 (m, 1 H, H-3β), 3.07 (dd, *J* = 10.5, 3.6 Hz, 1 H, H-2α), 2.79 (dd, *J* = 10.6, 8.4 Hz, 1 H, H-2β), 1.46 – 1.38 (m, 6H, CH_3 muramic acid α and β). ^{13}C NMR (151 MHz, Methanol-d_4) δ 97.75 (C-1β), 93.25 (C-1α), 84.09 (C-3β), 82.18 (C-3α), 82.05 (C-muramic acid β), 81.98 (C-muramic acid α), 80.75 (C-4β), 75.84 (C-5α), 75.27 (C-5β), 75.02 (C-4α), 64.77 (C-6β), 64.55 (C-6α), 60.33 (C-2β), 57.97 (C-2α), 22.63 (C-CH_3 muramic acid α), 22.60 (C-CH_3 muramic acid β). HRMS (ESI-Pos) for $C_9H_{17}NO_7$ (251.100504) : $[M+H]^+$ = 252.10712 (theoretical $[M+H]^+$: 252.10778).

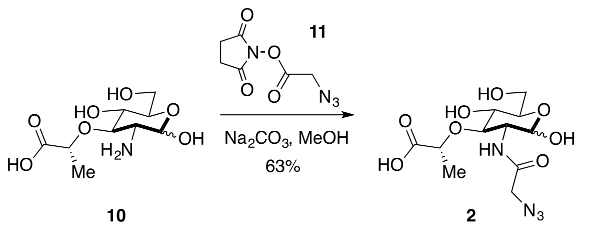

(*R*)-2-(((3*R*,4*R*,5*S*,6*R*)-3-(2-azidoacetamido)-2,5-dihydroxy-6-(hydroxymethyl)tetrahydro-2*H*-pyran-4-yl)oxy)propanoic acid (2). A: Preparation of 2,5-dioxopyrrolidin-1-yl 2-azidoacetate (**11**): 2-azidoacetic acid (488 μL, 6.52 mmol, 1.0 eq) was dissolved in 13 mL of anhydrous DMF under N_2. *N*-hydroxysuccinimide (0.788 g, 6.85 mmol, 1.05 eq) was added to the reaction followed by *N*-(3-dimethylaminopropyl)-*N'*-ethylcarbodiimide hydrochloride (EDC) (1.50 g, 7.82 mmol, 1.2 eq. The reaction turned yellow and stirred under N_2 at room temperature for 14 hours. The reaction was concentrated, quenched with deionized water and extracted three times with EtOAc. The organic layers were combined and washed three times with 1N HCl, three times with saturated $NaHCO_3$, and brine. The organic layer was dried over Na_2SO_4, filtered and condensed to yield a tan solid. The solid was washed three times with anhydrous Et_2O and dried under high vacuum to yield a white solid (0.624 g, 50%). ^1H NMR (600 MHz, Chloroform-*d*) δ 4.24 (s, 2H, CH_2 before Az), 2.88 (s, 4H, CH_2 NHS). ^{13}C NMR (151 MHz, Chloroform-*d*) δ 168.38 (carbonyl), 164.13 (carbonyl), 48.00 (CH_2), 25.57 (CH_2 NHS). IR (ATR probe): Azide – 2108.07 cm^{-1} (medium).

To **10** (0.089 g, 0.35 mmol, 1.0 eq) and Na_2CO_3 (0.250 g, 2.36 mmol, 6.7 eq) was added 8 mL of anhydrous MeOH under N_2. 2,5-dioxopyrrolidin-1-yl 2-azidoacetate (0.186 g, 0.94 mmol, 2.7 eq) was added in two additions every 30 minutes. Reaction was monitored by TLC (25% MeOH/EtOAc) and LC/MS ESI neg $[M-1]^-$ = 333. Once complete, the reaction was filtered and evaporated under reduced pressure without heat. The off-white solid was purified on the Waters preparative HPLC/MS. Crude oil was dissolved in DI H_2O 0.1% formic acid (50mg/mL) and purified on the Waters preparative HPLC/MS with the method as follows: flow rate 20 mL/min, 0.1% formic acid in millipure H_2O as eluent A and 0.1% formic acid in HPLC grade acetonitrile as eluent B. Inlet file (A/B): 0 min 95/5, 4 min 5/95, 4.5 min 5/95 and 4.8-5 min 95/5. The product was collected based on $[M-H]^-$ ESI neg 333.2 with a retention time between 1.25-1.75 min. The appropriate fractions were combined and lyophilized to give an off white

crystalline solid (0.073 g, 63% yield). ^{1}H NMR (600 MHz, Methanol-d_4) (Anomers 1.00α : 0.20β) δ 5.39 (d, *J* = 3.0 Hz, 1 H, H-1α), 4.70 (q, *J* = 7.0 Hz, 1 H, H-muramic acid α), 4.64 (d, *J* = 8.2 Hz, 1 H, H-1β), 4.58 (q, *J* = 6.9 Hz, 1H, H-muramic acid β), 3.93 (s, 2 H, CH_2 methylene), 3.78 (t, *J* = 2.7 Hz, 1 H, H-3α), 3.76 (d, *J* = 2.5 Hz, 1 H, H-6α), 3.74 – 3.71 (m, 1 H, H-4α), 3.70 – 3.69 (m, 1 H, H-6'α), 3.67 (d, *J* = 3.2 Hz, 1 H, C-2α), 3.63-3.58 (m, 1 H, H-2β), 3.53 (t, J = 9.0 Hz, 1 H, H-5α), 3.46 (t, J = 9.2 Hz, 1 H, H-3β), 1.43 (d, *J* = 7.0 Hz, 3 H, CH_3 muramic acid α), 1.40 (s, 1 H, CH_3 muramic acid β). ^{13}C NMR (151 MHz, Methanol-d_4) δ 94.16 (C-1α) , 81.20 (C-4α), 79.10 (CH-muramic acid α), 76.03 (C-3α), 75.69 (C-5α), 64.96 (C-6α), 58.46 (C-2α), 55.72 (CH_2 methylene α), 21.86 (CH_3 muramic acid α). HRMS (ESI-Neg) for $C_{11}H_{18}N_4O_8$ (334.112466) : $[M-H]^-$ = 333.10550 (theoretical $[M-H]^-$: 333.10519). IR (ATR probe): Azide – 2120.20 cm^{-1} (medium).

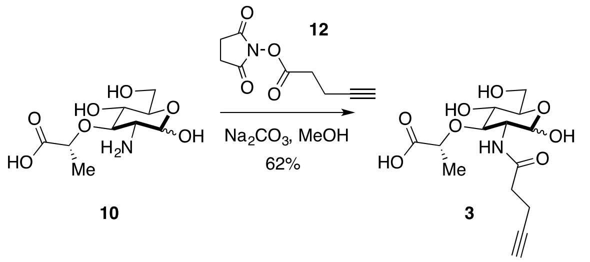

10 3

(2*R*)-2-(((3*R*,4*R*,5*S*,6*R*)-2,5-dihydroxy-6-(hydroxymethyl)-3-(pent-4-ynamido)tetrahydro-2*H*-pyran-4-yl)oxy)propanoic acid (3). A: Preparation **2,5-dioxopyrrolidin-1-yl pent-4-ynoate** (**12**): 4-pentynoic acid (0.250 g, 2.74 mmol, 1.0 eq) was dissolved in 6.37 mL of anhydrous DMF under N_2. *N*-hydroxysuccinimide (0.331 g, 2.88 mmol, 1.05 eq) was added to the reaction followed by *N*-(3-dimethylaminopropyl)-*N'*-ethylcarbodiimide hydrochloride (EDC) (0.631 g, 3.29 mmol, 1.2 eq. The reaction stirred at room temperature under N_2 for 14 hours. The reaction was concentrated, quenched with deionized water and extracted three times with EtOAc. The organic layers were combined and washed three times with 1N HCl, three times with saturated $NaHCO_3$, and brine. The organic layer was dried over Na_2SO_4, filtered and condensed to yield a tan solid. The solid was washed three times with anhydrous Et_2O and dried under high vacuum to yield a white solid (0.2997g, 57%). ^{1}H NMR (600 MHz, Chloroform-*d*) δ 2.90 – 2.87 (m, 2 H, CH_2 methylene adjacent to amide), 2.85 (s, 4 H, CH_2 NHS), 2.62 (td, *J* = 7.6, 2.6 Hz, 2 H, CH_2 methylene adjacent to alkyne), 2.05 (t, *J* = 2.6 Hz, 1 H, H-alkyne). ^{13}C NMR (151 MHz, $CDCl_3$) δ 168.89 (carbonyl), 167.01 (carbonyl), 80.85 (C-quat), 70.04 (C-H alkyne), 30.32 (CH_2 methylene adjacent to amide), 25.58 (CH_2 NHS), 14.10 (CH_2 methylene adjacent to alkyne).

To **10** (0.020 g, 0.080 mmol, 1.0 eq) and Na_2CO_3 (0.0566 g, 0.534 mmol, 6.7 eq) was added 1.1 mL of anhydrous MeOH under N_2. **12** (0.0417 g, 0.214 mmol, 2.7 eq) was added in two additions every 30 minutes. Reaction was monitored by TLC (25% MeOH/EtOAc) and LC/MS. Once complete, the reaction was filtered and evaporated under reduced pressure without heat. The off-white solid was purified on the Waters preparative HPLC/MS with the following method: Crude product was dissolved in DI H_2O 0.1% formic acid (20mg/mL) and purified on the Waters preparative HPLC/MS

with the method as follows: flow rate 20 mL/min, 0.1% formic acid in millipure H_2O as eluent A and 0.1% formic acid in HPLC grade acetonitrile as eluent B. Inlet file (A/B): 0 min 95/5, 4 min 5/95, 4.5 min 5/95 and 4.8-5 min 95/5. The product was collected based on $[M-H]^-$ ESI neg 330.2 with a retention time between 1.50-1.80 min. The appropriate fractions were combined and lyophilized to give an off white crystalline solid (15.7 mg, 62% yield). ^{1}H NMR (600 MHz, Methanol-d_4) (Anomers ~90% α) δ 5.43 (d, J = 3.0 Hz, 1 H, H-1α), 4.58 (q, J = 6.8 Hz, 1 H, CH muramic acid α), 4.55-4.52 (m, 1 H, H-1 β, CH muramic acid β), 3.85-3.80 (m, 1 H, H-6β), 3.79-3.73 (m, 2 H, H-6α, H-4α), 3.73-3.65 (m, 2 H, H-3α, H-6'α), 3.61-3.58 (m, 1 H, H-2α), 3.49 (t, J = 9.1 Hz, 2 H, H-5α, H-2β), 3.47-3.44 (m, 1 H, H-5β), 2.68 (d, J = 3.3 Hz, 2 H, CH_2 adjacent to amide), 2.55-2.46 (m, 4 H, CH_2 adjacent to alkyne α/β), 2.22 (d, J = 6.6 Hz, 2 H, CH alkyne α/β), 1.46 -1.36 (m, 5 H. CH_3 muramic acid α/β).^{13}C NMR (151 MHz, Methanol-d_4) δ 182.73 (carbonyl), 177.42 (carbonyl), 176.80 (carbonyl), 101.09 (C-1β), 94.22 (C-1α), 86.19 (C-quat alkyne α), 86.08 (C-quat alkyne β), 83.77 (C-3β), 80.98 (C-3α), 80.52 (C-4β), 80.17 (CH muramic acid α), 76.03 (C-4α), 75.60 (C-5α), 75.01 (C-5β), 72.64 (CH alkyne β), 72.57 (CH alkyne α), 65.09 (C-6α,β), 60.92 (C-2β), 58.57 (C-2α), 38.77 (CH_2 adjacent to amide β), 28.82 (CH_2 adjacent to amide α), 22.37 (CH_3 muramic acid β), 22.24 (CH_3 muramic acid α), 18.09 (CH_2 methylene adjacent to alkyne α), 18.02 (CH_2 methylene adjacent to alkyne β). HRMS (ESI-Neg) for $C_{14}H_{21}NO_8$ (331.126719) : $[M-H]^-$ = 330.11907 (theoretical $[M-H]^-$: 330.11944).

Supplementary References:

1 Baba, T. *et al.* Construction of Escherichia coli K-12 in-frame, single-gene knockout mutants: the Keio collection. *Mol. Syst. Biol.* **2**, 1-11 (2006).

2 Konkol, M. A., Blair, K. M. & Kearns, D. B. Plasmid-encoded ComI inhibits competence in the ancestral 3610 strain of Bacillus subtilis. *J. Bacteriol.* **195**, 4085-4093 (2013).

3 Ye, H. *et al.* A safe and facile route to imidazole-1-sulfonyl azide as a diazotransfer reagent. *Org. Lett.* **15**, 18-21 (2013).
